# ‘Science Fun Days’: Opportunities for Connecting Primary School Pupils With Nature and Microbiology

**DOI:** 10.1111/1751-7915.70279

**Published:** 2025-12-10

**Authors:** Elizabeth J. Archer, Corinne B. Whitby, Robert M. W. Ferguson, Dave R. Clark, Benjamin M. Skinner, Drew K. Henderson, Olivia S. Solanke, Anna M. Sturrock, Rebekah Boreham, Terry J. McGenity

**Affiliations:** ^1^ School of Life Sciences University of Essex Colchester UK; ^2^ Communications and External Relations University of Essex Colchester UK

## Abstract

Microbes are essential for the functioning of life on earth, yet a lack of awareness of their positive activities persists in society. In the UK, microbiology is scarcely taught before secondary education. Therefore, we organised ‘Science Fun Days’ for primary school pupils (aged 9–11 years) in 2024 and 2025, with the aims of increasing their microbiological awareness and, more generally, promoting positive attitudes towards science and nature. Over 450 pupils attended a Science Fun Day hosted at the University of Essex, which involved hands‐on activities in the laboratory and outdoors. Pre‐event and post‐event surveys were completed by 307 and 305 of these pupils, respectively, from across seven schools. The surveys revealed that, after participating in a Science Fun Day, the proportion of pupils who would like a job in science increased from 29.6% to 41.9% in 2024 and 21.8% to 32.9% in 2025. Pupils from schools located in areas of high deprivation rated their desire for a science career significantly higher overall than pupils from schools located in low deprivation areas. Surveys also captured a post‐event increase in the percentage of pupils that know what microbes are from 68.7% to 88.0% in 2024 and 49.3% to 79.1% in 2025. Gender differences were minimal and included higher overall perceived confidence in science lessons by male‐identifying students; however, female‐identifying students reported similar levels of confidence as their male‐identifying peers in the post‐event survey. Our results support the value of extra‐curricular excursions to boost children's understanding of microbiology, enable positive attitudes towards science, and encourage science‐related career aspirations.

## Graphical abstract

This article details ‘Science Fun Day’ outreach events which we organised for primary school pupils in Essex, UK. We demonstrate that pupils ended the event with increased enjoyment of learning about science, increased desire to have a career in science and a greater understanding of the importance of microbes.

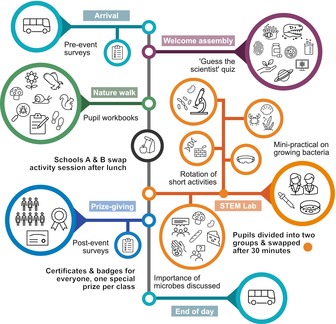

## 1 Introduction

We have reached an era in Earth's history that is defined by the destructive forces of human activity, the Anthropocene (Ellis 2015). In this time of increasingly complex global crises, it has never been more pressing to encourage the scientific literacy and nature connectedness of society, thereby promoting awareness and stewardship of the planet's finite resources. Whilst often forgotten, microbes underpin the functioning of healthy environments and all organisms, including humans, by recycling nutrients, degrading pollutants, enhancing food security, aiding food digestion, generating a significant amount of the oxygen we breathe and much more (Cavicchioli et al. 2019; Crowther et al. 2024). Therefore, an understanding of the essential positive activities of microbes in everyday life and wider planetary health—microbiology literacy—is especially needed amongst the general population and decision makers (Timmis et al. 2019, 2024). Indeed, more time spent in nature has been associated with positive attitudes towards microbes (Robinson et al. 2021) as well as better mental health and well‐being (Barton and Rogerson 2017; Wood et al. 2023). Several studies have shown that time spent in nature during childhood often shapes connectedness with living organisms and pro‐environmental attitudes in adult life (Rosa et al. 2018; Fretwell and Greig 2019; Chawla 2020; Cleary et al. 2020). Promoting children's aspirations to study science subjects helps to secure a future generation of scientists, who can contribute to a sustainable future across all sectors such as health, food and the economy. Equally, a more scientifically literate society is likely to make more rational decisions on issues as diverse as vaccination and climate protection, contributing to a healthier and more equitable society (Archer et al. 2012; Timmis et al. 2024). School excursions to local venues, such as academic institutions, are one way to foster greater environmental awareness and scientific (including microbial) literacy, providing cost‐effective, fun, hands‐on activities to enrich learning (McGenity et al. 2020).

In the United Kingdom, children's formal education is shaped by the National Curriculum. Although important biological concepts are taught from a young age, natural history and the diversity of local plants and animals are not always fully explored or experienced first‐hand. Microbiology content is even more scarce, often omitting the fundamental importance of microbes for a functioning biosphere and their profound benefits to health. The first and only mention of microbes' contribution to ecosystem health (as decomposers) is included in the Key Stage 4 programme of study, when pupils are aged 14–16 (Department for Education 2015). With few exceptions, young children across the globe receive little information about the vital roles of microbes. Microbiology education is therefore needed from a young age to provide a balanced and positive understanding of microbes to counter the prevailing negative perception of microbes as ‘germs’, which may have been exacerbated by the COVID‐19 pandemic (Timmis et al. 2019, 2024; McGenity et al. 2020; Robinson et al. 2021; Timmis 2023a, 2023b). This ‘germaphobia’ or ‘microbiophobia’ is concerning as it discourages interaction with ‘dirty’ environments (Timmis et al. 2019; Robinson et al. 2021) that bring benefits to health and well‐being. It is also important to expand an awareness of microbes beyond their ability to cause disease—only around one in every billion of the estimated 1 trillion microbial species on Earth is a human pathogen (‘Microbiology by numbers,’ 2011; Locey and Lennon 2016)—because the impact of policy decisions related to microbes and microbiology will influence our efforts to make progress on many, if not all, United Nations Sustainable Development Goals (SDGs) (Timmis et al. 2017; Akinsemolu 2018; Crowther et al. 2024; Peixoto et al. 2024). Consequently, increasing microbiology literacy must be a priority.

Many children do not get exposure to science, for example by knowing a scientist, participating in educational recreation such as visits to a science museum, or having access to at‐home science kits—so‐called ‘science capital’ (Archer et al. 2015). This is of particular concern in areas of high socioeconomic deprivation as the low ‘cultural capital’ of Year 6 primary school pupils (based on parental education, number of books in the home and frequency of museum visits) has been linked to lower science career aspirations (DeWitt and Archer 2015). Therefore, schools play a crucial role in democratising extra‐curricular engagement with science by providing excursions to different facilities, institutes, museums and galleries, which take learning out of the classroom and into the wider world (McGenity et al. 2020). The alternative style of learning experienced by children during school excursions uses different skills and senses, which can encourage the most reserved pupils to be actively involved. Academic environments are well suited to hosting schools in this way, thanks to their enthusiastic and knowledgeable personnel and student‐friendly teaching facilities (McGenity et al. 2020). Many universities also have an outreach team to facilitate public engagement with the community. As well as providing a unique learning experience, school visits to universities may highlight future higher‐education opportunities that were previously unknown to pupils. Although deciding on a specific subject to study may be a distant priority, it is vital to ensure that pupils are aware of the future options open to them, especially those pupils who might not have considered higher education or careers benefiting from scientific/green skills before the visit. Gender stereotypes, such as intellectual ability, that influence science aspirations can begin to develop at primary school age (Bian et al. 2017); therefore interaction with a diverse team of scientists during such visits can increase pupils' exposure to science students and professionals that they can relate to.

**FIGURE 1:**
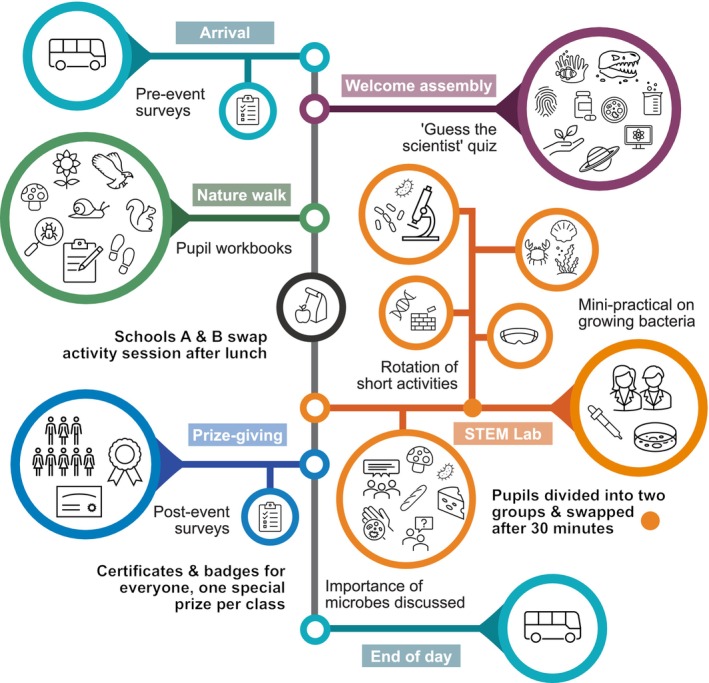
Overall structure of a Science Fun Day hosting two schools.

Our first aim was to design informative and enjoyable events for children aged between 9 and 11 years that combined classroom, laboratory and field activities, and to introduce microbes, their activities and interactions, along with methods to study them. The events, branded as ‘Science Fun Days’ (Figure 1), were not solely about microbes. Instead, we wove microbial examples into and between aspects of biology and ecology with which school children were more familiar. The second aim was to obtain feedback from teachers, and to evaluate changes in pupils' attitudes towards science and microbes/microbiology after the events. These responses were also explored to assess whether answers differ based on the socioeconomic status of the school location or gender identity of pupils to better understand the impact of the events on specific groups of pupils. Multiple Science Fun Days were carried out in 2024 and 2025, and our final goal was to implement any learnings from the 2024 events and produce guidance for others planning to run similar events.

## 2 Experimental Procedures

### 2.1 Science Fun Day Structure

In celebration of the 2024 and 2025 British Science Weeks, the University of Essex hosted 465 Year 4–6 pupils (9–11 years old) from eight primary schools across Essex for Science Fun Days that were packed with educational activities. Schools from low‐income areas geographically close to the University and within the Tendring District Council boundary were prioritised to attend (Schools 1, 4, 5 and 7). Four additional schools that need not meet the criterion of being from a low‐income area were invited to attend as part of their ongoing participation with the InChildHealth EU project. Across the 2 years of events, ten groups of pupils from eight different schools attended. In 2024, groups of pupils from Schools 1, 2 and 3 attended and completed surveys whilst two further school groups (School 4 and an additional school) attended but did not take part in the pupil surveys. In 2025, groups of pupils from Schools 3, 4, 5, 6 and 7 attended and all groups took part in the pupil survey. Where the same school attended in both years, different pupils took part in the event.

Each day was designed for two groups of ~60 students and was structured as outlined in Figure 1 and below. School group sizes varied between 19 and 62 (median 59) and smaller school groups took part in activities together to make up a collective group of ~60 pupils. We conducted a risk assessment and shared this with teachers prior to the event in an information pack.

### 2.2 Arrival

#### 2.2.1 Pre‐Event Survey

Ethical approval was gained to conduct pupil surveys from an institutional ethics review committee via the University of Essex Ethics Review and Management System (ERAMS) (ETH2324‐0242 and ETH2224‐0118). Survey questions included asking pupils to rate their enjoyment of learning about science and nature, their desire to have a job in science, how much they would like to go to university when they are older and how much they know about microbes (see Data S2 and S4 in Supporting Information). Pupils were welcomed to our campus by Student Ambassadors (paid university students who are trained to look after visitors and serve as role models for the pupils). One Student Ambassador was assigned per 15 school pupils. For those pupils who had consent from a parent/guardian and completed a pupil assent form, the pre‐event survey was conducted to assess the baseline attitudes and scores of the pupils so that they could later be compared to the results of the post‐event survey obtained at the end of the day.

### 2.3 Welcome Assembly

#### 2.3.1 Interactive Scientific Careers Quiz

To get the pupils excited about the day ahead, we began with a short interactive introduction (see Figure S1). Pupils were asked what they thought a scientist looks like to highlight the common biased perception of an older man in a lab coat, which was exemplified by the results of an internet image search of the term ‘scientist’. A variety of different scientific careers were then highlighted to the pupils with a ‘guess the scientist’ quiz.

At the end of this assembly, the two school groups were sent to their first activity session—either an outdoor nature walk or activities in the Science Technology Engineering and Mathematics (STEM) Teaching Laboratory that were happening concurrently, before swapping after lunch to complete their remaining session.

### 2.4 Laboratory Activities

#### 2.4.1 Introductory Talk

Pupils were provided with a laboratory coat (sourced from Sublime Science Labs Ltd), which was not necessary for safety reasons, but made them feel like professional scientists when they entered the STEM Teaching Laboratory. Then, pupils were given a short talk on microbes and their importance in everyday life with the aim of counteracting the negative bias towards microbes as solely ‘germs’ that cause disease. The essential role of microbes in the creation of popular foods such as chocolate, cheese and pickles was emphasised, and pupils had to guess how many microbes they host in their microbiome. Microbe‐themed plush toys, such as a *Penicillium* sp. and a bacteriophage, were shown to the pupils as props to visualise microbes. To reduce group size, pupils were then allocated to two groups to take part in a mini‐practical or rotation of short activities, swapping midway to complete their second activity.

#### 2.4.2 Microbiology Practical

**FIGURE 2:**
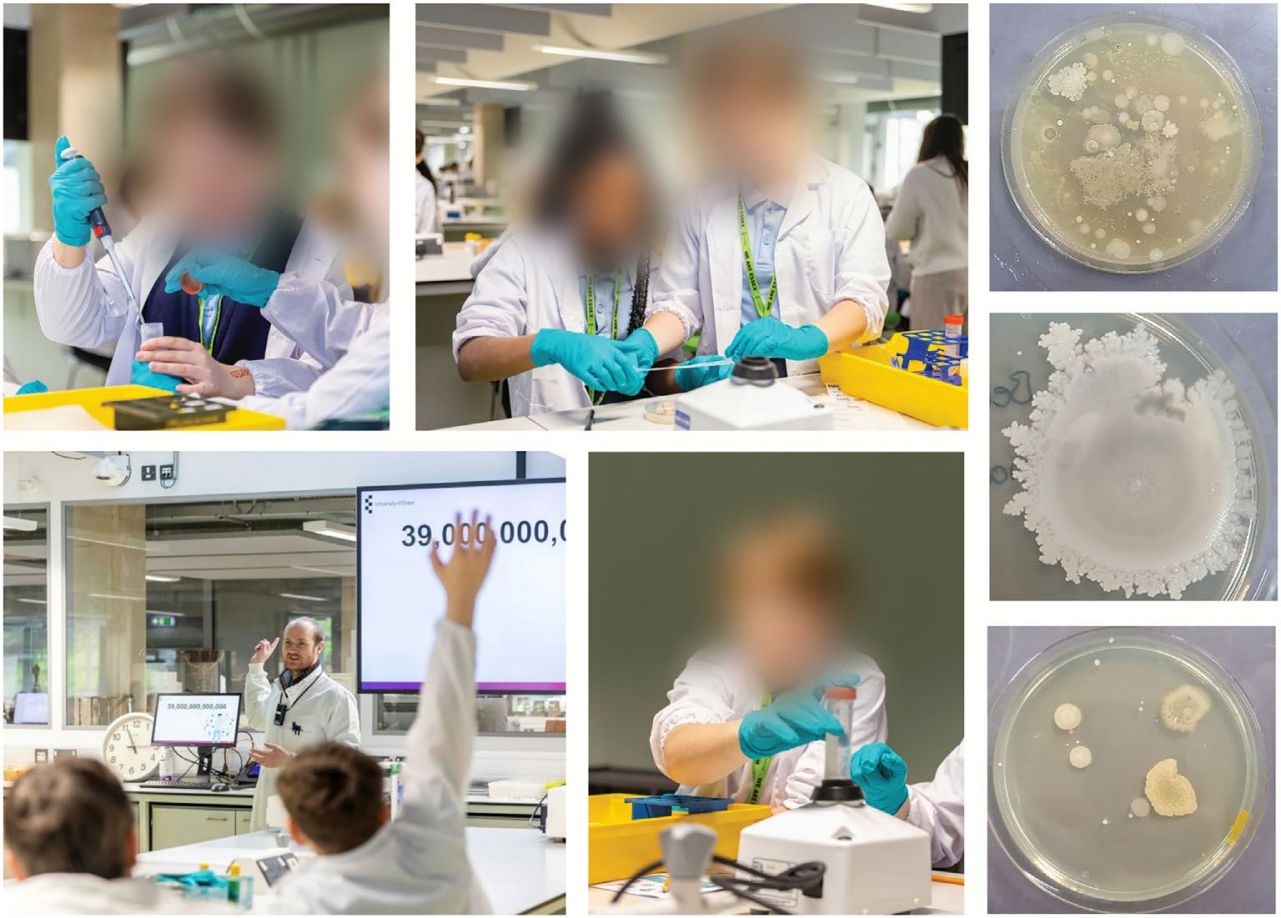
Pupils took part in activities in the University of Essex STEM Teaching Laboratory, including a ‘growing microbes’ practical, after a short introductory talk with Dr. Rob Ferguson on why microbes are important. Photos (examples on the right) were sent to pupils after the event so that they could view and compare the diversity of colonies grown from their samples.

To demonstrate the ubiquity of microbes in the environment, pupils were able to experience culturing of microbes from samples that they collected. To do this, pupils were tasked with finding an area of the Teaching Laboratory that might harbour microbes and therefore be a good location to swab for microbial growth. This goal prompted them to think about factors that would promote microbes' growth and persistence, such as moisture (plug holes were a popular choice) and high‐touch surfaces such as door handles. With supervision and working in pairs, pupils first swabbed their selected microenvironment using electrostatic dust cloths (Swiffer UK) approximately 2 × 10 cm in size. They then extracted microbes by placing the cloth into a 50 mL centrifuge tube containing 10 mL sterile deionised water and vortexing for 1 min (the vortexing was done as a group activity with a countdown from the lead scientist) (see Figure 2). A 100 μL aliquot of the extract was pipetted (by the students) onto a plate containing LB agar, which was then labelled, sealed with PetriFilm, and incubated at the University at 37°C. Images of the plates were sent to the schools later that week so that pupils could see the diversity of their microbial cultures and discuss their findings. This is an adapted version of a passive air sampling protocol we typically follow, which employs electrostatic dust collectors, and was used as training for students who went on to take their own classroom air samples in 2025 (see ‘Classroom Bioaerosol sampling’ in Supporting Information document Appendix S1 and Data S5 for details).

#### 2.4.3 Rotation of Short Activities

Several 5‐min activities were also set up on the other side of the laboratory, which small groups of pupils rotated around during the session. Pupils were given a printed booklet corresponding with the day's activities. Completing the tasks in the booklet kept pupils engaged and provided a take‐home reminder of the Science Fun Day (see Data S6 in Supporting Information). Examples of short activities are outlined below.

### 2.5 Observing Bacteria and Fungi Under the Microscope

**FIGURE 3:**
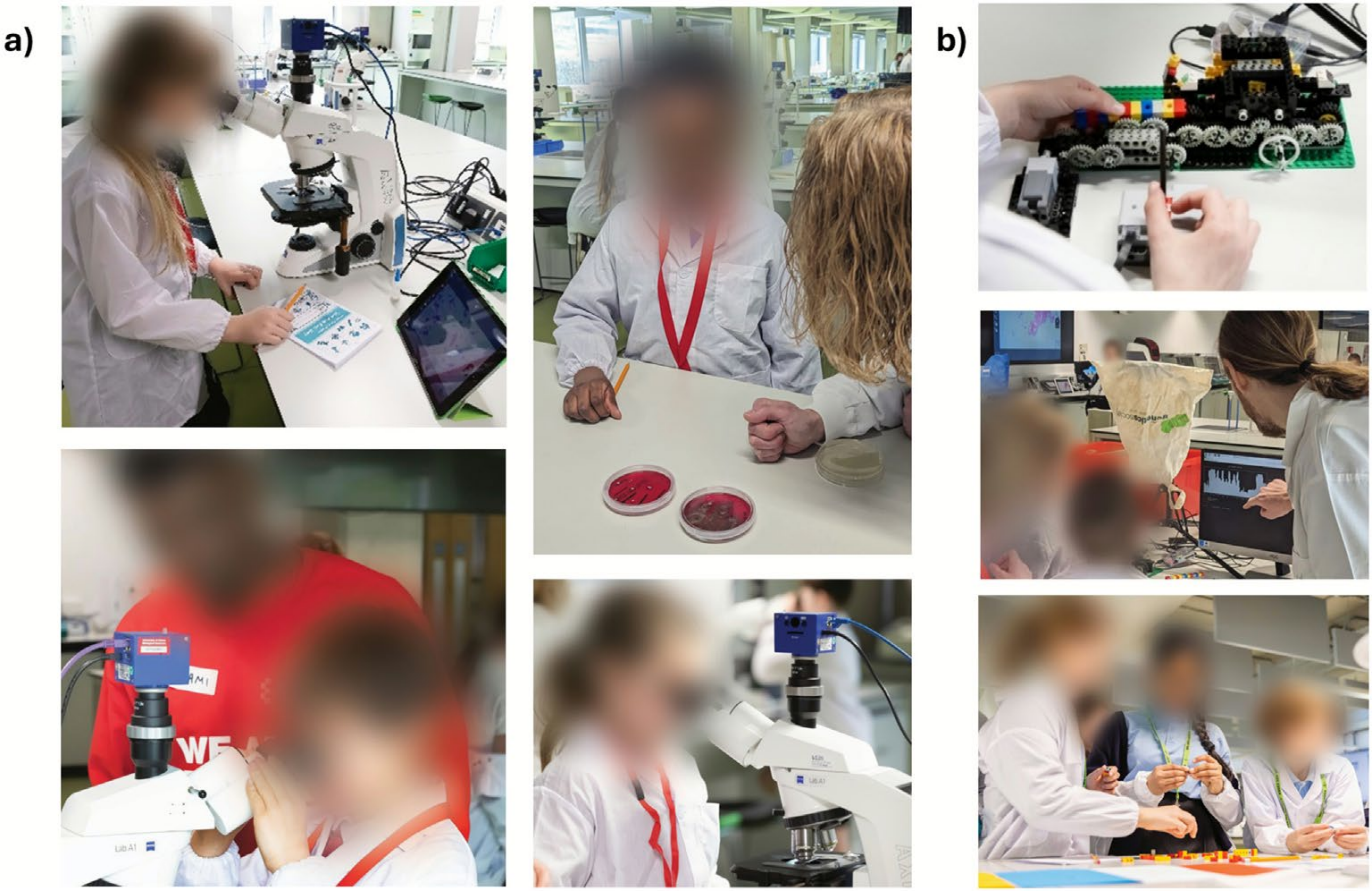
(a) Pupils observing bacteria and fungi under the microscope with the help of staff and Student Ambassadors. Pupils were shown the importance of washing hands for personal hygiene with demonstration plates of a handprint before and after washing by Dr. Aurélie Villedieu. (b) The ‘Legopore’ developed by Dr. Ben Skinner enabled students to create and ‘read’ their own ‘DNA sequences’.

Pupils explored the morphology of different microbes under the microscope (see Figure 3a). For example, they looked at how microbiologists use coloured stains like the Gram stain to help distinguish between Gram‐positive and Gram‐negative bacteria, such as *Bacillus subtilis* and *Escherichia coli* . Pupils also looked at different species of fungi (e.g., *Penicillium* spp.) that had been isolated from school classrooms. They were shown how they grow and reproduce and taught about their significance to human health, such as in the production of antibiotics. Some slides included both yeast cells and bacteria so that pupils could compare the size of different types of microbes. Being able to see yeast cells helped to foster pupils' understanding of microbes that are crucial for food production. Pupils were also shown the importance of washing hands in personal hygiene to remove microbes. This was done with demonstration Columbia Blood Agar plates showing colonies in the shape of a handprint from a staff member, where one hand had been washed and the other one had not been washed (see Figure 3a). Pupils saw more colonies deriving from the unwashed hand. In addition to looking down the microscope and at agar plates, pupils could see the magnified view of their microbes on computer tablets so they could share the images with the wider group.

### 2.6 Virtual Reality (VR) Headsets

Using VR headsets, pupils were able to see scientists at work in the different research facilities at the University of Essex and experience student practical classes in the STEM Teaching Laboratory (see Figure S2). This activity was only included in the 2024 event as we focused pupils' time on activities that involved interaction with staff and each other in 2025.

### 2.7 The ‘Legopore’ Sequencer

Pupils were introduced to the ‘building blocks of life’ (DNA) and a method of identifying microbes through the medium of LEGO (see Figure 3b). Direct DNA sequencing (e.g., by Oxford Nanopore) was demonstrated via a ‘DNA sequencer’ built from LEGO Technic bricks and a Raspberry Pi camera and computer. Four colours of LEGO brick correspond to the four DNA bases; when students pull a lever, a motor drives the LEGO DNA past a camera. The brick colours are converted to DNA base calls in the computer and visualised on screen using a custom Python script (Skinner 2023). Pupils built and ‘read’ their own short DNA sequences, seeing how different nucleotides give different measurements from the sequencer.

### 2.8 Marine Activities and Fish Ecology

**FIGURE 4:**
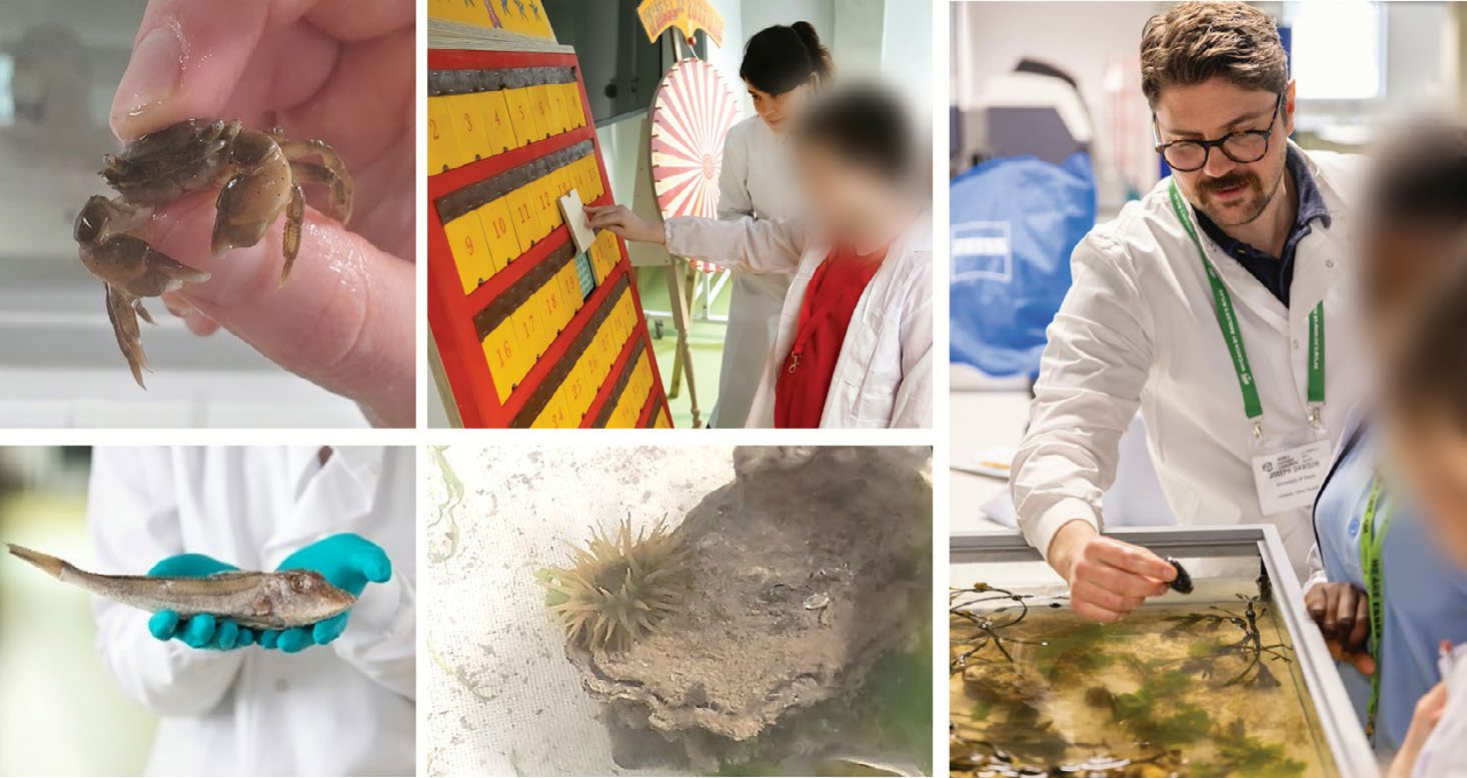
Marine activities included a shallow seawater tank showing local marine species such as anemones and mussels (2024 event), discussing adaptations of a grey gurnard, counting otolith growth rings, and playing the Wheel of Misfortune (to see if your ‘fish’ survived to the beach and to its first birthday) to encourage pupils to think about the importance of our rivers and coastlines as nursery areas and the steps we can take to improve their health and productivity (2025 event).

In 2024, pupils were shown a shallow 10‐L seawater tank with green crabs ( *Carcinus maenas* ), periwinkles ( *Littorina littorea* ), beadlet anemones ( *Actinia equina* ), oysters (*Magallana gigas*) and macroalgae (e.g., *Ulva lactuca, Fucus spiralis*), so they could get close to and learn about their local marine species (see Figure 4). In 2025, pupils looked at a sectioned 6‐year‐old cod ( *Gadus morhua* ) otolith (fish ‘earstone’) under a dissecting microscope and counted the annual growth rings (just like tree‐trunk rings). They were told how these ‘bones’ help us avoid overfishing by allowing scientists to check that humans are not removing too many of the oldest or youngest fish from the sea.

Pupils also took part in the ‘Wheel of Misfortune’ game in 2025 (see Supporting Information document Appendix S1 for details) to learn about the perils faced by larval and juvenile fish and to encourage the pupils to make the link that the fish that they eat may have grown up in their local river, beach or estuary, and for us to brainstorm how we can improve the health of these ‘nursery habitats’ to help more fish survive.

### 2.9 Nature Walk

**FIGURE 5:**
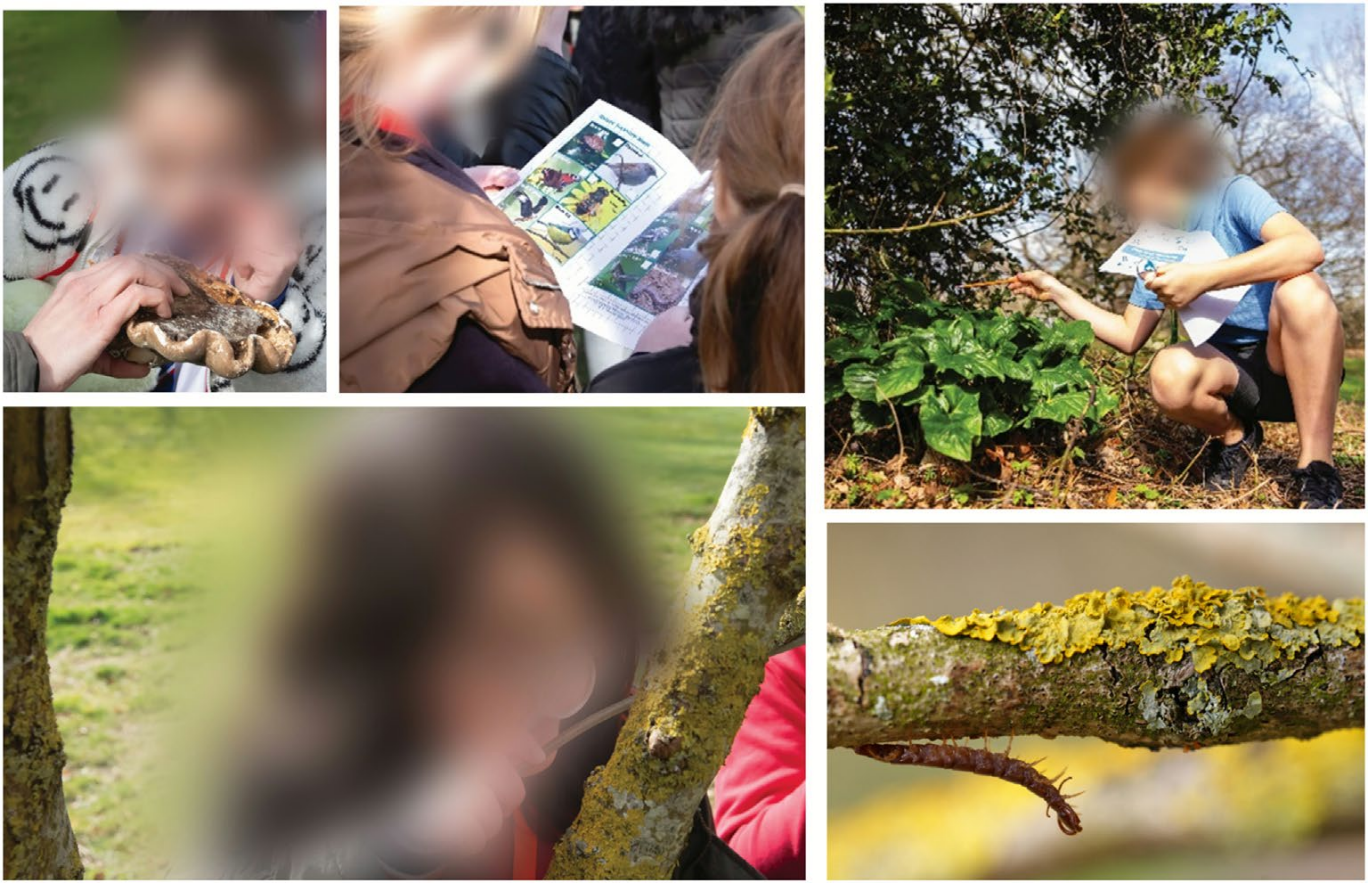
On the ‘nature treasure hunt’, pupils had to spot a range of plants and animals included in their booklets. They were provided with a magnifying glass to help them observe the fine detail and structures of insects, plants, fungi and lichens. Bottom right photo credit: Jay Burke photography.

To introduce local biodiversity representative of the region, pupils took part in a nature walk around the grounds of the University of Essex, framed as a ‘nature treasure hunt’ (see Figure 5). Pupils had to spot a range of plants and animals included in their booklets. They were provided with a magnifying glass to help them observe fine details and structures. The microbial world was made visible to students in the form of fungi and lichens, which staff stopped to point out and discuss regularly and linked back to the microbes pupils had seen, or were going to see, under the microscope (e.g., observing fungi growing on dead wood linked to topics such as nutrient cycling). The nature walk lasted approximately 40 min, with 10 min scheduled on either side of the main activity for walking across campus and washing hands. Upon pupils' return to the room, there was time to recap as a group on all the species that had been spotted and for pupils to ask questions. This also included more general discussion about university life, as well as the different science subjects available to study.

### 2.10 End‐Of‐Event Assembly

#### 2.10.1 Post‐Event Surveys

After the afternoon activity, pupils were given the post‐event survey to complete, which repeated the questions asked at the start of the day, as well as additional questions to evaluate the different elements of their Science Fun Day experience (see Data S1 and S3 in Supporting Information). Teachers were also given a form to submit their feedback on the Day.

#### 2.10.2 Prize‐Giving

To round the day off, one pupil from each class was chosen by their teachers to receive a prize for their enthusiasm—a tardigrade (‘water bear’) plush toy. All pupils received a badge and certificate of ‘scientific excellence’ for attending.

### 2.11 Data Analysis of Pupil Surveys

All analysis was conducted with R version 4.5.0 (R Core Team 2025) in RStudio version 2025.5.0.496 (Posit team 2025) with the tidymodels meta‐package (version 1.3.0; Kuhn and Wickham 2020). Ordinal logistic regression models were built with ordinal (version 2023.12.4.1; Christensen 2023). A Brant test from the gofcat package (version 0.1.2; Ugba 2022) was used to check the proportional odds assumption for each model. Data were visualised with ggplot2 (version 3.5.2; Wickham 2016).

Survey data from 2024 and 2025 were analysed separately because we did not do exactly the same activities in both years. For example, we did not run the VR headset activity in 2025 and, in 2024, the ‘marine activity’ was the marine tank whilst in 2025, this was replaced with the otolith demonstration and ‘Wheel of Misfortune’ game. Ordinal logistic regressions were produced to assess the impact of the Science Fun Days on pupils' responses to each question. In 2024, an interaction term was included to assess whether the effect of attending an event differed between school groups. In addition to the school interaction term, a gender‐identity interaction term was also included in the 2025 models to understand if attitudes towards science differ with gender and whether male‐ and female‐identifying pupils respond to this form of outreach in different ways. Pupils were asked to write down the gender they identify with, not tick a box; therefore, responses were grouped into female and male identities (i.e., ‘girl’ and ‘woman’ as ‘female’ and ‘boy’, ‘man’ and ‘male’ as ‘male’). Pupils who did not answer have been grouped into the ‘unspecified’ gender identity category.

Further analysis was conducted to investigate the influence of the socio‐economic status of the school location on pupils' responses to survey questions, based on the Index of Multiple Deprivation (IMD) decile assigned to each school's postcode (Ministry of Housing, Communities, and Local Government (2018 to 2021) 2019) (see ‘School Demographics’). Schools were allocated to either the ‘high‐deprivation‐location’ IMD group (IMD decile 1–5; located within the 50% most deprived areas in England), or the ‘low‐deprivation‐location’ IMD group (IMD decile 6–10; located within the 50% least deprived areas in England). Ordinal logistic regressions were conducted as described above, with an interaction term for the ‘IMD group’ instead of a term for ‘school’. This analysis was only conducted on 2025 data as only one school was allocated to the ‘low‐deprivation‐location’ group in 2024. Gender identity was not included as it was not the focus of this additional analysis.

## 3 Results

### 3.1 Pupil Surveys

#### 3.1.1 School Demographics

A subset of pupils who had parental consent took part in the surveys. In 2024, 152 and 153 pre‐ and post‐ surveys were completed by pupils from Schools 1, 2 and 3. In 2025, 156 and 154 pre‐ and post‐event surveys were completed by pupils from Schools 3, 4, 5, 6 and 7. In 2025, we asked pupils to report their gender identity. The proportion of female‐ and male‐identifying pupils varied across school groups, ranging between 22.2% to 45.5% female‐identifying and 36.4% to 77.8% male‐identifying. The number of pupils within a school group who did not complete the gender‐identity question ranged from 0.0% to 68.2% in the pre‐event survey and 0.0% to 21.2% in the post‐event survey. This may have been influenced by arrival time at the event (pupils were asked to record their gender identity in one of the final questions) which varied with school. For instance, 68.2% of School 5 pupils, who were last to arrive, did not complete the gender‐identity question in the pre‐event survey; yet all except 18.2% of School 5 pupils responded to this question in the post‐event survey.

The seven schools that completed the survey vary in their location's national rankings across the English Indices of Deprivation 2019 (Ministry of Housing, Communities, and Local Government (2018 to 2021) 2019; see Table 1). The Index of Multiple Deprivation (IMD) combines data from seven different domains such as ‘Employment’, ‘Education and skills’, ‘Living Environment’ to score the overall relative deprivation experienced in different geographic areas known as Lower‐layer Super Output Areas (LSOAs) that is, each neighbourhood of ~1500 residents, across England (Penney 2019). Schools 1, 4, and 7 are located within the 20% most deprived LSOAs in England by 2019 IMD score, whilst schools 2 and 3 rank in the 10% least deprived by this metric. The locations of Schools 1 and 4 rank in the bottom 10% of the ‘Education and skills’ domain in England which measures lack of attainment and skills and the bottom 10% and 20% of ‘Income Deprivation Affecting Children Index’ (IDACI), respectively. Schools 3 and 5 are also located in LSOAs ranked in the bottom 50% for IDACI in England. All schools, except 6 and 7, are located in LSOAs where the quality of the ‘Living Environment’, which encompasses indoor (housing) and outdoor spaces, is ranked in the top 50% in England.

**TABLE 1:** Schools surveyed and their location's decile of deprivation across multiple domains. Data from the English Indices of Deprivation 2019 (Ministry of Housing, Communities, and Local Government (2018 to 2021) 2019). For analysis, schools surveyed in 2025 were placed into two IMD groups: ‘high‐deprivation‐location’ schools (IMD decile 1–5) and ‘low‐deprivation‐location’ schools (IMD decile 6–10).

School	*n* Survey responses^a^		2019 Decile in the UK from 1 (most deprived) to 10 (least deprived)				
			Index of Multiple Deprivation (IMD)	Income Deprivation Affecting Children Index (IDACI)^b^	Employment^b^	Education and skills^b^	Living environment^b^
	Pre	Post					
1	53	52	2	2	3	1	9
2	47	56	10	10	10	10	6
3	52; 18	45; 19	10	5	10	8	9
4	55	52	2	1	2	1	8
5	22	22	4	4	2	2	8
6	35	34	7	6	7	7	3
7	25	25	2	7	2	5	2

^a^ School 3 *n* = 2024; 2025.

^b^ One of seven ‘domains’ contributing to the IMD score.

#### 3.1.2 Attitudes Towards Science & Future Career

**FIGURE 6:**
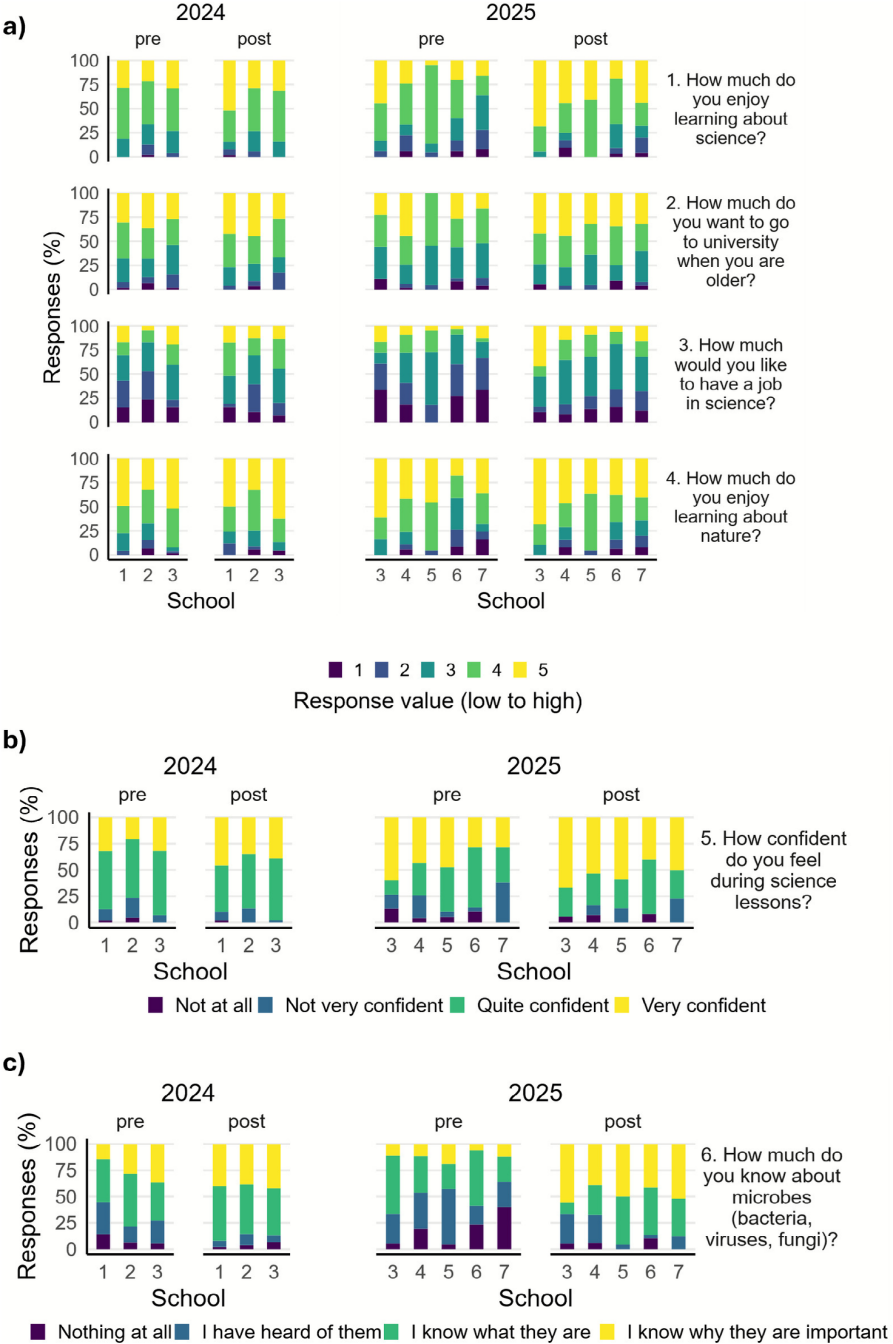
Survey results at the start (pre) and end (post) of the event grouped by School and year of event. Questions related to (a) attitudes towards science and nature. (Scores on a 5‐level Likert scale with 1 being lowest and 5 being the highest.) (b) Confidence in Science lessons. (c) Knowledge of microbes.

Figure 6a shows the response to questions relating to future career aspirations and the pupils' enjoyment of learning about science and nature. Overall, pupils reported greater enjoyment of learning about science (question 1) after taking part in the Science Fun Day in 2024 (*p* ≤ 0.05, OR 2.07, 95% CI 1.00–4.32; 107.3% increase in odds). The percentage of pupils who answered positively (a score of 4/5 or 5/5) in 2024 increased from 73.7% to 80.4% by the end of the day and from 64.9% to 78.0% in 2025; however, there was no significant impact of the event in 2025 on responses to this question (*p* = 0.06, OR 4.28, 95% CI 0.96–20.07).

When asked to score how much they want to go to university when they are older (question 2), 63.1% and 61.4% of pupils responded positively in the pre‐event surveys of 2024 and 2025, which increased post‐event to 72.6% and 71.3%, respectively, but overall, this was not significant (2024: *p* = 0.18, OR 1.60, 95% CI 0.80–3.19, 2025: *p* = 0.22, OR 2.46, 95% CI 0.58–10.52).

Over both years, the Science Fun Day led to a significant increase in pupils stating they would like to have a job in science (2024: *p* < 0.05, OR 2.24, 95% CI 1.11–4.53; 2025: *p* < 0.01, OR 7.54, 95% CI 1.72–33.30). The percentage of pupils who positively rated their desire to have a job in science increased from 29.6% to 41.9% in 2024 and from 21.8% to 32.9% in 2025. In 2024, there were no differences in responses between schools; however, in 2025, the impact of attending the event on how much pupils want a job in science was significantly lower for School 5 than for School 3 (*p* < 0.05, OR 0.12, 95% CI 0.02–0.69). Whilst a similar proportion of pupils in these schools answered positively for this question before the event (School 3: 27.8%, School 5: 27.2%), this response nearly doubled for School 3 pupils to 52.6% post‐event compared to School 5 pupils' ratings which increased to 31.8%.

There was no overall significant change in pupils' self‐reported enjoyment of learning about nature (question 4) at the end of the Science Fun Days (2024: *p* = 0.91, OR 0.96, 95% CI 0.45–2.03, 2025: *p* = 0.79, OR 0.80, 95% CI 0.16–4.12). Over 70% of pupils gave a positive response (4/5 or 5/5) in 2024 (pre: 79.6%, post: 78.6%) and 2025 (pre: 70.8%, post: 74.7%). However, responses varied between schools in 2025 as School 6 and School 7 scored their responses to this question significantly lower overall than School 3 (*p* < 0.001, OR 0.11, 95% CI 0.03–0.33; *p* < 0.01, OR 0.19, 95% CI 0.05–0.63). School 3 pupils who attended in 2025 exhibited the greatest enjoyment of learning about nature: 83.3% and 89.5% of these 18 pupils gave a positive response in the pre‐ and post‐event surveys, respectively. By contrast, 41.1% of School 6 pupils (*n* = 34) responded positively in the pre‐event survey which increased to 65.6% of pupils in the post‐event survey. Male‐identifying pupils who attended in 2025 rated their enjoyment of learning about nature significantly lower overall than their female‐identifying peers (*p* < 0.001, OR 0.23, 95% CI 0.11–0.48; see Figure S6).

Pupils were asked to rate their agreement with a further set of statements in 2025 surveys (questions 7 to 14 shown in Figure S4 and all model results reported in Table S1). Attendance of a Science Fun Day significantly increased pupils' ratings in response to ‘I like to do science activities’ (*p* < 0.05, OR 7.26, 95% CI 1.60–35.07), ‘I enjoy watching science and nature shows on TV’ (*p* < 0.05, OR 5.71, 95% CI 1.44–23.65), ‘I am good at understanding some science topics’ (*p* < 0.01, OR 7.43, 95% CI 1.79–31.74) and ‘I am good at explaining science’ (*p* < 0.001, OR 11.79, 95% CI 3.00–48.09). Male‐identifying pupils scored their responses to the latter two statements (questions 12 and 13) significantly higher overall than female‐identifying pupils (question 12: *p* < 0.05, OR 2.52, 95% CI 1.24–5.11; question 13: p < 0.05, OR 2.44, 95% CI 1.21–4.95; see Figure S1).

#### 3.1.3 Confidence in Science Lessons

Figure 6b shows how confident pupils report feeling during science lessons (question 5). Between 12 and 26 pupils either did not provide an answer or responded ‘don't know’ to this question for each set of pre‐ and post‐event survey responses per year. In 2024, over half of pupils felt ‘quite confident’ during science lessons in the pre‐ (57.5%) and post‐ (51.1%) event surveys. The proportion of pupils who felt ‘very confident’ increased from 28.4% to 39.7%; however, there was no significant difference in overall scores before and after attendance (*p* = 0.18, OR 1.72, 95% CI 0.18–0.78). Similar results were obtained in 2025 and, although attending an event had no significant impact on pupil scores (*p* = 0.15, OR 3.52, 95% CI 0.63–19.91), over half of pupils (53.1%) said they felt ‘very confident’ during science lessons by the end of the day. In 2025, male‐identifying pupils expressed significantly greater confidence in science lessons overall than female‐identifying pupils (*p* < 0.05, OR 2.66, 95% CI 1.16–6.15; see Figure S7). Although no statistically significant effect on confidence due to attending was observed, the proportion of female‐identifying pupils who reported feeling ‘very confident’ in science lessons increased from 32.3% (pre‐event) to 50.0% (post‐event). This result is similar to the proportion of male‐identifying pupils who answered they felt ‘very confident’ in the pre‐event (46.5%) and post‐event (53%) surveys.

#### 3.1.4 Understanding the Importance of Microbes

Pupils were asked to indicate how much they know about microbes in question 6 (Figure 6c). The pre‐event surveys in 2024 suggested good pre‐existing self‐reported knowledge of microbes, because 42.2% of pupils said they know what microbes are whilst a further 26.5% of pupils indicated that they know one or more reasons why microbes are important (68.7% combined). Attending the 2024 Science Fun Day resulted in a 400% increase in the odds of pupils ranking their response to this question one level higher (*p* < 0.001, OR 5.00, 95% CI 2.38–10.63). By the end of the event, 88.0% of pupils reported they at least knew what microbes were. Pupils from School 2 and School 3 reported significantly greater knowledge of microbes than School 1 overall (School 2: *p* < 0.05, OR 2.64, 95% CI 1.25–5.65; School 3: *p* < 0.01, OR 2.98, 95% CI 1.42–6.33) and attending the event had a lower impact on their scores than School 1 (School 2: *p* < 0.05, OR 0.31, 95% CI 0.11–0.88; School 3: *p* < 0.05, OR 0.32, 95% CI 0.11–0.91). At the start of the day, 55.1% of School 1 pupils knew what microbes were or knew one or more reasons why they are important. After the event, 92.0% of pupils in this group reported this level of knowledge.

In 2025, the proportion of pupils who said they know what microbes are or why they are important increased from 49.3% in the pre‐event survey to 79.1% at the end of the day; however, this was not a statistically significant impact (*p* = 0.06, OR 4.17, 95% CI 0.95–18.90). School 7 exhibited significantly lower self‐reported knowledge of microbes overall than School 3 (*p* < 0.05, OR 0.30, 95% CI 0.10–0.92).

#### 3.1.5 Evaluation of the Science Fun Day Event

Additional questions were asked in the post‐survey for evaluation purposes (see Figures S3, S5 and S9). Pupils were asked to rate their enjoyment of the Science Fun Day on a scale of 1 to 5 with 1 being the lowest and 5 being the highest (Question 15). Of the 151 pupils who responded to this question in 2024, 68.2% rated their enjoyment as 5/5 followed by 23.8% of pupils who scored the event as 4/5. Similarly, in 2025, 70.0% and 19.3% of 140 pupils rated their enjoyment as 5/5 and 4/5, respectively. Question 16 asked pupils whether they had ‘learnt something new’ during the event. In 2024, 87.2% responded ‘yes’ and 3.4% ‘no’ whilst 9.4% ticked ‘don't know’. The 2025 event received 92.4% ‘yes’ responses and 7.6% ‘no’ responses, overall. One hundred percent of pupils from three of the five schools that took part in 2025 (schools 3, 5, and 7) responded ‘yes’ they had learnt something new during the Science Fun Day.

We asked pupils to choose the activity they enjoyed the most (Question 17). The majority of pupils (57.9% in 2024; 72.9% in 2025) chose the ‘STEM Lab’ as the activity that they enjoyed the most, followed by the ‘outdoor activities’ that is, nature walk (28.6% in 2024; 16.7% in 2025). School 2 was the only group where more pupils enjoyed the nature walk the most (52.2%) compared to the STEM Lab activities (41.3%). The assembly at the start of the event was considered the least enjoyable part of the day by 48.9% (2024) and 55.3% (2025) of pupils.

Question 19 asked pupils to answer what their favourite part of the day was, in free‐form writing. Responses varied (see Box 1) and reflect the variety of short activities that pupils were given the chance to participate in.

BOX 1 **Pupil feedback: 19. What was your favorite thing about the Science Fun Day?**

- ‘Overall I liked everything in the Science Fun Day because everything was unique!’
- ‘We had to learn about microorganism and how they affect plant humans and animals’
- ‘My favorite thing was going on a walk and exploring nature’
- ‘The indoor activities and when we used the microscopes and saw the marine tank’
- ‘My favorite thing about the science fun day was learning about all the new things I didn't know before’
- ‘I liked the VR headset it was very surreal and interesting’
- ‘The welcome. The Science activities – taking samples.’
- ‘Definitely the experiment with the testing bacteria! I thought it was very interesting.’

#### 3.1.6 Influence of Index of Multiple Deprivation (IMD)

All questions were further analysed to investigate the potential impact of socio‐economic deprivation on pupils' answers in 2025 (see ‘Data Analysis of Pupil Surveys’ in ‘Experimental Procedures’). Schools 4, 5, and 7 were assigned to the ‘high‐deprivation‐location’ group (IMD decile 1–5) and schools 3 and 6 were assigned to the ‘low‐deprivation‐location’ group (IMD decile 6–10). Most questions did not reveal differences in responses between pupils attending schools in areas of high deprivation compared to low deprivation (for all model results, see Table S2 and Figures S10–S13 in Supporting Information document Appendix S1). However, unequal sample size between groups may influence these results (102 pre‐ and 99 post‐event survey responses from the high‐deprivation‐location group; 53 pre‐ and 53 post‐event survey responses from the low‐deprivation‐location group). Below, the few questions that elicited a significantly different response between groups are mentioned.

In 2025, pupils in the ‘high‐deprivation‐location’ schools rated their desire to have a job in science (question three) significantly higher overall than pupils from the low‐deprivation‐location schools (*p* < 0.05, OR 1.98, 95% CI 1.07–3.66). There was no difference between deprivation groups in the impact of attending the event on their response to this question (*p* = 0.16, OR 0.54, 95% CI 0.23–1.28). Further, when asked if they would like to do more of these kinds of activities in class (question 23), pupils from schools in the high‐deprivation‐location group responded with significantly higher scores than pupils in the low‐deprivation‐location group (*p* < 0.05, OR 2.07, 95% CI 1.05–4.12).

### 3.2 Teacher Feedback

For feedback, teachers were asked to suggest ‘what three things were positive or most useful from your visit?’. Many responses refer to the chance for the children to visit a university campus and the opportunity it provided for pupils to ‘better understand ongoing education’ (see Box 2). Teachers were also asked to reflect on what impact the visit had on their students (see Box 3). As well as ‘increased confidence of new experiences*’* teachers mention that it gave pupils ‘the opportunity to see science in a fun environment*’* and that pupils ‘were talking about it in school the next day*’*.

BOX 2 **Teacher feedback: What 3 things were positive or most useful to you from your visit?**

- ‘Having the opportunity to come out of school to somewhere where Science is done and to meet people who actually work in the field of Science was great’
- ‘Children having a tour of university and better understanding ongoing education’
- ‘Interesting hands‐on activities’
- ‘Meeting real scientists’
- ‘…the children all got to spend some time in a proper Science lab – including wearing lab coats and gloves – and to take part in an activity where they got to use scientific equipment was a brilliant opportunity…’

BOX 3 **Teacher feedback: What impact do you think your campus visit had on your students?**

- ‘Increased confidence of new experiences prior to secondary school’
- ‘The children seemed really engaged by the activities and lots were asking questions about how to get into university and become a scientist. They all seemed to have lots of fun.’
- ‘The children were all really excited about the trip and were talking about it in school the next day. They loved the certificates and badges – many of them have been proudly wearing their badges all week on their school jumpers. They loved the fact that they got to go in a Science lab and that they got to use magnifying glasses to look at things from nature.’
- ‘I think it had a really positive impact on our children as they had the opportunity to see Science in a fun environment.’
- ‘Lots said they would like to go to university in future. Excited about Science.’

## 4 Discussion

‘Science Fun Day’ outreach events were organised for primary school children aged 9–11 years to enhance their knowledge and understanding of microbes whilst promoting enjoyment of learning about science and nature more broadly. Across 2 weeks of events in successive years, > 450 primary school pupils from eight local schools attended an event and over 300 pupils from seven of these schools took part in our pupil survey.

### 4.1 Benefits of Science Fun Days (Qualitative and Quantitative [Longitudinal Study Notwithstanding]) Are Very Clear for Both Children and Teachers (and Potentially Families)

Across both years, we observed a statistically significant increase in pupils' desire to pursue a job in science compared to the pre‐event scores. This result supports the importance of primary science outreach events for motivating pupils to consider a science‐related career. This is especially noteworthy as science‐related career aspirations typically form before pupils reach secondary education (Tai et al. 2006; Archer et al. 2015; Salvadó et al. 2021). Similar out‐of‐school events such as science festivals, science fairs and interactive workshops captivate pupils with exciting novel activities that encourage positive attitudes towards science, and provide opportunities for engagement, enhancing their ‘science capital’ (Archer et al. 2015) and interest in science‐related careers (The Royal Society 2014). In particular, the ‘growing microbes’ activity included in the Science Fun Days, is an example of an interactive workshop that emphasises student‐led inquiry—students decided which surface to grow bacteria from—whilst being guided by professional staff members. The exploratory and non‐competitive style of interactive workshops enables pupils to engage in science with less anxiety and pressure, making the experience more enjoyable and likely to foster confidence in their ability (Muñoz‐Losa and Corbacho‐Cuello 2025).

Another core feature of the Science Fun Days was the interaction pupils had with real scientists, many of whom identify as female, and university students studying for science degrees, which teachers and pupils mentioned in their feedback. For example, teachers positively highlighted ‘meeting real scientists*’*, and ‘having the opportunity … to meet people who actually work in the field of Science was great*’*. Pupil engagement with STEM role models with whom they identify is crucial for developing their ‘science identity’—seeing oneself as someone ‘who does science’ (DeWitt et al. 2016; Salvadó et al. 2021)—and for breaking down stereotypes (Salvadó et al. 2021). Building these connections is especially key for encouraging pupils from low‐income backgrounds, who are less likely to have existing personal connections with science‐related role models (Archer et al. 2012), to choose science subjects beyond compulsory education (Korpershoek et al. 2013). Our analysis found that pupils from schools located in the top 50% most deprived locations in England responded with an overall greater desire to have a job in science and enthusiasm to do more of the activities in class than their peers from schools located in less deprived areas. Whilst we are unable to determine what could be driving this response, it is positive to report an eagerness for STEM careers and activities in pupils from schools located in areas of high deprivation.

### 4.2 Importance of Contextualising Microbiology as Part of Nature and How Microbes Impact Human Lives

During both years of events, a greater proportion of pupils said, at the end of the day, that they know one or more reasons why microbes are important. However, while in 2024 there was a significant overall increase in self‐reported knowledge of microbes, in 2025 there was no significant difference. Throughout the Science Fun Days, microbes were positively linked to everyday concepts such as health, food and the environment, and pupils were able to observe real microbes such as yeast cells, *Penicillium* spp. and examples of Gram‐positive ( *Bacillus subtilis* ) and Gram‐negative (*Escherichia coli*) bacteria under the microscope (McGenity et al. 2020).

### 4.3 Gender Identity—Differences in Self‐Reported Confidence and Ability

For 14 out of 19 questions where differences between gender identity were investigated, male‐ and female‐identifying pupils did not report significantly different responses. In the other five questions, differences included that male‐identifying pupils self‐reported significantly greater overall confidence in science lessons and agreement with the statements ‘I am good at understanding some science topics’ and ‘I am good at explaining science’ than their female‐identifying peers across both pre‐ and post‐event surveys. In the UK, female pupils consistently outperform male pupils in science at the national level (Department for Education 2025), suggesting that the relatively lower confidence expressed by female‐identifying pupils may be linked to the persistence of gender‐related stereotypes. A greater proportion of female‐identifying pupils said they felt ‘very confident’ in science lessons in the post‐event surveys (pre‐event: 32.3%, post‐event: 50.0%) and this was similar to the proportion of male‐identifying pupils who gave this response before (46.5%) and after (53.0%) the event, which, although not statistically significant, is a promising trend. The Science Fun Day was led by a female researcher who was assisted by many female staff and Student Ambassadors, which may have contributed positively to the experience of female‐identifying pupils. Providing pupils with real examples of women working in STEM is important for promoting less stereotypical conceptions of STEM professionals (So et al. 2022). Gender stereotypes of intellectual ability can arise in children as young as 6 years old (Bian et al. 2017); therefore early intervention through experiences during primary school is important.

### 4.4 Laboratory Activities Were (Mostly) Enjoyed More Than Nature Activities

Pupils were highly enthusiastic about taking part in scientific activities in the University's STEM Teaching Laboratory. The opportunity to use technical equipment such as microscopes and pipettes was novel and exciting to students who haven't previously had the chance to experience this. There is broad psychological consensus that emotional events are remembered more vividly and for longer (Tyng et al. 2017), therefore encouraging a sense of excitement in pupils may boost their learning and retention of new information. However, it is important that students understand the context of the information—a criticism of primary practical science in England is the use of fun hands‐on activities which do not link back to wider learning (Bianchi et al. 2021). We aimed to integrate microbes into familiar topics (habitats, food and health), facilitated by scientists with expert knowledge of microbiology, to ensure that pupils engaged with ‘hands‐on’ activities with their ‘minds‐on’ too. Use of ‘hands‐on’ activities involving equipment that could be visually demonstrated to pupils may also have benefitted pupils' learning, because physical tasks that rely less on language proficiency are more accessible (Tang et al. 2022). This is crucial for pupils to remain engaged, especially when microbiology literacy at primary school age is typically low (Timmis 2023c).

On the nature walk, pupils were also encouraged to interact with objects and physical phenomena. For example, they used magnifying glasses to observe lichens and insects on dead wood and leaves they picked up. Previous case studies have generally reported positive impacts of practical ‘hands‐on’ experiences of biodiversity during childhood on biodiversity awareness later in life (Beery and Jørgensen 2018). In addition, such exposure to nature during childhood has been shown to increase nature relatedness in later life, whilst deprivation of these experiences may lead to low sensory registration in adulthood (Li et al. 2022). Consequently, providing pupils with memorable and practical experiences of local nature may have long‐lasting social and physical benefits throughout their lives.

Male‐identifying pupils reported lower enjoyment of learning about nature and a lower desire to understand how nature works compared to their female‐identifying peers. These findings may reflect higher levels of nature connectedness that have been reported by female primary school pupils compared to their male counterparts (Keith et al. 2021; Price et al. 2022). However, no gender differences were identified in how much pupils enjoy reading, watching or talking about nature.

### 4.5 Changes Made Based on 2024 Feedback

Whilst the 2024 Science Fun Days were received positively overall (see ‘Evaluation of the Science Fun Day event’ in the Results section), areas for improvement included the quality of pupils' time spent at each activity, staff numbers on the nature walks and the amount of paperwork for teachers. In 2025, we ensured two members of staff were assigned to each activity, which meant that one staff member could focus on giving instructions on how to use the equipment whilst another could provide scientific context and answer questions, leading to, anecdotally, more effective engagement with pupils. We also did not include the VR headset activity in 2025 to focus pupils' time and attention on the STEM Laboratory activities with interactive scientific content that facilitated greater collaboration amongst pupils, rather than being observation‐only. We recruited two additional staff or PhD student volunteers per nature walk to increase the presence of university representatives (including Student Ambassadors) during the activity to 8 per group of ~60 pupils (1 per ~8 pupils). In 2025, all paperwork was delivered to teachers in one instance. To further decrease the effort required by teachers, hard copies of permission forms (survey consent, photographic permissions) were provided.

### 4.6 Challenges

A major challenge was ensuring that enough time was available throughout the day for pupils to move between activities and locations without delays to avoid restlessness. Clear communication with teachers, university staff and Student Ambassadors was essential to keeping as close to schedule as possible whilst allowing for adequate breaks. We recommend advance booking of large‐capacity rooms to ensure availability for an entire day to minimise the time spent moving between rooms and to ensure there is indoor space available for alternatives to the outdoor activity in bad weather.

Engagement with the classroom bioaerosol sampling activity once back at school was low as only four out of the 10 sampling packs were returned to the University. There may be many different reasons for this low engagement, for example, time constraints or teachers potentially feeling less confident in delivering the material; however, it highlights the challenge of continuing engagement outside of the university setting.

### 4.7 Survey Design Limitations

Changes in pupils' attitudes and perspectives were evaluated with a self‐reporting survey that used Likert Scale responses. Although results revealed significant immediate positive impacts of the event on pupils, future work could involve repeating the survey with the same pupils 1 month or more following the event to assess whether the event has had a lasting impact on attitudes and perceptions. Further, the inclusion of knowledge‐testing questions (rather than self‐reported knowledge) would be useful to reduce potential bias in responses.

Pupils were asked to include their school class group on the survey form. However, most pupils did not do this, so we were unable to account for possible differences in survey responses between class groups within the same school taught by different teaching staff.

## 5 Conclusion

Primary school visits to local universities can enhance positive attitudes towards science and encourage the scientific career aspirations of pupils. A key aspect here was to engage pupils with exciting ‘hands‐on’ activities in a non‐competitive environment that were led by real research scientists. For example, pupils especially enjoyed the opportunity to observe bacteria and fungi under the microscope and grow bacteria from samples they had collected themselves. This experience also provided pupils with a snapshot of university life, opening up new opportunities that they may not have previously thought were available to them, and aided by interaction with student role models (Student Ambassadors), to inspire future learning. Throughout each ‘Science Fun Day’ event, we fostered an enthusiasm for microbes by highlighting their importance in our everyday lives and enhanced the pupils' access to nature.

## Author Contributions

**Terry J. McGenity:** conceptualization, investigation, funding acquisition, writing – original draft, methodology, writing – review and editing, supervision, project administration, resources, formal analysis, visualization. **Elizabeth J. Archer:** conceptualization, investigation, funding acquisition, writing – original draft, methodology, visualization, writing – review and editing, formal analysis, data curation, project administration, resources. **Robert M. W. Ferguson:** conceptualization, investigation, funding acquisition, writing – original draft, methodology, writing – review and editing, supervision, project administration, resources. **Dave R. Clark:** conceptualization, investigation, funding acquisition, writing – original draft, methodology, visualization, writing – review and editing, supervision, formal analysis, resources. **Corinne B. Whitby:** conceptualization, investigation, funding acquisition, writing – original draft, methodology, writing – review and editing, supervision, resources. **Olivia S. Solanke:** conceptualization, investigation, funding acquisition, writing – original draft, methodology, project administration, writing – review and editing, resources. **Anna M. Sturrock:** funding acquisition, writing – original draft, methodology, writing – review and editing, resources. **Benjamin M. Skinner:** writing – original draft, methodology, writing – review and editing, resources. **Drew K. Henderson:** writing – original draft, methodology, writing – review and editing, resources. **Rebekah Boreham:** writing – original draft, methodology, writing – review and editing, resources.

## Funding

This work was supported by the European Union, 101056883; UK Research and Innovation, 10040524, MR/V023578/1; Swiss State Secretariat for Education, Research and Innovation, 22.00324; Australian National Health & Medical Research Council, APP2017786, APP2008813; Suffolk and North East Essex Integrated Care System, MOU – Project 2; Microbiology Society, GA004526; Suffolk and North East Essex Integrated Care Board; Centre for Healthcare Science University of Essex (funded by NHS England); Academy of Medical Sciences, SBF007\100130.

## Ethics Statement

Ethical approval was obtained from the University of Essex to conduct pupil surveys (application reference: ETH2324‐0242 and ETH2224‐0118). Written permission to include anonymous feedback quotes from teachers has been obtained.

## Conflicts of Interest

The authors declare no conflicts of interest.

## Supporting information


**Data S1:** mbt270279‐sup‐0001‐DataS1.pdf.

**Data S2:** mbt270279‐sup‐0002‐DataS2.pdf.

**Data S3:** mbt270279‐sup‐0003‐DataS3.pdf.

**Data S4:** mbt270279‐sup‐0004‐DataS4.pdf.

**Data S5:** mbt270279‐sup‐0005‐DataS5.pdf.

**Data S6:** mbt270279‐sup‐0006‐DataS6.pdf.

**Data S7:** mbt270279‐sup‐0007‐DataS7.csv.

**Data S8:** mbt270279‐sup‐0008‐DataS8.csv.

**Appendix S1:** mbt270279‐sup‐0009‐AppendixS1.docx.

## Data Availability

The anonymised data that supports the findings of this study is available in the Supporting Information of this article (Data S7 and S8).
