# Supplementary material for: ‘Science Fun Days’: Opportunities for Connecting Primary School Pupils With Nature and Microbiology
Source: Microb Biotechnol. 2025 Dec 10;18(12):e70279. doi: 10.1111/1751-7915.70279 (PMC12696025; doi:10.1111/1751-7915.70279)
Supplement: Supplementary file 2 — Data S2: mbt270279‐sup‐0002‐DataS2.pdf. [file MBT2-18-e70279-s001.pdf]

University of Essex

Science Fun Day pupil survey – before

My school: ……………………………………………………………………

My class: ……………………………………………………………………..

Hello! We are a team of scientists at the University of Essex, and we want to know what you think about studying science. Please help us by answering some questions at the start and end of your Science Fun Day visit to the University. Each survey should take about 10 minutes to complete. If you have any questions about the survey, please ask.

Your answers will be private and kept safe with the team of scientists at the University. We will not share your individual answers but our results for your class may be included in reports, case studies or scientific publications to help us understand and communicate the impact of events like the Science Fun Day so that hopefully more children can visit the University in future.

You can stop completing the survey at any point or skip any question without needing to say why and won't be told off for this. You won't get into trouble if you don't want to complete the survey, it is your choice whether to take part.

You must not write your name on your survey, so we don't know who each set of answers belong to.

The way we look after your information is ruled by UK law. Under UK law, we need to have a good reason for handling your information. In this case, the reason is permission ('consent') from you and your parent/guardian.

Have you read the information above and are you happy to answer this survey? Please tick the box under your answer:

Yes ☐ No ☐

University of Essex

1. On a scale of 1-5, how much do you enjoy learning about science, where 1 is “not at all” and 5 is “really enjoy”?
   (Please circle your answer.)

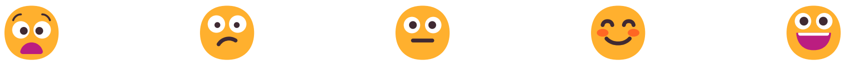

1 2 3 4 5

2. On a scale of 1-5, how much do you want to go to university when you are older, where 1 is “not at all” and 5 is “I definitely want to go”?
   (Please circle your answer.)

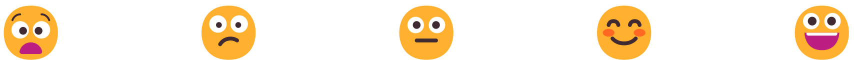

1 2 3 4 5

3. On a scale of 1-5, how much would you like to have a job in science, where 1 is “not at all” and 5 is “I definitely want a job in science”?
   (Please circle your answer)

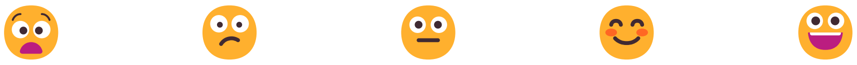

1 2 3 4 5

University of Essex

4. How confident do you feel during science lessons?
   (Please tick the box under the answer you agree with.)

Not at all confident	Not very confident	Quite confident	Very confident	Don't know
☐	☐	☐	☐	☐

5. On a scale of 1 to 5, how interesting do you find learning about science, where 1 is “not interesting at all” and 5 is “very interesting”?
   (Please circle your answer.)

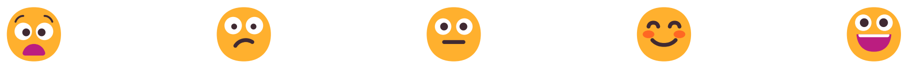

1 2 3 4 5

6. How much do you know about microbes (bacteria, viruses, fungi)?
   (Please tick **all the boxes** that you agree with.)

Nothing at all. I hadn’t heard of them until now.	I know they exist but don’t know what they are.	I know what they are	I know one or more reasons why they are important
☐	☐	☐	☐

University of Essex

7. On a scale of 1 to 5, how much do you enjoy learning about nature, where 1 is “not at all” and 5 is “really enjoy”? (Please circle your answer.)

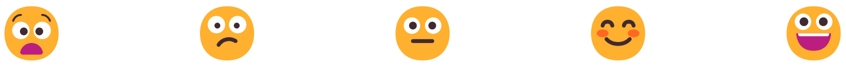

1 2 3 4 5

Thank you from our scientists at the University of Essex.
