# Supplementary material for: ‘Science Fun Days’: Opportunities for Connecting Primary School Pupils With Nature and Microbiology
Source: Microb Biotechnol. 2025 Dec 10;18(12):e70279. doi: 10.1111/1751-7915.70279 (PMC12696025; doi:10.1111/1751-7915.70279)
Supplement: Supplementary file 4 — Data S4: mbt270279‐sup‐0004‐DataS4.pdf. [file MBT2-18-e70279-s008.pdf]

STUDENT QUESTIONNAIRE on AIR QUALITY - Perception and behaviors

(pre-event – Science Fun Day)

School: _____________________________ Class:__________________________

This study has been explained to me by an adult, and I have asked all the questions I want. I am happy to take part in this study and understand I don't have to if I don't want to. **(If 'yes', please tick the box)** ☐

Please read each question carefully and tick the box that best applies to you.

**Questions**	**not at all**	**A little**	**undecided**	**yes**	**yes totally!**
1. I like to do science activities	☐	☐	☐	☐	☐
2. I enjoy learning about science	☐	☐	☐	☐	☐
3. I enjoy learning about nature	☐	☐	☐	☐	☐
4. I want to understand how nature works	☐	☐	☐	☐	☐
5. I enjoy **reading** about science and nature	☐	☐	☐	☐	☐
6. I enjoy **talking** about science and nature	☐	☐	☐	☐	☐
7. I enjoy **watching** science and nature shows on TV	☐	☐	☐	☐	☐
8. I am good at understanding some science topics	☐	☐	☐	☐	☐
9. I am good at explaining science	☐	☐	☐	☐	☐
10. I am good at following instructions for scientific activities	☐	☐	☐	☐	☐
11. I am good at explaining how to do science activities to others	☐	☐	☐	☐	☐
12. I feel confident during science lessons	☐	☐	☐	☐	☐

13. How much do you know about microbes (bacteria, viruses, fungi)?

(Please tick **all the boxes** that you agree with.)

Nothing at all. I hadn't heard of them until now	I know they exist but don't know what they are	I know what they are	I know one or more reasons why they are important
☐	☐	☐	☐

STUDENT QUESTIONNAIRE on AIR QUALITY - Perception and behaviors

(pre-event – Science Fun Day)

**Questions**<br><br>On a scale of 1-5...	1	2	3	4	5
14. How much do you want to go to university when you are older, where 1 is “not at all” and 5 is “I definitely want to go”?	☐	☐	☐	☐	☐
15. How much would you like to have a job in science, where 1 is “not at all” and 5 is “I definitely want a job in science”?	☐	☐	☐	☐	☐

**How would you rate the air quality in each of the following places?**	**bad**	**reasonable**	**good**	**I don't know**
16. Local area around my home	☐	☐	☐	☐
17. Inside my home	☐	☐	☐	☐
18. My Classroom	☐	☐	☐	☐

**Please read each the following statements carefully and choose between true, false or don’t know**	True ✓	False X	Don't know
19. Indoor air quality affects my health	☐	☐	☐
20. Indoor air quality depends on outdoor air pollution	☐	☐	☐
21. Opening a window changes the air quality inside	☐	☐	☐
22. We should use candles at home	☐	☐	☐
23. Fireplaces can release air pollution into the home	☐	☐	☐
24. Cooking can release air pollution into the home	☐	☐	☐
25. Children are a source of air pollution in the classroom	☐	☐	☐
26. How I travel to school affects the air pollution I breathe	☐	☐	☐

STUDENT QUESTIONNAIRE on AIR QUALITY - Perception and behaviors

(pre-event – Science Fun Day)

Please indicate how often you do the following activities?	Always	Often	Sometimes	Rarely	Never	
27. I keep my bedroom tidy, so it is easy to clean	☐	☐	☐	☐	☐	
28. I put my hand in front of my mouth when sneezing or coughing	☐	☐	☐	☐	☐	
29. I open the windows of my bedroom when air is stuffy	☐	☐	☐	☐	☐	☐ not possible

30. Please write down, what are the main SOURCES of AIR POLLUTION in each of the following locations? If you don't know, leave it empty.

- In the local area around my home:

  ____

- Inside my home:

  ____

- In the classroom:

  ____

31. Please write down, what are the main AIR POLLUTANTS in each of the following locations? If you don't know, leave it empty.

- In the local area around my home:

  ____

- Inside my home:

  ____

- In the classroom:

  ____

32. **Optional**: Do you have questions or comments to the research team?

____

____

____

____

33. How old are you? ______ years

34. What gender do you identify with? ______________

Thank you!!!
