# Supplementary material for: ‘Science Fun Days’: Opportunities for Connecting Primary School Pupils With Nature and Microbiology
Source: Microb Biotechnol. 2025 Dec 10;18(12):e70279. doi: 10.1111/1751-7915.70279 (PMC12696025; doi:10.1111/1751-7915.70279)
Supplement: Supplementary file 5 — Data S5: mbt270279‐sup‐0005‐DataS5.pdf. [file MBT2-18-e70279-s009.pdf]

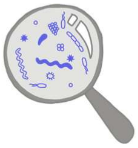

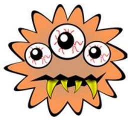

Measure the air quality in your school

Part one: taking the sample

You have 3 air samplers (dust cloths attached to paper) just like the ones we used in the workshop. They are attached to paper so that you can pick them up without touching the cloth itself which would contaminate the sample. The cloth is statically charged, so it will collect dust from the air enabling us to measure the microorganisms in the air.

Unfold your air samplers and place them in your classroom where they won't be disturbed for one month. For example, on top of a shelf or windowsill is best. If you think they will get blown off use a bit of tape to keep them in place.

Draw a map of your classroom showing the locations of the air samplers. (use more paper if sampling more than one classroom)

Sample code:

Leave you air sampler out for about 30 days. – return them to us using the pre-paid envelope before the end of term.

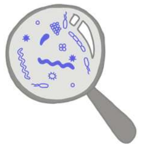

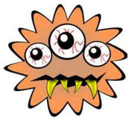

Fill out the table so we know about your sample

**School**			
**Email to send your results to**			
**Sample code (see tube)**	**Classroom**	**Date started**	**Date collected**

Part two: extracting your microbes

Use the gloves provided and try not to touch the air sampler or the inside of the tube, or the microbes from your skin will end up in the sample!

- Remove the dust cloth from your air sampler – *you do not need the paper part anymore*.
- Place the dust cloth into the tube provided (matching the codes). The liquid in the tube is just a 0.9% salt solution with tween, it is perfectly safe, but don't drink it – it will taste disgusting and probably make you puke.
- Shake for 1 minute.
- Post it back to us with this sheet.

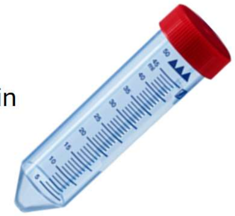

Thanks for your help! These results are very useful to our project and help us understand air quality in UK schools. We will send your results to the email provided

Which classroom do you think will have the best air? Why do you think this?

What is the good air? Are more or less microorganisms good, let me know what you think?

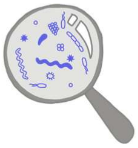

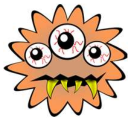

Sample code:

Sample code:
