# Supplementary material for: ‘Science Fun Days’: Opportunities for Connecting Primary School Pupils With Nature and Microbiology
Source: Microb Biotechnol. 2025 Dec 10;18(12):e70279. doi: 10.1111/1751-7915.70279 (PMC12696025; doi:10.1111/1751-7915.70279)

University of Essex

Science Fun Day

Name: ....

School: ....

MICROBIOLOGY SOCIETY

Schedule

**09:30 – 10:00**	**Arrival**
**10:00 – 10:45**	**Welcome assembly & quiz**
**10:45 – 11:00**	**Morning break**
**11:00 – 12:00**	**Session 1**
**12:00 – 12:45**	**Lunch**
**12:45 – 13:45**	**Session 2**
**13:45 – 14:30**	**Prizes & close**
**14:30 – 14:45**	**Back to coaches**

Session 1

Group A: STEM Lab

Group B: Outdoor activities

Session 2

Group A: Outdoor activities

Group B: STEM Lab

My Group: ....

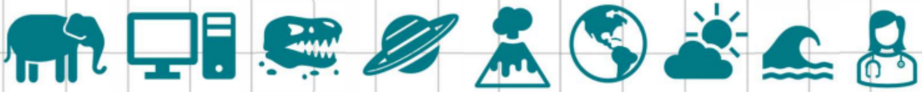

Name as many types of scientists as you can...

Marine biologist

Types of scientist

...use the icons as hints!

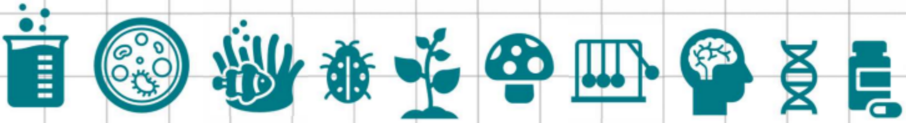

Can you find out these University of Essex facts?

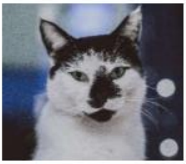

1. What is Campus Cat's real name?

....

2. What year was the University founded?

....

3. How much did the STEM Centre cost to build?

....

4. Name a scientist that at the University and what they study (hint: they will be running your sessions!):

Scientist: ....

Area of study: ....

....

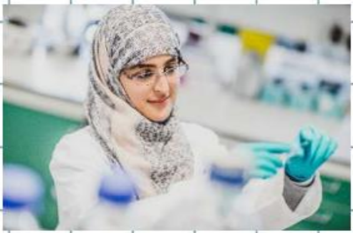
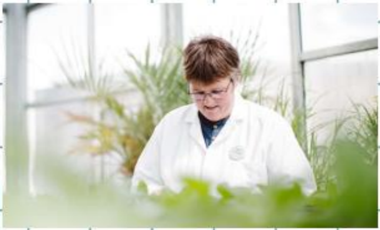

Nature treasure hunt

Deciduous tree

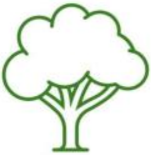

Name of tree:

Evergreen tree

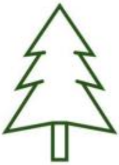

Name of tree:

Ant

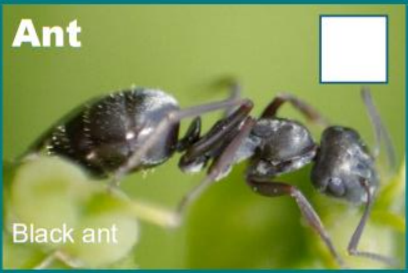

Black ant

Acorn

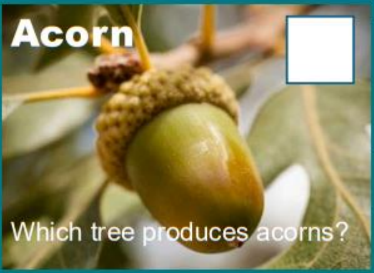

Which tree produces acorns?

Mushroom

(fungal fruiting body)

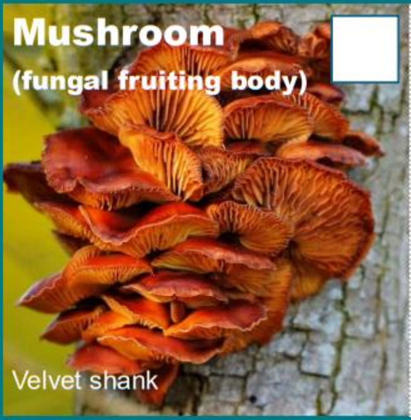

Velvet shank

Sketch your mushroom:

Image credit: Jay Burk (ant & fungi), Heather Gill via Unsplash (acorn)

Nature treasure hunt

Sketch two differently shaped leaves:

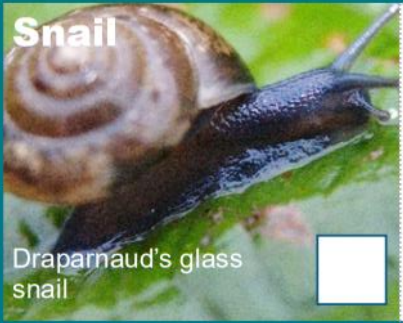

Sketch or describe its shell:

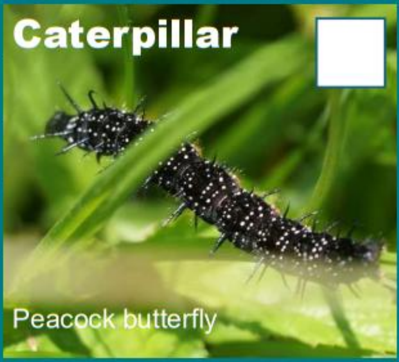

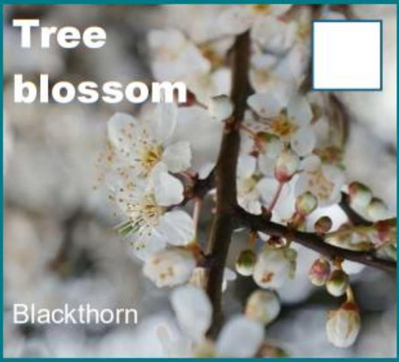

Image credit: Jay Burk (snail), Lars Willighagen via Wikimedia under the Creative Commons Attribution 4.0 International license (caterpillar)
Jack Blueberry via Unsplash (tree blossom)

Nature treasure hunt

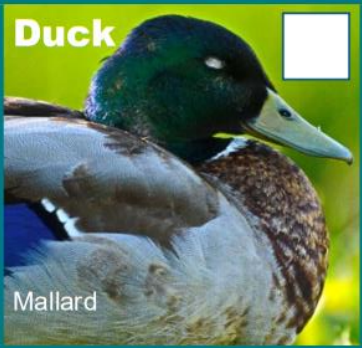

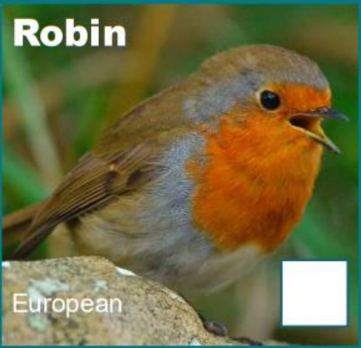

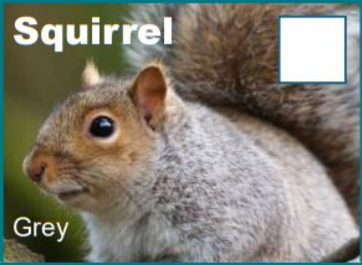

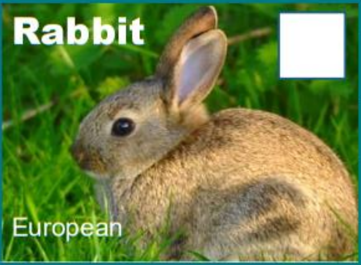

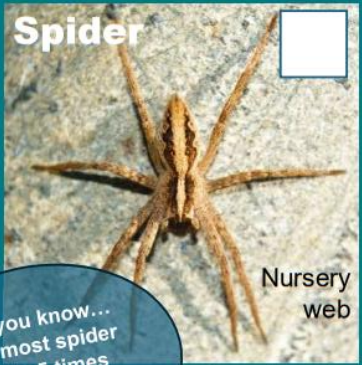

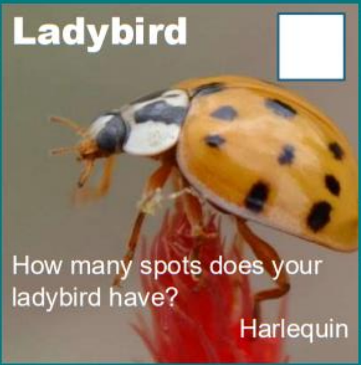

Did you know… that most spider silk is 5 times stronger than steel!

Image credit: Jay Burk (all)

Nature treasure hunt

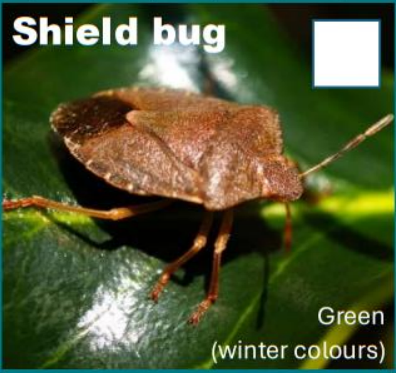

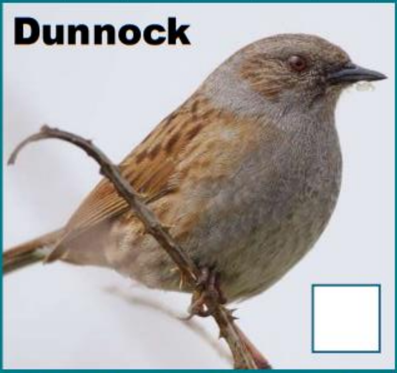

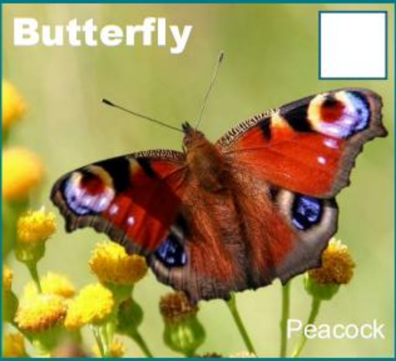

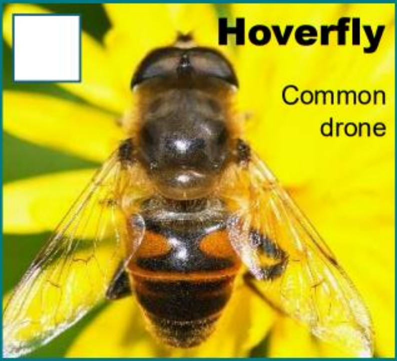

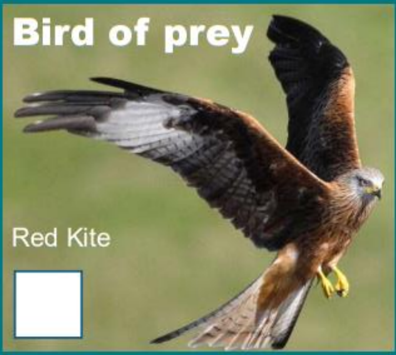

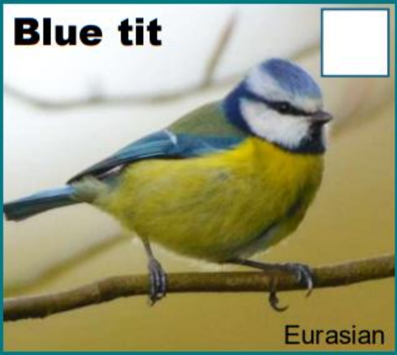

Image credit: Ed van Duijn via Unsplash (butterfly),
Jay Burk (remaining images)

Nature treasure hunt

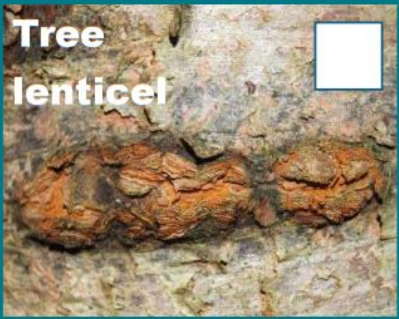

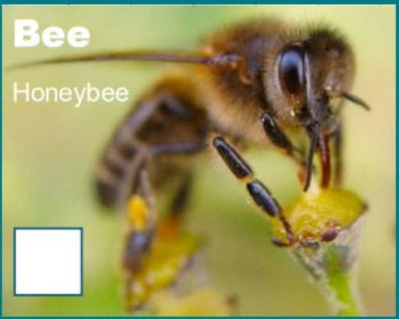

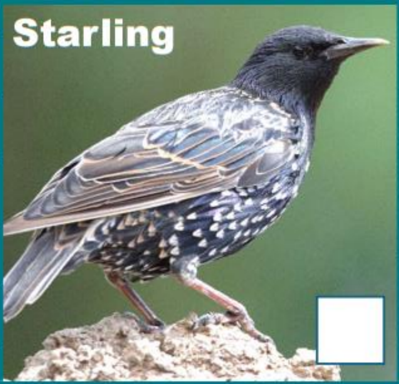

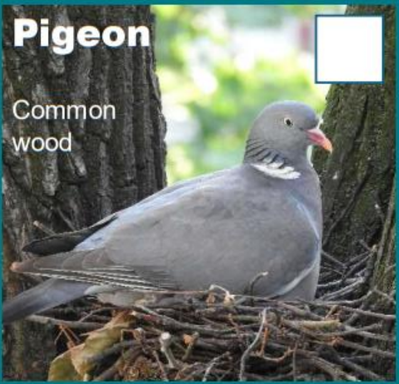

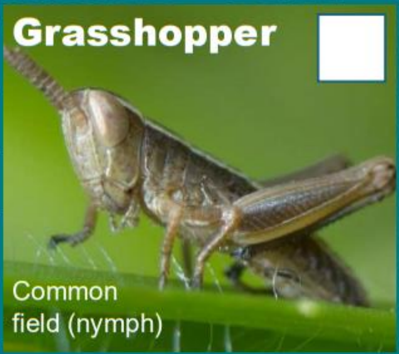

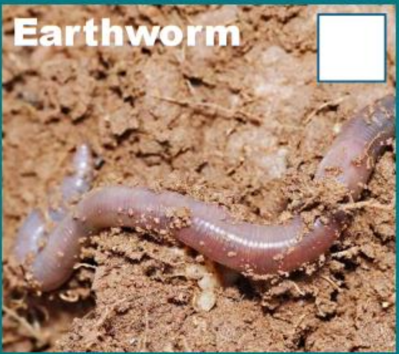

Image credit: Lukas Kadava via Unsplash (pigeon), Fir0002/Flagstaffotos via Wikimedia under the GNU Free License 1.2 (earthworm), Jay Burk (remaining images)

Nature treasure hunt

Other animals spotted:

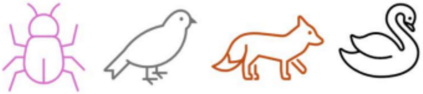

Other plants spotted:

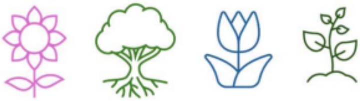

STEM Lab

Air quality

Name 5 sources of air pollution <u>in the home</u>:

1. ....
2. ....
3. ....
4. ....
5. ....

Tick the examples of <u>bio</u>aerosols:

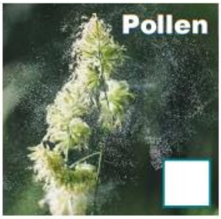

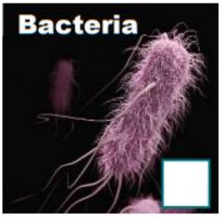

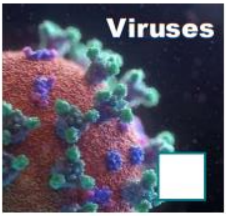

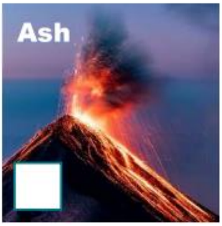

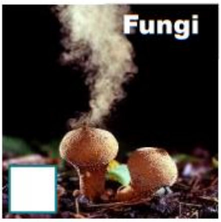

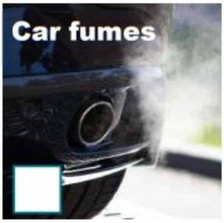

Unsplash image credit top: Alex Jones, CDC, Fusion Medical
bottom: Alain Bonnardeaux, Lesmalvern (via Wikimedia under the Creative Commons Attribution 4.0 International license), Matt Boitor

STEM Lab

Decomposition

Number these materials 1 to 9 in the order they would decompose in the environment from first to last:

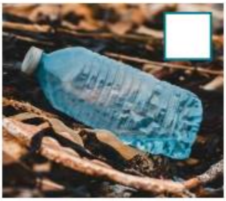
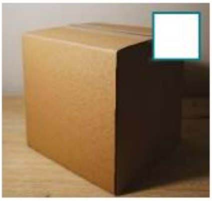
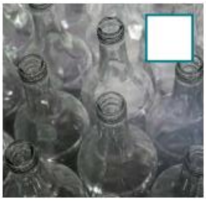
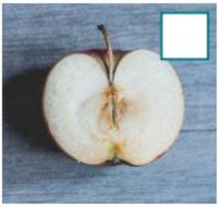
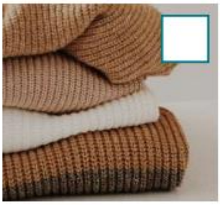
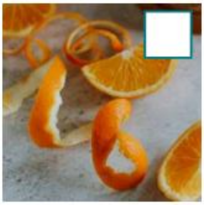
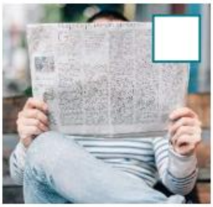
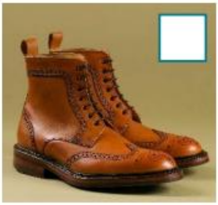
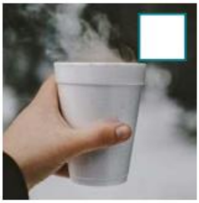

Which organisms help break materials down?

....

....

....

Unsplash image credit top: Erik Mclean, Brandable Box, A. R.
middle: Mak Flex, Tanya Trukyr, Gaby Yerden
bottom: Roman Kraft, Noah Smith, Caleb Lucas

My Notes

My Notes

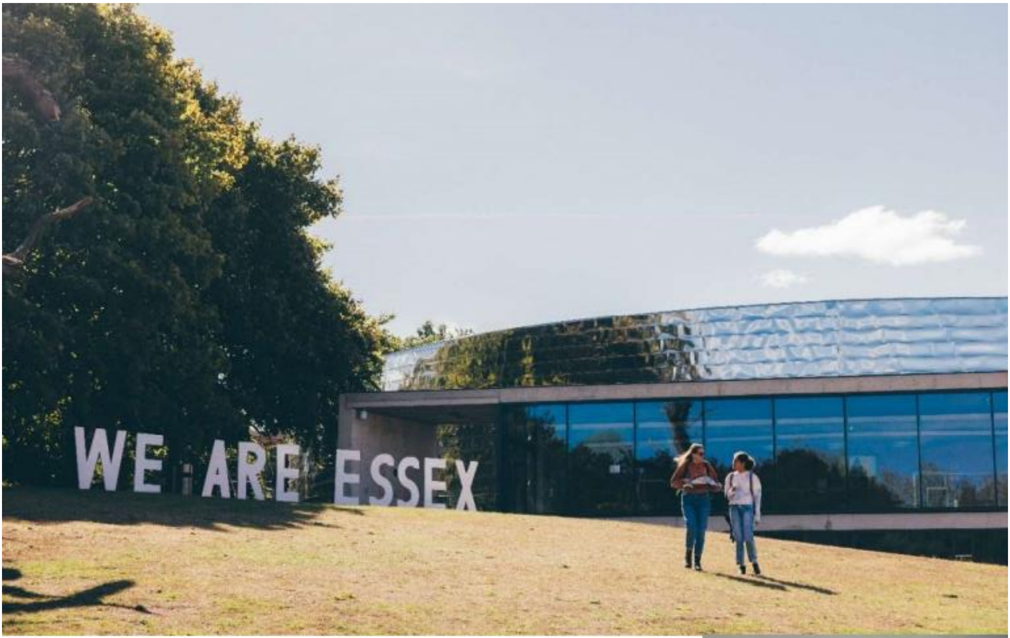

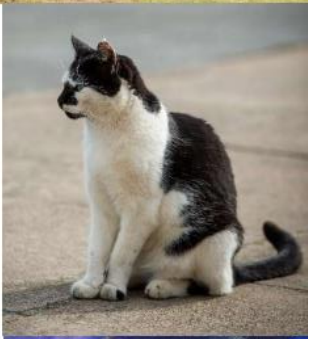

Thank you for all your hard work and enthusiasm today!

We hope you enjoyed your Science Fun Day!

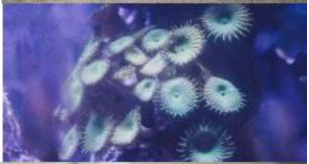

Funded by: MICROBIOLOGY SOCIETY

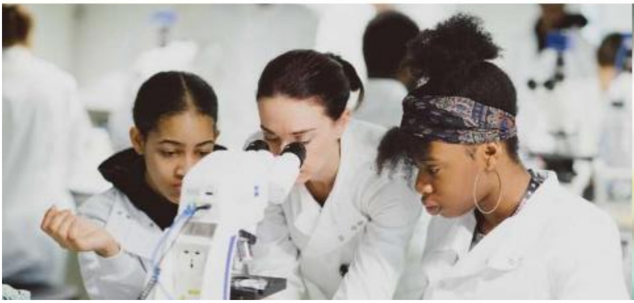

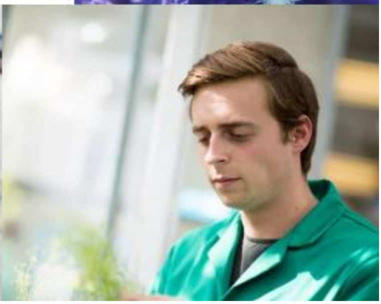

Supplement: Supplementary file 6 — Data S6: mbt270279‐sup‐0006‐DataS6.pdf. [file MBT2-18-e70279-s006.pdf]
