# Supplementary material for: ‘Science Fun Days’: Opportunities for Connecting Primary School Pupils With Nature and Microbiology
Source: Microb Biotechnol. 2025 Dec 10;18(12):e70279. doi: 10.1111/1751-7915.70279 (PMC12696025; doi:10.1111/1751-7915.70279)
Supplement: Supplementary file 9 — Appendix S1: mbt270279‐sup‐0009‐AppendixS1.docx. [file MBT2-18-e70279-s003.docx]

# Supporting information

**Table S 1** Ordinal logistic regression results tables for survey questions in 2024 and 2025.

| ***1. How much do you enjoy learning about science?*** | | | | | |
| --- | --- | --- | --- | --- | --- |
| **2024** | | | | | |
| **term** | **odds ratio** | **p-value** | **95% CI** | | **sig.** |
|  |  |  | **lower** | **upper** |  |
| **pre_postpost** | **2.07** | **0.050** | **1.00** | **4.32** | ***** |
| Sch_anonSchool 2 | 0.52 | 0.080 | 0.25 | 1.08 |  |
| Sch_anonSchool 3 | 0.82 | 0.591 | 0.41 | 1.67 |  |
| pre_postpost:Sch_anonSchool 2 | 0.75 | 0.586 | 0.27 | 2.10 |  |
| pre_postpost:Sch_anonSchool 3 | 0.67 | 0.452 | 0.24 | 1.89 |  |
|  |  |  |  |  |  |
| **2025** | | | | | |
| **term** | **odds ratio** | **p-value** | **95% CI** | | **sig.** |
|  |  |  | **lower** | **upper** |  |
| pre_postpost | 4.28 | 0.060 | 0.96 | 20.07 |  |
| **Sch_anonSchool 4** | **0.32** | **0.029** | **0.11** | **0.88** | ***** |
| **Sch_anonSchool 5** | **0.27** | **0.038** | **0.08** | **0.92** | ***** |
| **Sch_anonSchool 6** | **0.29** | **0.023** | **0.10** | **0.84** | ***** |
| **Sch_anonSchool 7** | **0.16** | **0.002** | **0.05** | **0.51** | ****** |
| gendermale | 1.57 | 0.213 | 0.77 | 3.17 |  |
| genderunspecified | 2.65 | 0.053 | 0.99 | 7.17 |  |
| pre_postpost:Sch_anonSchool 4 | 0.88 | 0.873 | 0.19 | 3.94 |  |
| pre_postpost:Sch_anonSchool 5 | 1.70 | 0.553 | 0.29 | 9.70 |  |
| pre_postpost:Sch_anonSchool 6 | 0.35 | 0.188 | 0.07 | 1.65 |  |
| pre_postpost:Sch_anonSchool 7 | 1.30 | 0.758 | 0.24 | 7.00 |  |
| pre_postpost:gendermale | 0.79 | 0.634 | 0.29 | 2.12 |  |
| **pre_postpost:genderunspecified** | **0.15** | **0.009** | **0.04** | **0.62** | ****** |
|  |  |  |  |  |  |
|  |  |  |  |  |  |
| ***2. How much do you want to go to university when you are older?*** | | | | | |
| **2024** | | | | | |
| **term** | **odds ratio** | **p-value** | **95% CI** | | **sig.** |
|  |  |  | **lower** | **upper** |  |
| pre_postpost | 1.60 | 0.182 | 0.80 | 3.19 |  |
| Sch_anonSchool 2 | 1.07 | 0.852 | 0.52 | 2.19 |  |
| Sch_anonSchool 3 | 0.66 | 0.235 | 0.33 | 1.31 |  |
| pre_postpost:Sch_anonSchool 2 | 0.89 | 0.816 | 0.33 | 2.41 |  |
| pre_postpost:Sch_anonSchool 3 | 0.78 | 0.627 | 0.29 | 2.11 |  |

Table S X continued…

| Table S1 continued…   \| ***2. How much do you want to go to university when you are older?*** \| \| \| \| \| \| \| --- \| --- \| --- \| --- \| --- \| --- \| \| **2025** \| \| \| \| \| \| \| **term** \| **odds ratio** \| **p-value** \| **95% CI** \| \| **sig.** \| \| **lower** \| **upper** \| \| pre_postpost \| 2.46 \| 0.222 \| 0.58 \| 10.52 \|  \| \| Sch_anonSchool 4 \| 2.77 \| 0.054 \| 0.98 \| 7.86 \|  \| \| Sch_anonSchool 5 \| 1.01 \| 0.983 \| 0.29 \| 3.58 \|  \| \| Sch_anonSchool 6 \| 1.08 \| 0.889 \| 0.37 \| 3.19 \|  \| \| Sch_anonSchool 7 \| 0.78 \| 0.661 \| 0.25 \| 2.40 \|  \| \| gendermale \| 0.55 \| 0.098 \| 0.27 \| 1.11 \|  \| \| genderunspecified \| 0.45 \| 0.109 \| 0.17 \| 1.20 \|  \| \| pre_postpost:Sch_anonSchool 4 \| 0.36 \| 0.166 \| 0.08 \| 1.52 \|  \| \| pre_postpost:Sch_anonSchool 5 \| 0.54 \| 0.482 \| 0.10 \| 3.00 \|  \| \| pre_postpost:Sch_anonSchool 6 \| 0.73 \| 0.691 \| 0.16 \| 3.37 \|  \| \| pre_postpost:Sch_anonSchool 7 \| 0.66 \| 0.604 \| 0.13 \| 3.24 \|  \| \| pre_postpost:gendermale \| 1.01 \| 0.979 \| 0.38 \| 2.69 \|  \| \| pre_postpost:genderunspecified \| 1.68 \| 0.459 \| 0.43 \| 6.63 \|  \| | | | | | |  |
| --- | --- | --- | --- | --- | --- | --- | --- | --- | --- | --- | --- | --- | --- | --- | --- | --- | --- | --- | --- | --- | --- | --- | --- | --- | --- | --- | --- | --- | --- | --- | --- | --- | --- | --- | --- | --- | --- | --- | --- | --- | --- | --- | --- | --- | --- | --- | --- | --- | --- | --- | --- | --- | --- | --- | --- | --- | --- | --- | --- | --- | --- | --- | --- | --- | --- | --- | --- | --- | --- | --- | --- | --- | --- | --- | --- | --- | --- | --- | --- | --- | --- | --- | --- | --- | --- | --- | --- | --- | --- | --- | --- | --- | --- | --- | --- | --- | --- | --- | --- | --- | --- | --- | --- | --- |
|  | | | | | |  |
|  | | | | | |  |
| ***3. How much would you like to have a job in science?*** | | | | | |  |
| **2024** | | | | | |  |
| **term** | **odds ratio** | **p-value** | **95% CI** | | **sig.** |  |
|  |  |  | **lower** | **upper** |  |  |
| **pre_postpost** | **2.24** | **0.024** | **1.11** | **4.53** | ***** |  |
| Sch_anonSchool 2 | 0.56 | 0.103 | 0.27 | 1.12 |  |  |
| Sch_anonSchool 3 | 1.75 | 0.116 | 0.87 | 3.54 |  |  |
| pre_postpost:Sch_anonSchool 2 | 0.87 | 0.781 | 0.33 | 2.31 |  |  |
| pre_postpost:Sch_anonSchool 3 | 0.50 | 0.166 | 0.18 | 1.34 |  |  |
|  | | | | | |  |
| **2025** | | | | | | |
| **term** | | **odds ratio** | **p-value** | **95% CI** | | **sig.** |
|  |  |  |  | **lower** | **upper** |  |
| **pre_postpost** | | **7.54** | **0.007** | **1.72** | **33.30** | ****** |
| Sch_anonSchool 4 | | 1.76 | 0.293 | 0.61 | 5.08 |  |
| Sch_anonSchool 5 | | 2.60 | 0.146 | 0.71 | 9.44 |  |
| Sch_anonSchool 6 | | 0.80 | 0.687 | 0.26 | 2.39 |  |
| Sch_anonSchool 7 | | 0.72 | 0.582 | 0.22 | 2.33 |  |
| gendermale | | 0.75 | 0.417 | 0.37 | 1.50 |  |
| genderunspecified | | 1.17 | 0.758 | 0.44 | 3.08 |  |
| pre_postpost:Sch_anonSchool 4 | | 0.24 | 0.059 | 0.06 | 1.06 |  |
| **pre_postpost:Sch_anonSchool 5** | | **0.12** | **0.018** | **0.02** | **0.69** | ***** |
| pre_postpost:Sch_anonSchool 6 | | 0.29 | 0.118 | 0.06 | 1.37 |  |
| pre_postpost:Sch_anonSchool 7 | | 0.45 | 0.347 | 0.09 | 2.35 |  |
| pre_postpost:gendermale | | 1.13 | 0.800 | 0.43 | 2.97 |  |
| pre_postpost:genderunspecified | | 1.07 | 0.920 | 0.27 | 4.20 |  |

Table S1 continued…

| ***4. How much do you enjoy learning about nature?*** | | | | | |
| --- | --- | --- | --- | --- | --- |
| **2024** | | | | | |
| **term** | **odds ratio** | **p-value** | **95% CI** | | **sig.** |
|  |  |  | **lower** | **upper** |  |
| pre_postpost | 0.96 | 0.910 | 0.45 | 2.03 |  |
| Sch_anonSchool 2 | 0.49 | 0.065 | 0.23 | 1.04 |  |
| Sch_anonSchool 3 | 1.35 | 0.418 | 0.65 | 2.83 |  |
| pre_postpost:Sch_anonSchool 2 | 1.26 | 0.663 | 0.45 | 3.55 |  |
| pre_postpost:Sch_anonSchool 3 | 1.34 | 0.594 | 0.46 | 3.99 |  |
|  |  |  |  |  |  |
| **2025** | | | | | |
| **term** | **odds ratio** | **p-value** | **95% CI** | | **sig.** |
|  |  |  | **lower** | **upper** |  |
| pre_postpost | 0.80 | 0.790 | 0.16 | 4.12 |  |
| Sch_anonSchool 4 | 0.35 | 0.056 | 0.11 | 1.00 |  |
| Sch_anonSchool 5 | 0.55 | 0.387 | 0.14 | 2.09 |  |
| **Sch_anonSchool 6** | **0.11** | **0.000** | **0.03** | **0.33** | ******* |
| **Sch_anonSchool 7** | **0.19** | **0.008** | **0.05** | **0.63** | ****** |
| **gendermale** | **0.23** | **0.000** | **0.11** | **0.48** | ******* |
| genderunspecified | 0.43 | 0.112 | 0.15 | 1.21 |  |
| pre_postpost:Sch_anonSchool 4 | 0.79 | 0.766 | 0.16 | 3.73 |  |
| pre_postpost:Sch_anonSchool 5 | 0.58 | 0.559 | 0.09 | 3.57 |  |
| pre_postpost:Sch_anonSchool 6 | 1.81 | 0.473 | 0.35 | 9.27 |  |
| pre_postpost:Sch_anonSchool 7 | 0.97 | 0.974 | 0.17 | 5.52 |  |
| pre_postpost:gendermale | 2.28 | 0.118 | 0.81 | 6.49 |  |
| pre_postpost:genderunspecified | 0.83 | 0.788 | 0.20 | 3.35 |  |
|  |  |  |  |  |  |
|  |  |  |  |  |  |
| ***5. How confident do you feel during science lessons?*** | | | | | |
| **2024** | | | | | |
| **term** | **odds ratio** | **p-value** | **95% CI** | | **sig.** |
|  |  |  | **lower** | **upper** |  |
| pre_postpost | 1.72 | 0.180 | 0.18 | 0.78 |  |
| Sch_anonSchool 2 | 0.50 | 0.104 | 0.10 | 0.22 |  |
| Sch_anonSchool 3 | 1.16 | 0.709 | 0.71 | 0.53 |  |
| pre_postpost:Sch_anonSchool 2 | 1.26 | 0.692 | 0.69 | 0.41 |  |
| pre_postpost:Sch_anonSchool 3 | 0.81 | 0.711 | 0.71 | 0.26 |  |

Table S1 continued…

| ***5. How confident do you feel during science lessons?*** | | | | | |  |
| --- | --- | --- | --- | --- | --- | --- |
| **2025** | | | | | |  |
| **term** | **odds ratio** | **p-value** | **95% CI** | | **sig.** |  |
|  |  |  | **lower** | **upper** |  |  |
| pre_postpost | 3.52 | 0.150 | 0.63 | 19.91 |  |  |
| Sch_anonSchool 4 | 0.87 | 0.830 | 0.24 | 2.96 |  |  |
| Sch_anonSchool 5 | 1.57 | 0.559 | 0.34 | 7.24 |  |  |
| Sch_anonSchool 6 | 0.62 | 0.461 | 0.17 | 2.16 |  |  |
| Sch_anonSchool 7 | 0.48 | 0.280 | 0.12 | 1.81 |  |  |
| **gendermale** | **2.66** | **0.021** | **1.16** | **6.15** | ***** |  |
| genderunspecified | 1.13 | 0.836 | 0.37 | 3.43 |  |  |
| pre_postpost:Sch_anonSchool 4 | 0.63 | 0.585 | 0.12 | 3.37 |  |  |
| pre_postpost:Sch_anonSchool 5 | 0.47 | 0.459 | 0.06 | 3.45 |  |  |
| pre_postpost:Sch_anonSchool 6 | 0.69 | 0.683 | 0.12 | 4.04 |  |  |
| pre_postpost:Sch_anonSchool 7 | 0.99 | 0.995 | 0.16 | 6.33 |  |  |
| pre_postpost:gendermale | 0.48 | 0.203 | 0.16 | 1.48 |  |  |
| pre_postpost:genderunspecified | 1.45 | 0.635 | 0.32 | 6.84 |  |  |
|  |  |  |  |  |  |  |
|  |  |  |  |  |  |  |
| ***6. How much do you know about microbes (bacteria, viruses, fungi)?*** | | | | | |  |
| **2024** | | | | | |  |
| **term** | **odds ratio** | **p-value** | **95% CI** | | **sig.** |  |
|  |  |  | **lower** | **upper** |  |  |
| **pre_postpost** | **5.00** | **0.000** | **2.38** | **10.63** | ******* |  |
| **Sch_anonSchool 2** | **2.64** | **0.012** | **1.25** | **5.65** | ****** |  |
| **Sch_anonSchool 3** | **2.98** | **0.004** | **1.42** | **6.33** | ****** |  |
| **pre_postpost:Sch_anonSchool 2** | **0.31** | **0.029** | **0.11** | **0.88** | ***** |  |
| **pre_postpost:Sch_anonSchool 3** | **0.32** | **0.034** | **0.11** | **0.91** | ***** |  |
|  | | | | | | |
| **2025** | | | | | | |
| **term** | | **odds ratio** | **p-value** | **95% CI** | | **sig.** |
|  |  |  |  | **lower** | **upper** |  |
| pre_postpost | | 4.17 | 0.060 | 0.95 | 18.90 |  |
| Sch_anonSchool 4 | | 0.49 | 0.143 | 0.19 | 1.27 |  |
| Sch_anonSchool 5 | | 0.43 | 0.177 | 0.13 | 1.46 |  |
| Sch_anonSchool 6 | | 0.56 | 0.248 | 0.20 | 1.50 |  |
| **Sch_anonSchool 7** | | **0.30** | **0.036** | **0.10** | **0.92** | ***** |
| gendermale | | 1.52 | 0.248 | 0.75 | 3.09 |  |
| **genderunspecified** | | **2.98** | **0.029** | **1.12** | **8.02** | ***** |
| pre_postpost:Sch_anonSchool 4 | | 1.29 | 0.736 | 0.29 | 5.54 |  |
| pre_postpost:Sch_anonSchool 5 | | 3.53 | 0.159 | 0.61 | 20.49 |  |
| pre_postpost:Sch_anonSchool 6 | | 1.56 | 0.573 | 0.33 | 7.30 |  |
| pre_postpost:Sch_anonSchool 7 | | 4.64 | 0.072 | 0.87 | 24.8 |  |
| pre_postpost:gendermale | | 0.75 | 0.574 | 0.27 | 2.05 |  |
| pre_postpost:genderunspecified | | 0.25 | 0.052 | 0.06 | 1.01 |  |

Table S1 continued…

| ***7. I like to do science activities*** | | | | | |
| --- | --- | --- | --- | --- | --- |
| **2025** | | | | | |
| **term** | **odds ratio** | **p-value** | **95% CI** | | **sig.** |
|  |  |  | **lower** | **upper** |  |
| **pre_postpost** | **7.26** | **0.011** | **1.60** | **35.07** | ***** |
| Sch_anonSchool 4 | 1.04 | 0.947 | 0.37 | 2.87 |  |
| Sch_anonSchool 5 | 1.36 | 0.643 | 0.37 | 4.97 |  |
| Sch_anonSchool 6 | 0.67 | 0.455 | 0.23 | 1.91 |  |
| **Sch_anonSchool 7** | **0.28** | **0.030** | **0.09** | **0.88** | ***** |
| gendermale | 1.12 | 0.760 | 0.55 | 2.25 |  |
| genderunspecified | 1.19 | 0.732 | 0.44 | 3.23 |  |
| pre_postpost:Sch_anonSchool 4 | 0.49 | 0.359 | 0.10 | 2.20 |  |
| pre_postpost:Sch_anonSchool 5 | 0.98 | 0.983 | 0.15 | 6.66 |  |
| **pre_postpost:Sch_anonSchool 6** | **0.20** | **0.046** | **0.04** | **0.96** | ***** |
| pre_postpost:Sch_anonSchool 7 | 0.44 | 0.343 | 0.08 | 2.35 |  |
| pre_postpost:gendermale | 0.57 | 0.275 | 0.20 | 1.57 |  |
| pre_postpost:genderunspecified | 0.32 | 0.124 | 0.07 | 1.37 |  |

| ***8. I want to understand how nature works*** | | | | | |
| --- | --- | --- | --- | --- | --- |
| **2025** | | | | | |
| **term** | **odds ratio** | **p-value** | **95% CI** | | **sig.** |
|  |  |  | **lower** | **upper** |  |
| pre_postpost | 1.33 | 0.720 | 0.28 | 6.38 |  |
| Sch_anonSchool 4 | 0.39 | 0.089 | 0.13 | 1.13 |  |
| Sch_anonSchool 5 | 0.49 | 0.291 | 0.13 | 1.81 |  |
| **Sch_anonSchool 6** | **0.17** | **0.002** | **0.06** | **0.51** | ****** |
| **Sch_anonSchool 7** | **0.13** | **0.001** | **0.04** | **0.42** | ******* |
| **gendermale** | **0.45** | **0.028** | **0.22** | **0.91** | ***** |
| genderunspecified | 1.02 | 0.965 | 0.39 | 2.71 |  |
| pre_postpost:Sch_anonSchool 4 | 0.42 | 0.269 | 0.09 | 1.94 |  |
| pre_postpost:Sch_anonSchool 5 | 0.57 | 0.542 | 0.09 | 3.44 |  |
| pre_postpost:Sch_anonSchool 6 | 0.75 | 0.721 | 0.15 | 3.66 |  |
| pre_postpost:Sch_anonSchool 7 | 0.97 | 0.974 | 0.18 | 5.20 |  |
| pre_postpost:gendermale | 1.89 | 0.206 | 0.71 | 5.06 |  |
| pre_postpost:genderunspecified | 0.42 | 0.204 | 0.11 | 1.61 |  |

Table S1 continued…

| ***9. I enjoy reading about science and nature*** | | | | | |
| --- | --- | --- | --- | --- | --- |
| **2025** | | | | | |
| **term** | **odds ratio** | **p-value** | **95% CI** | | **sig.** |
|  |  |  | **lower** | **upper** |  |
| pre_postpost | 3.69 | 0.091 | 0.81 | 17.06 |  |
| Sch_anonSchool 4 | 0.39 | 0.084 | 0.13 | 1.13 |  |
| Sch_anonSchool 5 | 0.75 | 0.678 | 0.19 | 2.91 |  |
| **Sch_anonSchool 6** | **0.21** | **0.007** | **0.07** | **0.64** | ****** |
| **Sch_anonSchool 7** | **0.10** | **0.000** | **0.03** | **0.34** | ******* |
| gendermale | 1.91 | 0.068 | 0.95 | 3.86 |  |
| genderunspecified | 1.07 | 0.891 | 0.41 | 2.78 |  |
| pre_postpost:Sch_anonSchool 4 | 0.50 | 0.363 | 0.11 | 2.22 |  |
| pre_postpost:Sch_anonSchool 5 | 0.32 | 0.205 | 0.05 | 1.87 |  |
| pre_postpost:Sch_anonSchool 6 | 0.37 | 0.208 | 0.08 | 1.74 |  |
| pre_postpost:Sch_anonSchool 7 | 0.64 | 0.601 | 0.12 | 3.45 |  |
| pre_postpost:gendermale | 0.55 | 0.225 | 0.21 | 1.45 |  |
| pre_postpost:genderunspecified | 0.69 | 0.592 | 0.18 | 2.65 |  |

| ***10. I enjoy talking about science and nature*** | | | | | |
| --- | --- | --- | --- | --- | --- |
| **2025** | | | | | |
| **term** | **odds ratio** | **p-value** | **95% CI** | | **sig.** |
|  |  |  | **lower** | **upper** |  |
| pre_postpost | 1.72 | 0.457 | 0.41 | 7.24 |  |
| Sch_anonSchool 4 | 0.55 | 0.233 | 0.20 | 1.47 |  |
| Sch_anonSchool 5 | 1.10 | 0.883 | 0.32 | 3.77 |  |
| **Sch_anonSchool 6** | **0.34** | **0.038** | **0.12** | **0.94** | ***** |
| **Sch_anonSchool 7** | **0.18** | **0.003** | **0.06** | **0.55** | ****** |
| gendermale | 0.79 | 0.514 | 0.40 | 1.59 |  |
| genderunspecified | 0.78 | 0.610 | 0.29 | 2.04 |  |
| pre_postpost:Sch_anonSchool 4 | 0.74 | 0.681 | 0.18 | 3.08 |  |
| pre_postpost:Sch_anonSchool 5 | 0.28 | 0.132 | 0.05 | 1.46 |  |
| pre_postpost:Sch_anonSchool 6 | 0.82 | 0.794 | 0.19 | 3.59 |  |
| pre_postpost:Sch_anonSchool 7 | 1.46 | 0.640 | 0.30 | 7.05 |  |
| pre_postpost:gendermale | 1.39 | 0.510 | 0.52 | 3.67 |  |
| pre_postpost:genderunspecified | 1.53 | 0.532 | 0.41 | 5.83 |  |

Table S1 continued…

| ***11. I enjoy watching science and nature shows on TV*** | | | | | |
| --- | --- | --- | --- | --- | --- |
| **2025** | | | | | |
| **term** | **odds ratio** | **p-value** | **95% CI** | | **sig.** |
|  |  |  | **lower** | **upper** |  |
| **pre_postpost** | **5.71** | **0.014** | **1.44** | **23.65** | ***** |
| Sch_anonSchool 4 | 1.16 | 0.762 | 0.43 | 3.12 |  |
| Sch_anonSchool 5 | 1.11 | 0.871 | 0.33 | 3.75 |  |
| Sch_anonSchool 6 | 0.79 | 0.652 | 0.29 | 2.17 |  |
| Sch_anonSchool 7 | 0.40 | 0.097 | 0.14 | 1.18 |  |
| gendermale | 0.87 | 0.688 | 0.44 | 1.71 |  |
| genderunspecified | 0.62 | 0.312 | 0.25 | 1.56 |  |
| pre_postpost:Sch_anonSchool 4 | 0.35 | 0.146 | 0.08 | 1.42 |  |
| **pre_postpost:Sch_anonSchool 5** | **0.09** | **0.005** | **0.02** | **0.48** | ****** |
| **pre_postpost:Sch_anonSchool 6** | **0.18** | **0.023** | **0.04** | **0.78** | ***** |
| pre_postpost:Sch_anonSchool 7 | 0.31 | 0.133 | 0.06 | 1.42 |  |
| pre_postpost:gendermale | 0.93 | 0.888 | 0.36 | 2.40 |  |
| pre_postpost:genderunspecified | 0.88 | 0.850 | 0.24 | 3.26 |  |

| ***12. I am good at understanding some science topics*** | | | | | |
| --- | --- | --- | --- | --- | --- |
| **2025** | | | | | |
| **term** | **odds ratio** | **p-value** | **95% CI** | | **sig.** |
|  |  |  | **lower** | **upper** |  |
| **pre_postpost** | **7.43** | **0.006** | **1.79** | **31.74** | ****** |
| Sch_anonSchool 4 | 0.88 | 0.795 | 0.34 | 2.29 |  |
| Sch_anonSchool 5 | 1.20 | 0.763 | 0.36 | 4.06 |  |
| Sch_anonSchool 6 | 0.75 | 0.561 | 0.28 | 1.99 |  |
| Sch_anonSchool 7 | 1.01 | 0.986 | 0.34 | 3.04 |  |
| **gendermale** | **2.52** | **0.010** | **1.24** | **5.11** | ***** |
| genderunspecified | 2.01 | 0.170 | 0.74 | 5.44 |  |
| pre_postpost:Sch_anonSchool 4 | 0.37 | 0.170 | 0.09 | 1.51 |  |
| pre_postpost:Sch_anonSchool 5 | 0.35 | 0.228 | 0.06 | 1.92 |  |
| **pre_postpost:Sch_anonSchool 6** | **0.22** | **0.049** | **0.05** | **0.98** | ***** |
| pre_postpost:Sch_anonSchool 7 | 0.33 | 0.182 | 0.07 | 1.66 |  |
| pre_postpost:gendermale | 0.68 | 0.439 | 0.25 | 1.82 |  |
| pre_postpost:genderunspecified | 0.43 | 0.236 | 0.11 | 1.73 |  |

Table S1 continued…

| ***13. I am good at explaining science*** | | | | | |
| --- | --- | --- | --- | --- | --- |
| **2025** | | | | | |
| **term** | **odds ratio** | **p-value** | **95% CI** | | **sig.** |
|  |  |  | **lower** | **upper** |  |
| **pre_postpost** | **11.79** | **0.000** | **3.00** | **48.09** | ******* |
| Sch_anonSchool 4 | 0.91 | 0.846 | 0.36 | 2.30 |  |
| Sch_anonSchool 5 | 0.76 | 0.654 | 0.23 | 2.48 |  |
| Sch_anonSchool 6 | 0.88 | 0.785 | 0.34 | 2.27 |  |
| Sch_anonSchool 7 | 0.66 | 0.436 | 0.23 | 1.88 |  |
| **gendermale** | **2.44** | **0.013** | **1.21** | **4.95** | ***** |
| genderunspecified | 2.45 | 0.077 | 0.90 | 6.65 |  |
| pre_postpost:Sch_anonSchool 4 | 0.27 | 0.056 | 0.07 | 1.02 |  |
| **pre_postpost:Sch_anonSchool 5** | **0.17** | **0.034** | **0.03** | **0.87** | ***** |
| **pre_postpost:Sch_anonSchool 6** | **0.20** | **0.029** | **0.05** | **0.84** | ***** |
| pre_postpost:Sch_anonSchool 7 | 0.32 | 0.143 | 0.07 | 1.47 |  |
| pre_postpost:gendermale | 0.55 | 0.229 | 0.21 | 1.45 |  |
| pre_postpost:genderunspecified | 0.37 | 0.152 | 0.10 | 1.44 |  |

| ***14. I am good at following instructions for scientific activities*** | | | | | |
| --- | --- | --- | --- | --- | --- |
| **2025** | | | | | |
| **term** | **odds ratio** | **p-value** | **95% CI** | | **sig.** |
|  |  |  | **lower** | **upper** |  |
| pre_postpost | 2.37 | 0.272 | 0.51 | 11.39 |  |
| Sch_anonSchool 4 | 0.78 | 0.646 | 0.27 | 2.22 |  |
| Sch_anonSchool 5 | 1.61 | 0.490 | 0.42 | 6.29 |  |
| Sch_anonSchool 6 | 0.38 | 0.090 | 0.12 | 1.15 |  |
| Sch_anonSchool 7 | 0.43 | 0.160 | 0.13 | 1.38 |  |
| gendermale | 0.68 | 0.282 | 0.33 | 1.37 |  |
| genderunspecified | 0.40 | 0.082 | 0.14 | 1.12 |  |
| pre_postpost:Sch_anonSchool 4 | 0.64 | 0.572 | 0.14 | 2.94 |  |
| pre_postpost:Sch_anonSchool 5 | 0.32 | 0.231 | 0.05 | 2.03 |  |
| pre_postpost:Sch_anonSchool 6 | 0.31 | 0.149 | 0.06 | 1.51 |  |
| pre_postpost:Sch_anonSchool 7 | 0.70 | 0.675 | 0.13 | 3.76 |  |
| pre_postpost:gendermale | 0.98 | 0.974 | 0.36 | 2.66 |  |
| pre_postpost:genderunspecified | 1.86 | 0.392 | 0.45 | 7.76 |  |

Table S1 continued…

| ***20. I am proud I helped scientists*** | | | | | |
| --- | --- | --- | --- | --- | --- |
| **2025** | | | | | |
| **term** | **odds ratio** | **p-value** | **95% CI** | | **sig.** |
|  |  |  | **lower** | **upper** |  |
| Sch_anonSchool 4 | 1.33 | 0.647 | 0.37 | 4.49 |  |
| Sch_anonSchool 5 | 1.88 | 0.390 | 0.44 | 8.29 |  |
| Sch_anonSchool 6 | 0.46 | 0.198 | 0.13 | 1.46 |  |
| Sch_anonSchool 7 | 0.45 | 0.220 | 0.12 | 1.58 |  |
| gendermale | 0.94 | 0.876 | 0.45 | 1.98 |  |
| genderunspecified | 1.00 | 0.995 | 0.28 | 4.19 |  |

| ***21. I understand better how scientists work*** | | | | | |
| --- | --- | --- | --- | --- | --- |
| **2025** | | | | | |
| **term** | **odds ratio** | **p-value** | **95% CI** | | **sig.** |
|  |  |  | **lower** | **upper** |  |
| Sch_anonSchool 4 | 0.46 | 0.176 | 0.15 | 1.40 |  |
| Sch_anonSchool 5 | 0.74 | 0.629 | 0.21 | 2.56 |  |
| Sch_anonSchool 6 | 0.36 | 0.079 | 0.11 | 1.11 |  |
| Sch_anonSchool 7 | 0.41 | 0.146 | 0.12 | 1.36 |  |
| gendermale | 1.47 | 0.284 | 0.73 | 3.00 |  |
| genderunspecified | 2.38 | 0.142 | 0.76 | 7.82 |  |

| ***22. I will tell my family and friends about the activities*** | | | | | |
| --- | --- | --- | --- | --- | --- |
| **2025** | | | | | |
| **term** | **odds ratio** | **p-value** | **95% CI** | | **sig.** |
|  |  |  | **lower** | **upper** |  |
| Sch_anonSchool 4 | 0.90 | 0.858 | 0.27 | 2.86 |  |
| Sch_anonSchool 5 | 2.81 | 0.171 | 0.66 | 13.36 |  |
| Sch_anonSchool 6 | 0.50 | 0.232 | 0.15 | 1.53 |  |
| Sch_anonSchool 7 | 0.67 | 0.521 | 0.19 | 2.28 |  |
| gendermale | 0.52 | 0.091 | 0.24 | 1.10 |  |
| genderunspecified | 0.50 | 0.270 | 0.15 | 1.78 |  |

Table S1 continued…

| ***23. I would like to do these kinds of activities more in class*** | | | | | |
| --- | --- | --- | --- | --- | --- |
| **2025** | | | | | |
| **term** | **odds ratio** | **p-value** | **95% CI** | | **sig.** |
|  |  |  | **lower** | **upper** |  |
| Sch_anonSchool 4 | 0.30 | 0.094 | 0.06 | 1.12 |  |
| Sch_anonSchool 5 | 0.87 | 0.868 | 0.15 | 4.69 |  |
| **Sch_anonSchool 6** | **0.12** | **0.003** | **0.02** | **0.44** | ****** |
| Sch_anonSchool 7 | 0.34 | 0.172 | 0.06 | 1.49 |  |
| gendermale | 0.60 | 0.197 | 0.27 | 1.30 |  |
| genderunspecified | 0.62 | 0.462 | 0.18 | 2.37 |  |
| ***24. It was fun to participate in the scientific activities*** | | | | | |
| **2025** | | | | | |
| **term** | **odds ratio** | **p-value** | **95% CI** | | **sig.** |
|  |  |  | **lower** | **upper** |  |
| **Sch_anonSchool 4** | **0.10** | **0.032** | **0.01** | **0.57** | ***** |
| Sch_anonSchool 5 | 0.40 | 0.454 | 0.02 | 3.64 |  |
| **Sch_anonSchool 6** | **0.06** | **0.009** | **0.00** | **0.33** | ****** |
| **Sch_anonSchool 7** | **0.11** | **0.049** | **0.01** | **0.71** | ***** |
| gendermale | 0.70 | 0.403 | 0.30 | 1.61 |  |
| **genderunspecified** | **0.23** | **0.021** | **0.06** | **0.81** | ***** |

**Table S2** Ordinal logistic regression results tables for survey questions in 2025 with school groups assigned to ‘low-deprivation-location’ or ‘high-deprivation-location’ group based on Index of Multiple Deprivation (IMD) of school postcode.

| ***1. How much do you enjoy learning about science?*** | | | | | |
| --- | --- | --- | --- | --- | --- |
| **2025** | | | | | |
| **term** | **odds ratio** | **p-value** | **95% CI** | | **sig.** |
|  |  |  | **lower** | **upper** |  |
| pre_postpost | 1.55 | 0.223 | 0.77 | 3.13 |  |
| imd_grouphigh-deprivation-location | 0.69 | 0.222 | 0.38 | 1.25 |  |
| pre_postpost:imd_grouphigh-deprivation-location | 1.68 | 0.241 | 0.71 | 4.02 |  |
|  |  |  |  |  |  |
|  |  |  |  |  |  |
| ***2. How much do you want to go to university when you are older?*** | | | | | |
| **2025** | | | | | |
| **term** | **odds ratio** | **p-value** | **95% CI** | | **sig.** |
|  |  |  | **lower** | **upper** |  |
| pre_postpost | 2.02 | 0.053 | 0.99 | 4.14 |  |
| imd_grouphigh-deprivation-location | 1.37 | 0.316 | 0.74 | 2.52 |  |
| pre_postpost:imd_grouphigh-deprivation-location | 0.71 | 0.443 | 0.30 | 1.70 |  |

| ***3. How much would you like to have a job in science?*** | | | | | |
| --- | --- | --- | --- | --- | --- |
| **2025** | | | | | |
| **term** | **odds ratio** | **p-value** | **95% CI** | | **sig.** |
|  |  |  | **lower** | **upper** |  |
| **pre_postpost** | **3.37** | **0.001** | **1.65** | **6.94** | ******* |
| **imd_grouphigh-deprivation-location** | **1.98** | **0.029** | **1.07** | **3.66** | ***** |
| pre_postpost:imd_grouphigh-deprivation-location | 0.54 | 0.161 | 0.23 | 1.28 |  |

| ***4. How much do you enjoy learning about nature?*** | | | | | |
| --- | --- | --- | --- | --- | --- |
| **2025** | | | | | |
| **term** | **odds ratio** | **p-value** | **95% CI** | | **sig.** |
|  |  |  | **lower** | **upper** |  |
| **pre_postpost** | **2.13** | **0.039** | **1.04** | **4.37** | ***** |
| imd_grouphigh-deprivation-location | 1.78 | 0.062 | 0.97 | 3.27 |  |
| pre_postpost:imd_grouphigh-deprivation-location | 0.46 | 0.079 | 0.19 | 1.09 |  |

Table S2 continued…

| ***5. How confident do you feel during science lessons?*** | | | | | |
| --- | --- | --- | --- | --- | --- |
| **2025** | | | | | |
| **term** | **odds ratio** | **p-value** | **95% CI** | | **sig.** |
|  |  |  | **lower** | **upper** |  |
| pre_postpost | 1.73 | 0.172 | 0.79 | 3.84 |  |
| imd_grouphigh-deprivation-location | 0.97 | 0.923 | 0.49 | 1.90 |  |
| pre_postpost:imd_grouphigh-deprivation-location | 0.98 | 0.972 | 0.37 | 2.59 |  |
|  |  |  |  |  |  |
|  |  |  |  |  |  |
| ***6. How much do you know about microbes (bacteria, viruses, fungi)?*** | | | | | |
| **2025** | | | | | |
| **term** | **odds ratio** | **p-value** | **95% CI** | | **sig.** |
|  |  |  | **lower** | **upper** |  |
| **pre_postpost** | **3.91** | **0.000** | **1.87** | **8.28** | ******* |
| imd_grouphigh-deprivation-location | 0.69 | 0.217 | 0.38 | 1.25 |  |
| pre_postpost:imd_grouphigh-deprivation-location | 1.44 | 0.429 | 0.58 | 3.52 |  |

| ***7. I like to do science activities*** | | | | | |
| --- | --- | --- | --- | --- | --- |
| **2025** | | | | | |
| **term** | **odds ratio** | **p-value** | **95% CI** | | **sig.** |
|  |  |  | **lower** | **upper** |  |
| pre_postpost | 1.54 | 0.230 | 0.76 | 3.10 |  |
| imd_grouphigh-deprivation-location | 1.08 | 0.809 | 0.59 | 1.96 |  |
| pre_postpost:imd_grouphigh-deprivation-location | 1.48 | 0.381 | 0.62 | 3.57 |  |

| ***8. I want to understand how nature works*** | | | | | |
| --- | --- | --- | --- | --- | --- |
| **2025** | | | | | |
| **term** | **odds ratio** | **p-value** | **95% CI** | | **sig.** |
|  |  |  | **lower** | **upper** |  |
| pre_postpost | 1.67 | 0.143 | 0.84 | 3.34 |  |
| imd_grouphigh-deprivation-location | 1.31 | 0.370 | 0.73 | 2.36 |  |
| pre_postpost:imd_grouphigh-deprivation-location | 0.52 | 0.136 | 0.22 | 1.23 |  |

Table S2 continued…

| ***9. I enjoy reading about science and nature*** | | | | | |
| --- | --- | --- | --- | --- | --- |
| **2025** | | | | | |
| **term** | **odds ratio** | **p-value** | **95% CI** | | **sig.** |
|  |  |  | **lower** | **upper** |  |
| pre_postpost | 1.40 | 0.337 | 0.70 | 2.81 |  |
| imd_grouphigh-deprivation-location | 0.93 | 0.804 | 0.51 | 1.67 |  |
| pre_postpost:imd_grouphigh-deprivation-location | 0.85 | 0.706 | 0.36 | 1.99 |  |
|  |  |  |  |  |  |
|  |  |  |  |  |  |
| ***10. I enjoy talking about science and nature*** | | | | | |
| **2025** | | | | | |
| **term** | **odds ratio** | **p-value** | **95% CI** | | **sig.** |
|  |  |  | **lower** | **upper** |  |
| pre_postpost | 1.96 | 0.054 | 0.99 | 3.90 |  |
| imd_grouphigh-deprivation-location | 1.03 | 0.925 | 0.57 | 1.85 |  |
| pre_postpost:imd_grouphigh-deprivation-location | 0.74 | 0.494 | 0.32 | 1.73 |  |

| ***11. I enjoy watching science and nature shows on TV*** | | | | | |
| --- | --- | --- | --- | --- | --- |
| **2025** | | | | | |
| **term** | **odds ratio** | **p-value** | **95% CI** | | **sig.** |
|  |  |  | **lower** | **upper** |  |
| pre_postpost | 1.71 | 0.122 | 0.87 | 3.37 |  |
| imd_grouphigh-deprivation-location | 0.93 | 0.820 | 0.52 | 1.68 |  |
| pre_postpost:imd_grouphigh-deprivation-location | 0.78 | 0.555 | 0.34 | 1.80 |  |

| ***12. I am good at understanding some science topics*** | | | | | |
| --- | --- | --- | --- | --- | --- |
| **2025** | | | | | |
| **term** | **odds ratio** | **p-value** | **95% CI** | | **sig.** |
|  |  |  | **lower** | **upper** |  |
| **pre_postpost** | **2.15** | **0.037** | **1.05** | **4.43** | ***** |
| imd_grouphigh-deprivation-location | 1.14 | 0.668 | 0.63 | 2.06 |  |
| pre_postpost:imd_grouphigh-deprivation-location | 0.77 | 0.562 | 0.32 | 1.86 |  |

Table S2 continued…

| ***13. I am good at explaining science*** | | | | | |
| --- | --- | --- | --- | --- | --- |
| **2025** | | | | | |
| **term** | **odds ratio** | **p-value** | **95% CI** | | **sig.** |
|  |  |  | **lower** | **upper** |  |
| **pre_postpost** | **2.47** | **0.009** | **1.26** | **4.90** | ****** |
| imd_grouphigh-deprivation-location | 0.84 | 0.557 | 0.47 | 1.50 |  |
| pre_postpost:imd_grouphigh-deprivation-location | 0.68 | 0.364 | 0.29 | 1.57 |  |
|  |  |  |  |  |  |
|  |  |  |  |  |  |
| ***14. I am good at following instructions for scientific activities*** | | | | | |
| **2025** | | | | | |
| **term** | **odds ratio** | **p-value** | **95% CI** | | **sig.** |
|  |  |  | **lower** | **upper** |  |
| pre_postpost | 1.07 | 0.846 | 0.53 | 2.19 |  |
| imd_grouphigh-deprivation-location | 1.34 | 0.347 | 0.73 | 2.49 |  |
| pre_postpost:imd_grouphigh-deprivation-location | 1.49 | 0.375 | 0.62 | 3.60 |  |

| ***20. I am proud I helped scientists*** | | | | | |
| --- | --- | --- | --- | --- | --- |
| **2025** | | | | | |
| **term** | **odds ratio** | **p-value** | **95% CI** | | **sig.** |
|  |  |  | **lower** | **upper** |  |
| imd_grouphigh-deprivation-location | 1.80 | 0.092 | 0.91 | 3.56 |  |

| ***21. I understand better how scientists work*** | | | | | |
| --- | --- | --- | --- | --- | --- |
| **2025** | | | | | |
| **term** | **odds ratio** | **p-value** | **95% CI** | | **sig.** |
|  |  |  | **lower** | **upper** |  |
| imd_grouphigh-deprivation-location | 0.97 | 0.936 | 0.50 | 1.88 |  |

| ***22. I will tell my family and friends about the activities*** | | | | | |
| --- | --- | --- | --- | --- | --- |
| **2025** | | | | | |
| **term** | **odds ratio** | **p-value** | **95% CI** | | **sig.** |
|  |  |  | **lower** | **upper** |  |
| imd_grouphigh-deprivation-location | 1.86 | 0.072 | 0.95 | 3.66 |  |

Table S2 continued…

|  |  |  |  |  |  |
| --- | --- | --- | --- | --- | --- |
| ***23. I would like to do these kinds of activities more in class*** | | | | | |
| **2025** | | | | | |
| **term** | **odds ratio** | **p-value** | **95% CI** | | **sig.** |
|  |  |  | **lower** | **upper** |  |
| **imd_grouphigh-deprivation-location** | **2.07** | **0.036** | **1.05** | **4.12** | ***** |

| ***24. It was fun to participate in the scientific activities*** | | | | | |
| --- | --- | --- | --- | --- | --- |
| **2025** | | | | | |
| **term** | **odds ratio** | **p-value** | **95% CI** | | **sig.** |
|  |  |  | **lower** | **upper** |  |
| imd_grouphigh-deprivation-location | 1.15 | 0.708 | 0.55 | 2.37 |  |


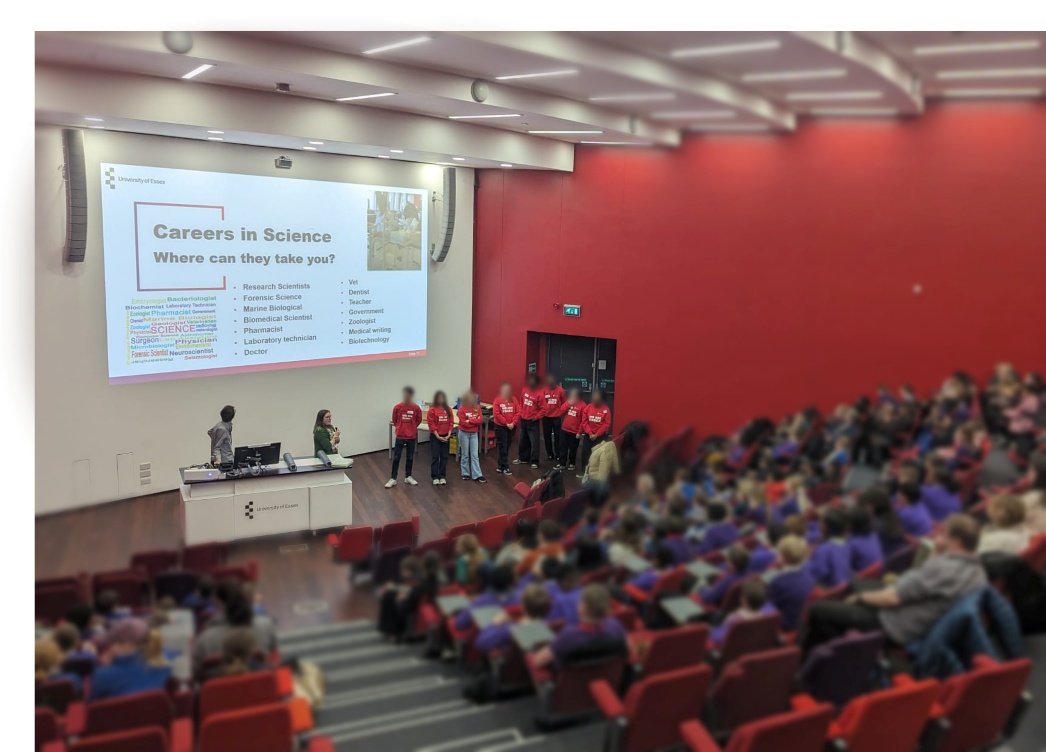


**Figure S 1** Pupils being introduced different science-related careers in a University of Essex lecture hall. Student Ambassadors are to the side in red shirts.


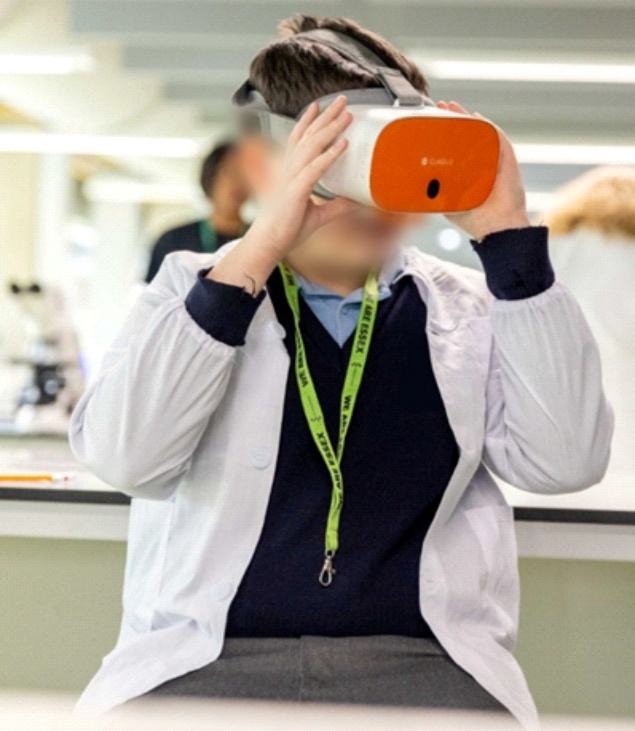


**Figure S 2** Pupils were able to ‘step into’ different University of Essex practical classes using a virtual reality (VR) headset to experience what is it like to be a student studying for a degree within the School of Life Sciences.


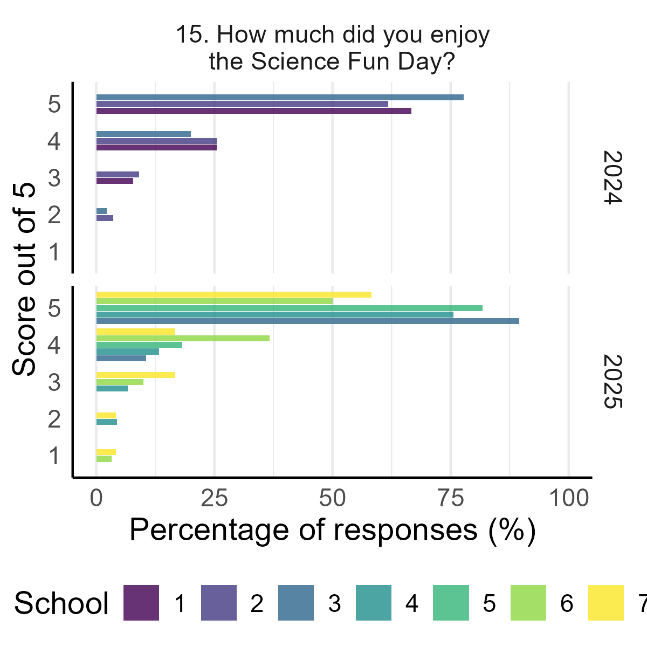

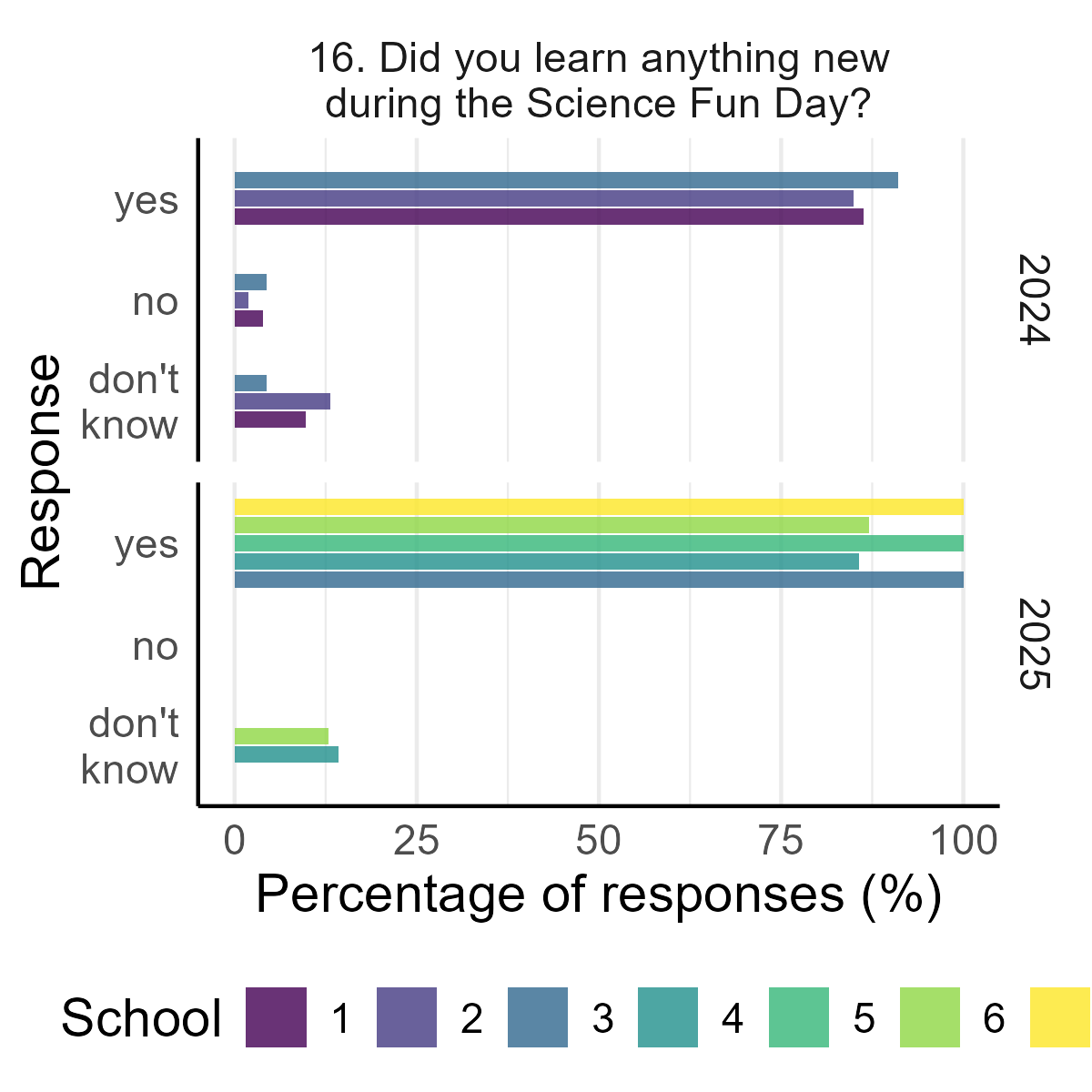

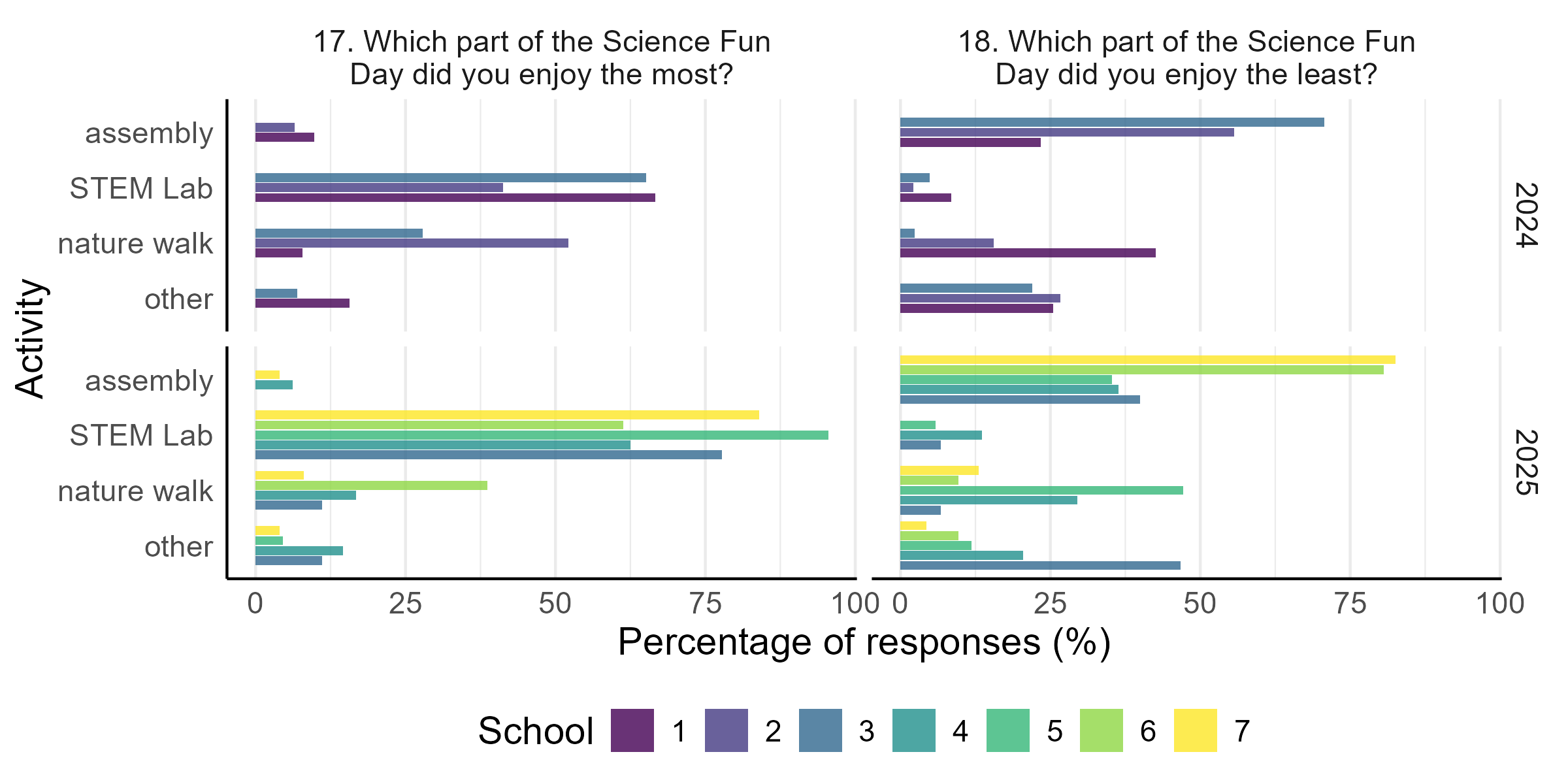


**Figure S 3** Responses to feedback questions from Schools 1 to 7 across 2024 and 2025 Science Fun Days.


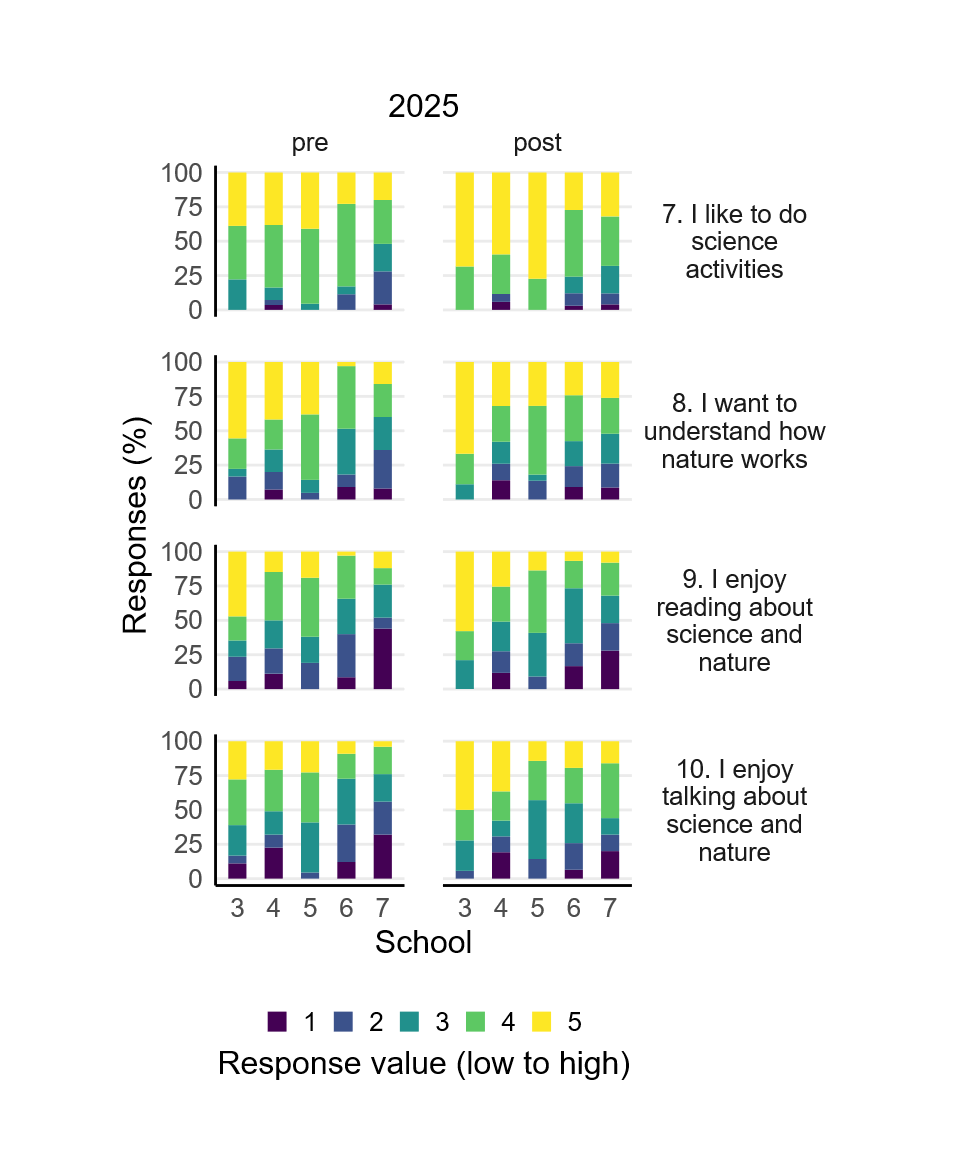

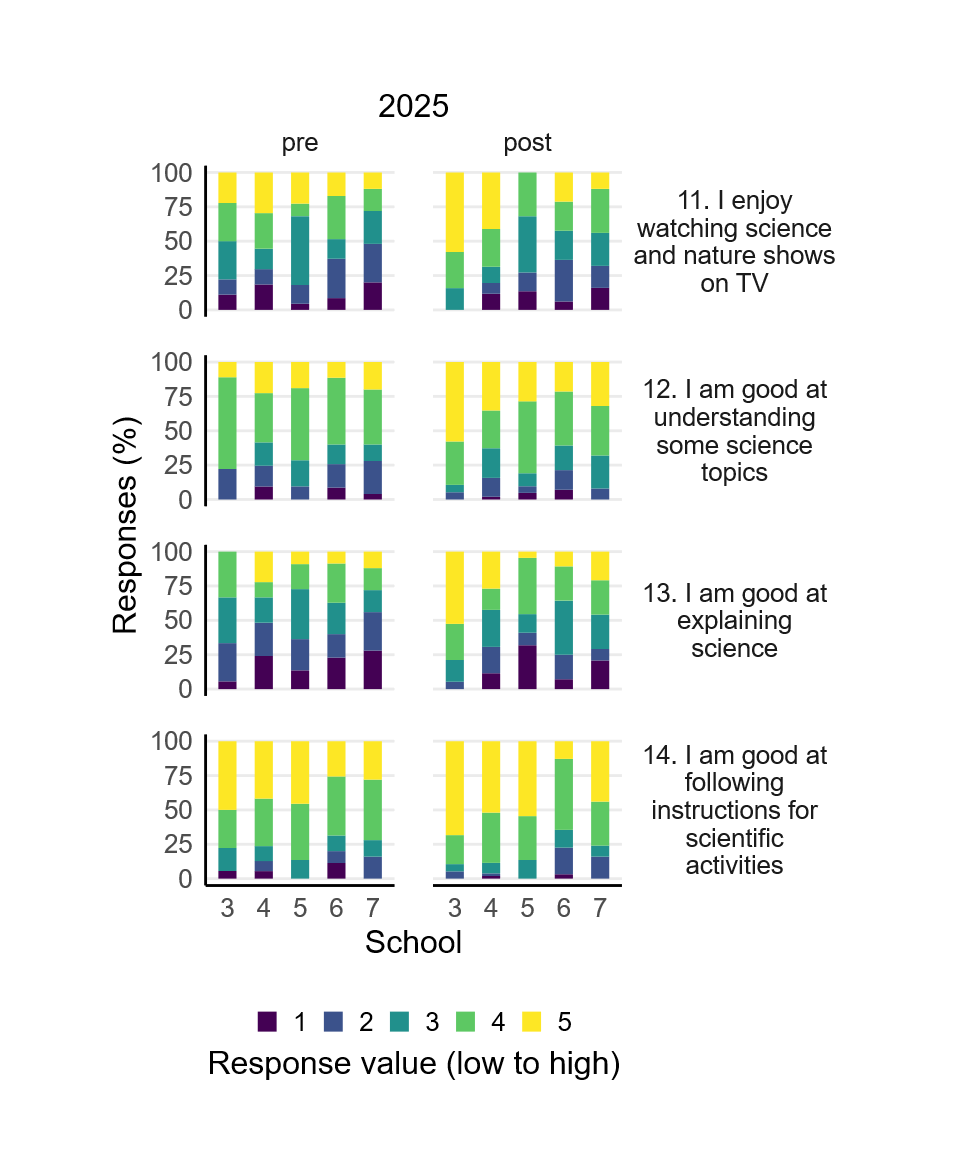


**Figure S 4** Survey results for extra questions in 2025 at the start (pre) and end (post) of the event grouped by School. (Scores on a 5-level Likert scale with 1 being lowest and 5 being the highest.)


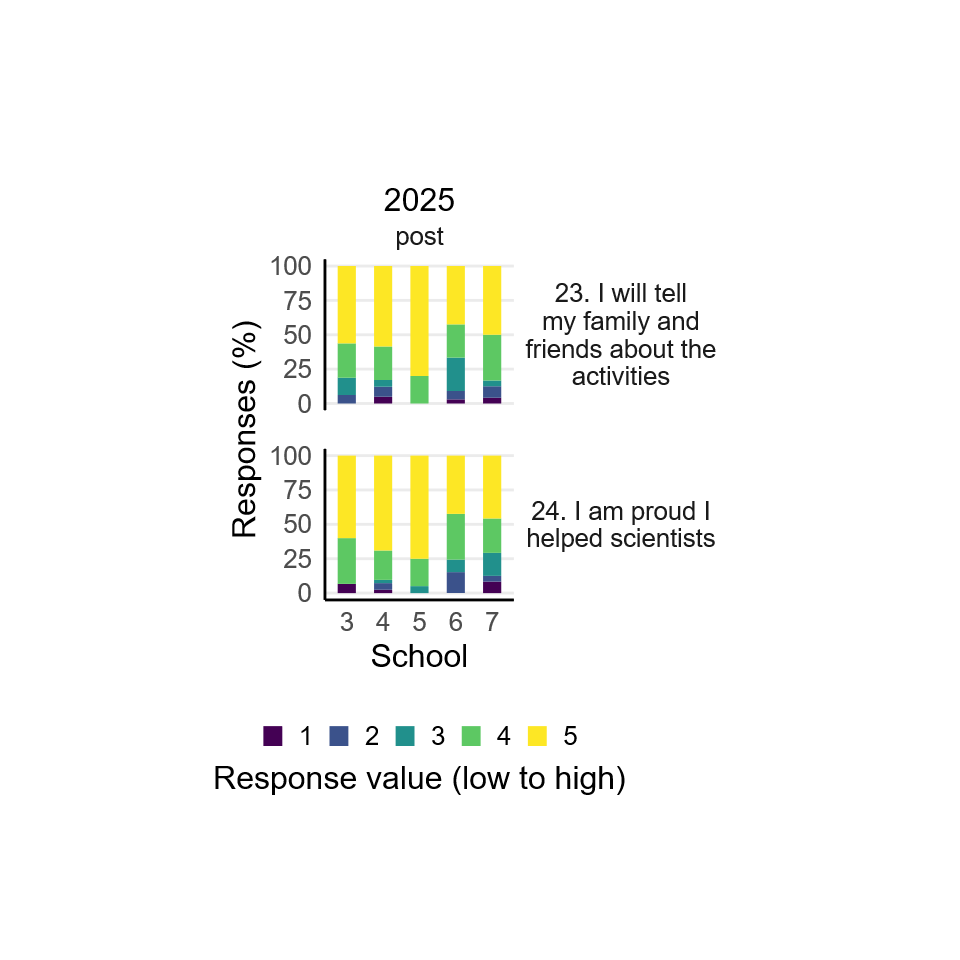

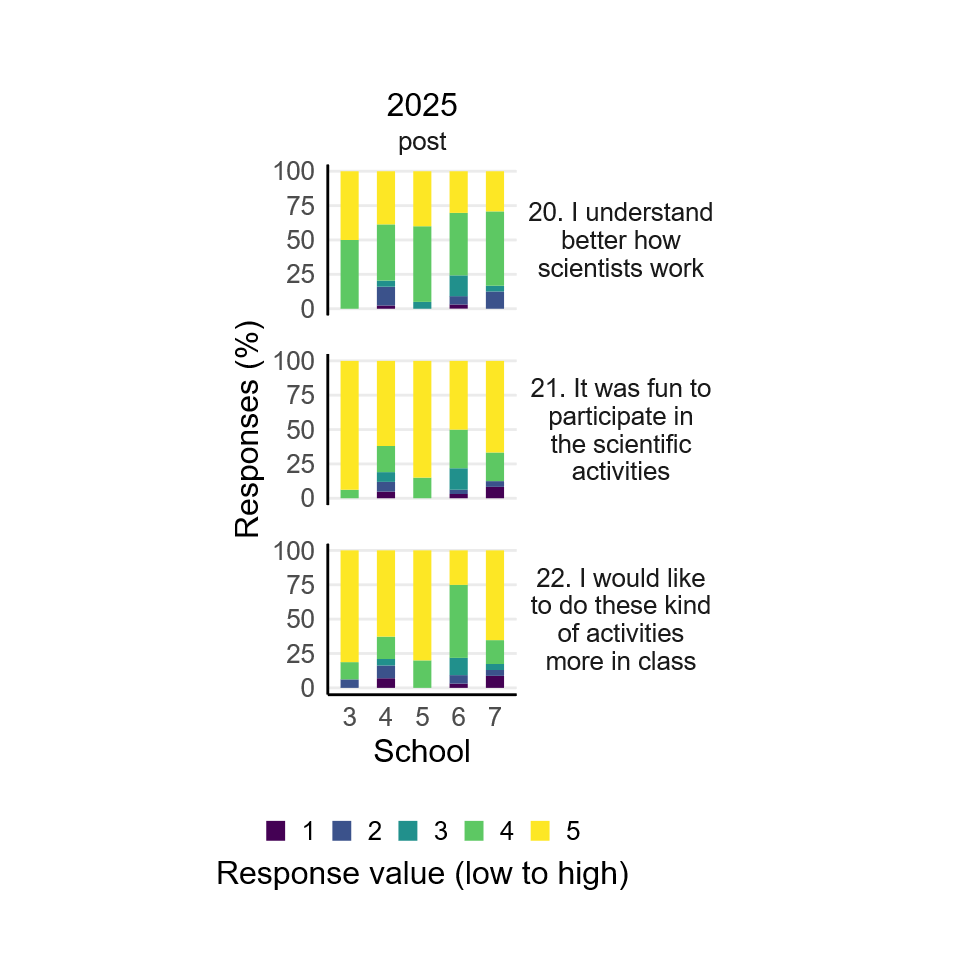


**Figure S 5** Survey results for extra feedback (post-event) questions in 2025 grouped by School. (Scores on a 5-level Likert scale with 1 being lowest and 5 being the highest.)


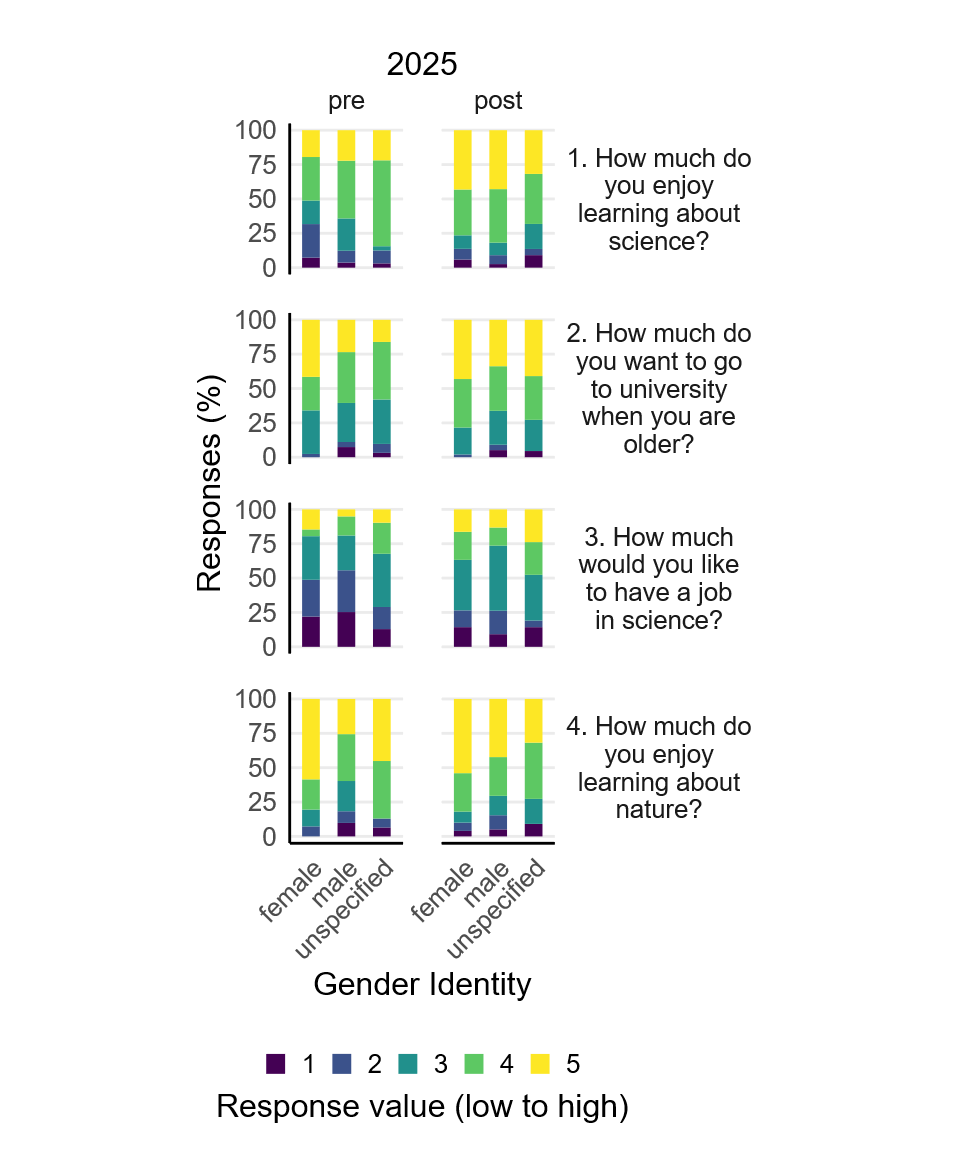


**Figure S 6** Survey results for 2025 at the start (pre) and end (post) of the event grouped by gender identity. (Scores on a 5-level Likert scale with 1 being lowest and 5 being the highest.)


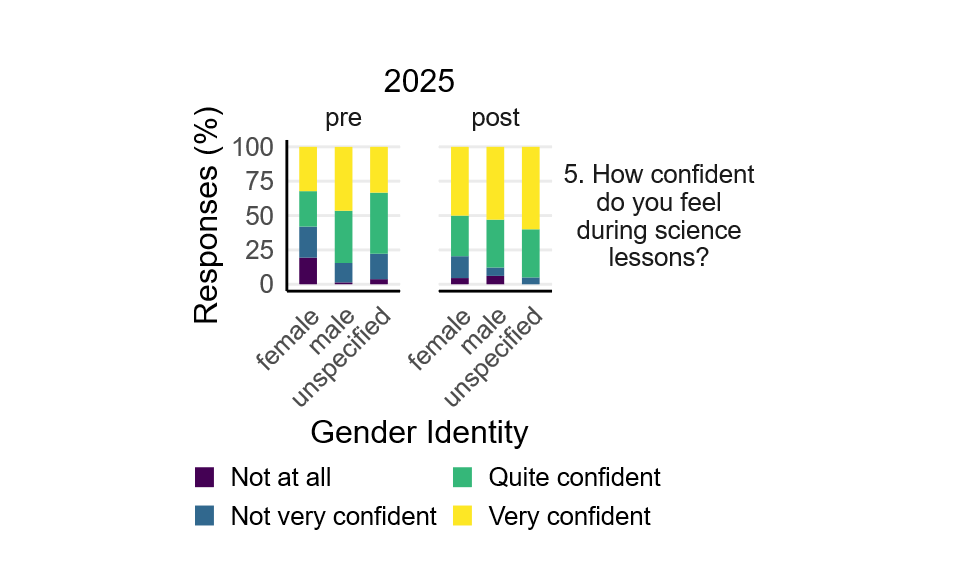

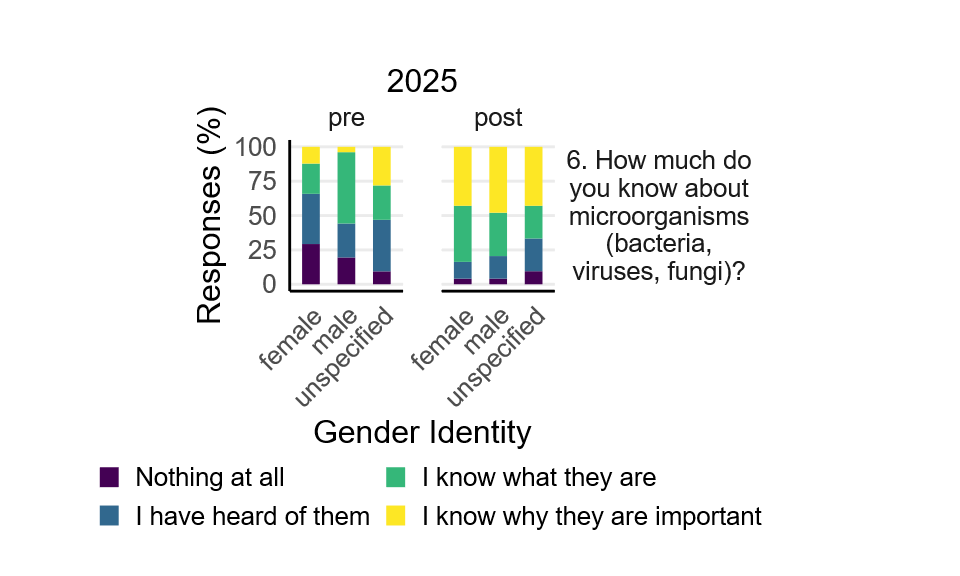


**Figure S 7** Survey results for 2025 at the start (pre) and end (post) of the event grouped by gender identity.


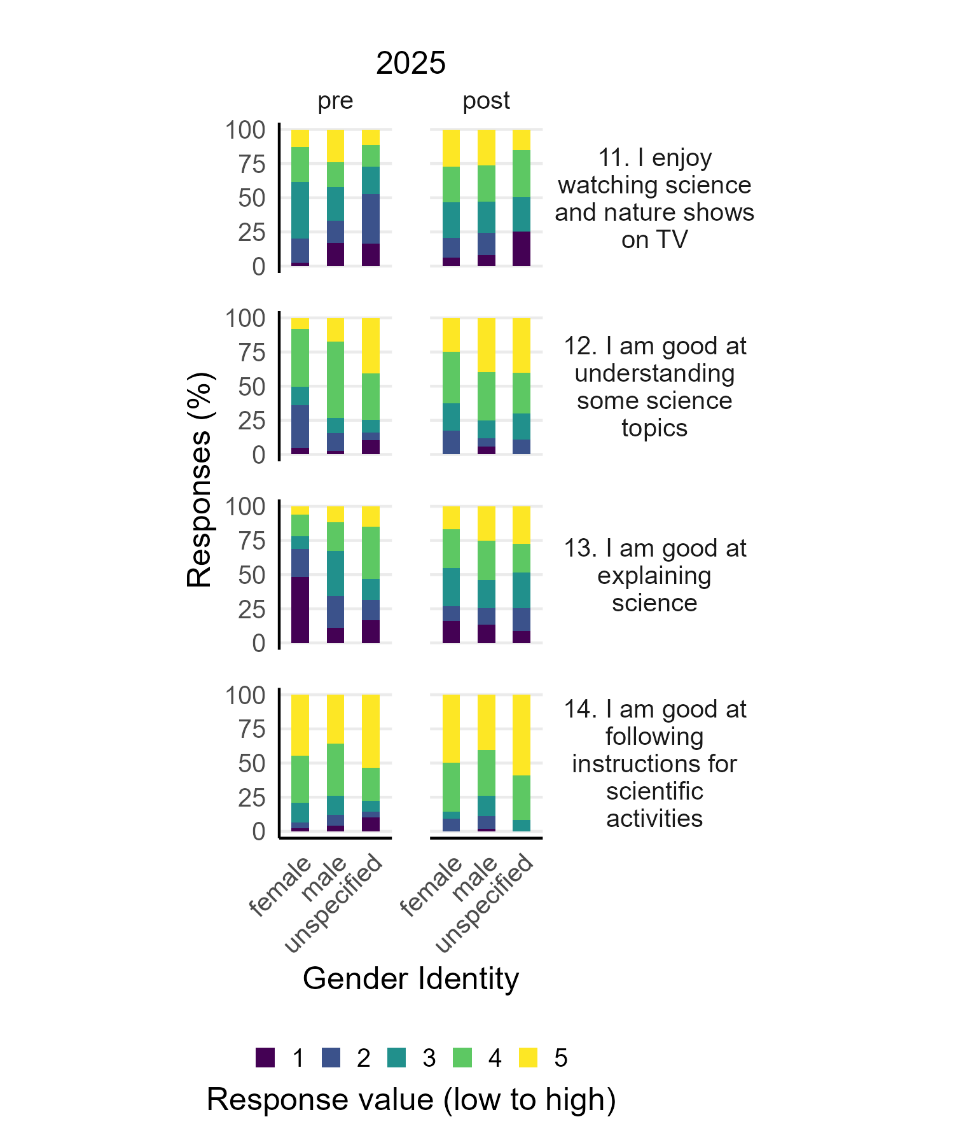

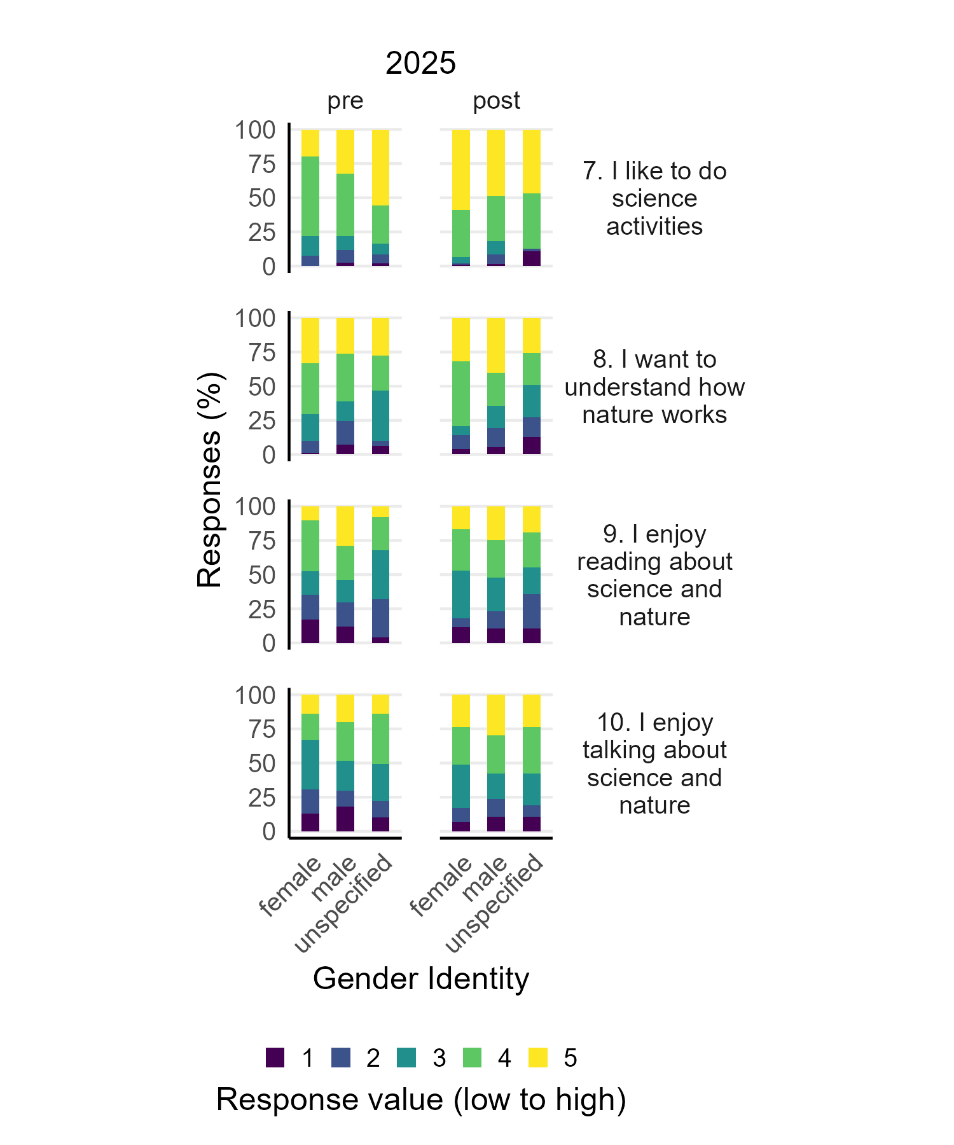


**Figure S 8** Survey results for extra questions in 2025 at the start (pre) and end (post) of the event grouped by Gender Identity. (Scores on a 5-level Likert scale with 1 being lowest and 5 being the highest.)


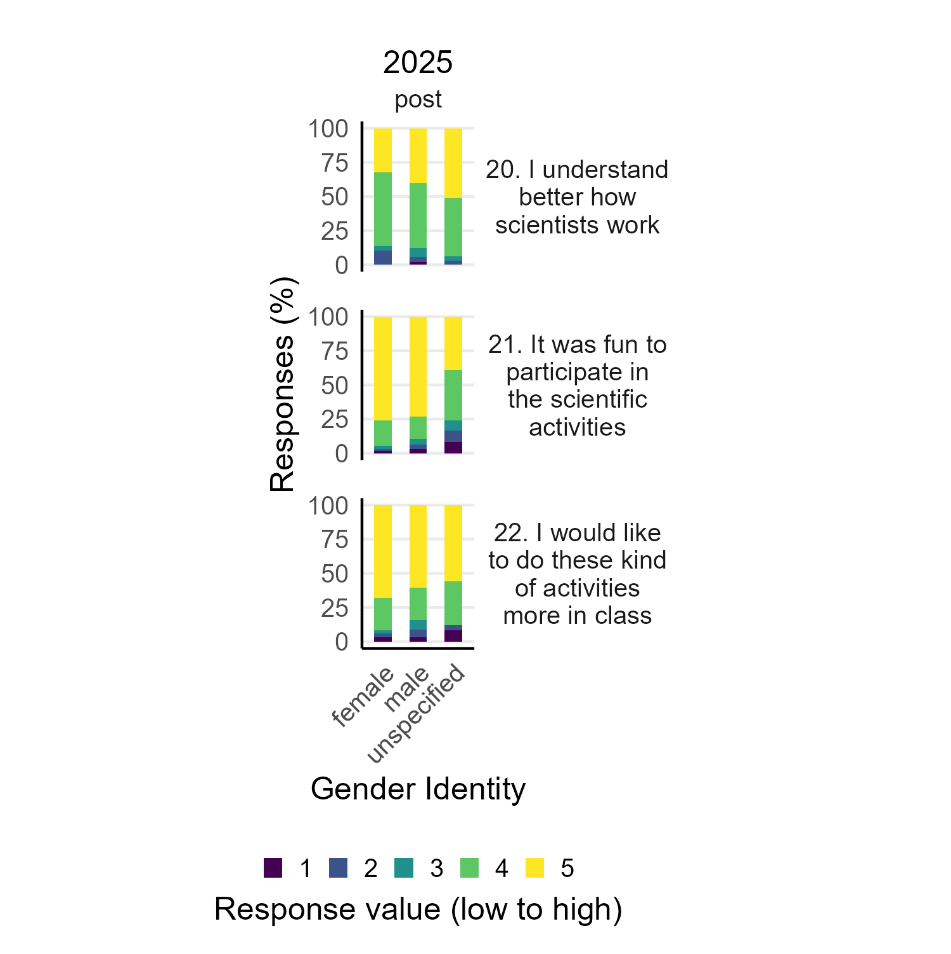

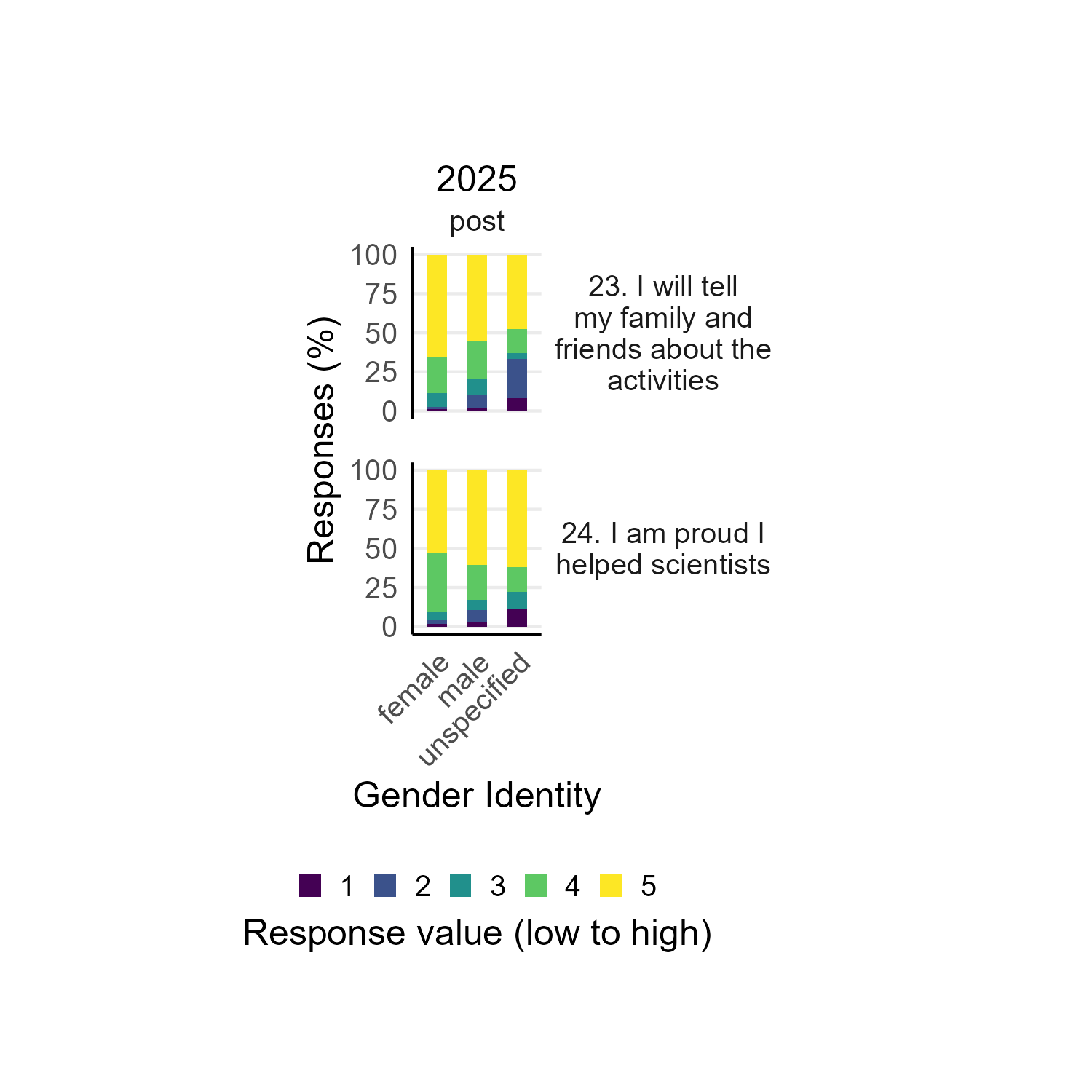


**Figure S 9** Survey results for extra feedback (post-event) questions in 2025 grouped by gender identity. (Scores on a 5-level Likert scale with 1 being lowest and 5 being the highest.)


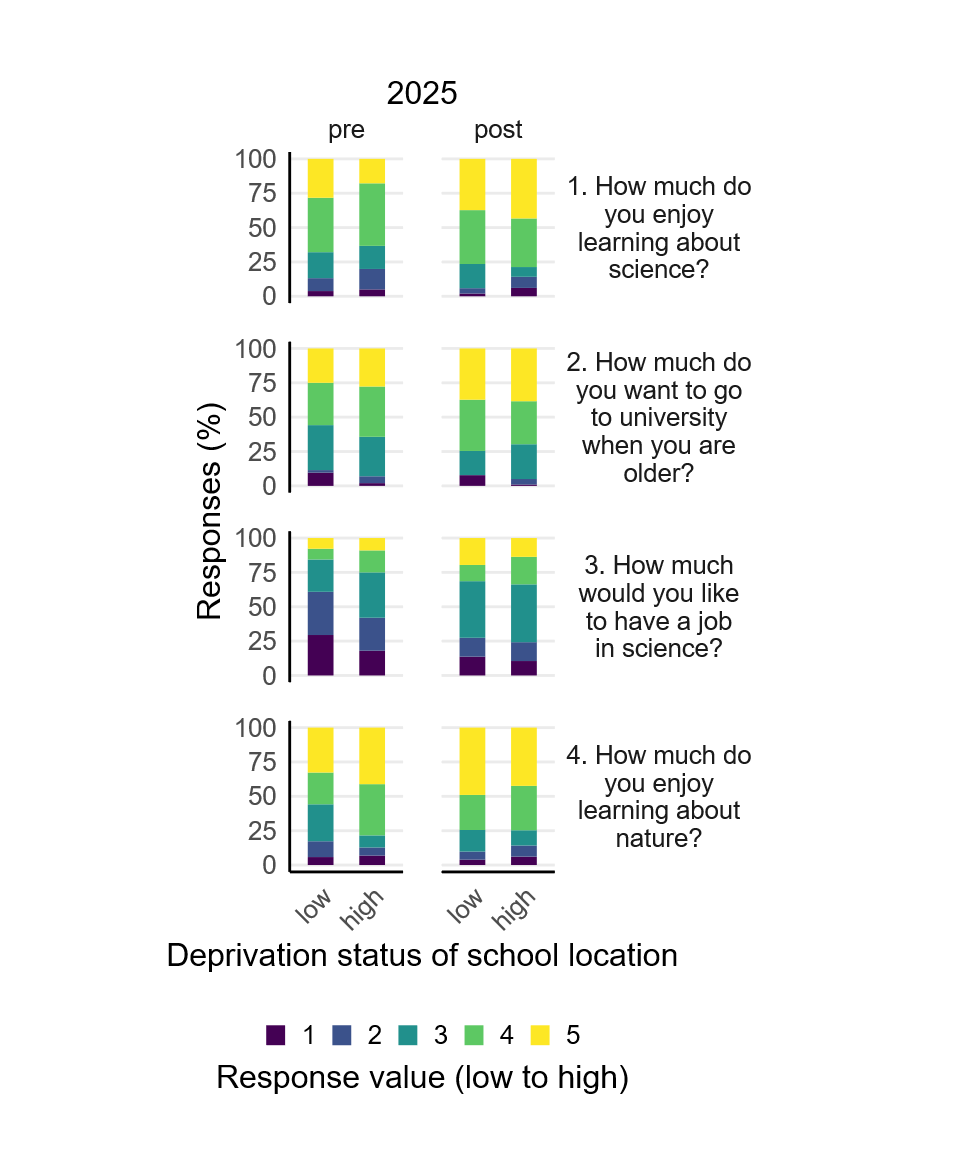


**Figure S 10** Survey results for questions in 2025 at the start (pre) and end (post) of the event grouped by Index of Multiple Deprivation (IMD) decile of school location (high-deprivation-location [IMD 1-5], low-deprivation-location [IMD 6-10]). (Scores on a 5-level Likert scale with 1 being lowest and 5 being the highest.)


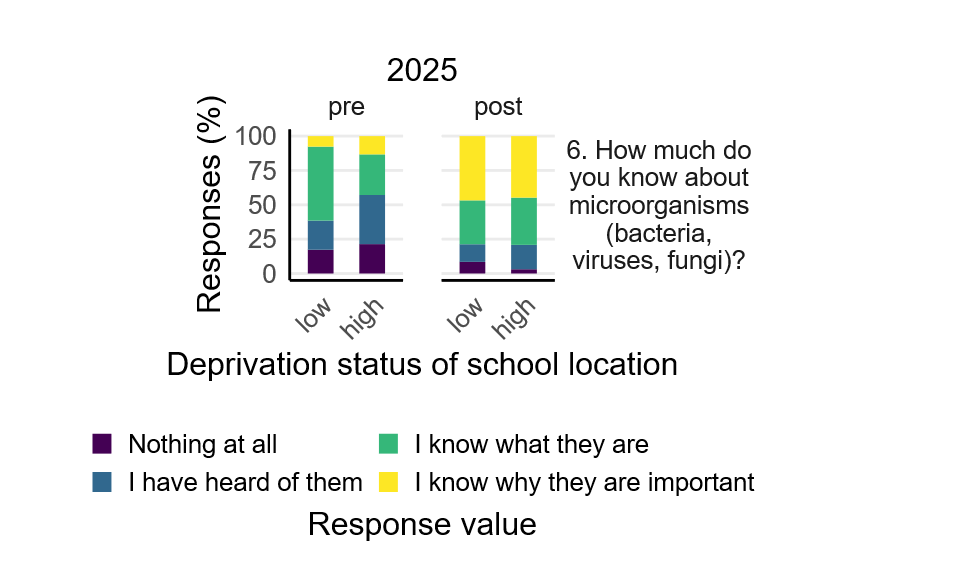

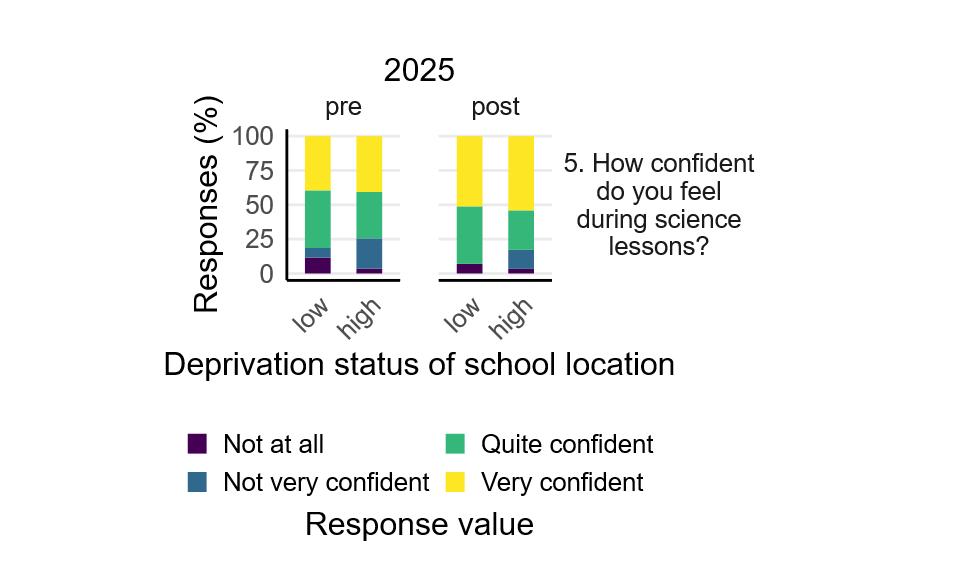


**Figure S 11** Survey results for questions in 2025 at the start (pre) and end (post) of the event grouped by Index of Multiple Deprivation (IMD) decile of school location (high-deprivation-location [IMD 1-5], low-deprivation-location [IMD 6-10]).

**Figure S 12** Survey results for extra questions in 2025 at the start (pre) and end (post) of the event grouped by Index of Multiple Deprivation (IMD) decile of school location (high-deprivation-location [IMD 1-5], low-deprivation-location [IMD 6-10]). (Scores on a 5-level Likert scale with 1 being lowest and 5 being the highest.)


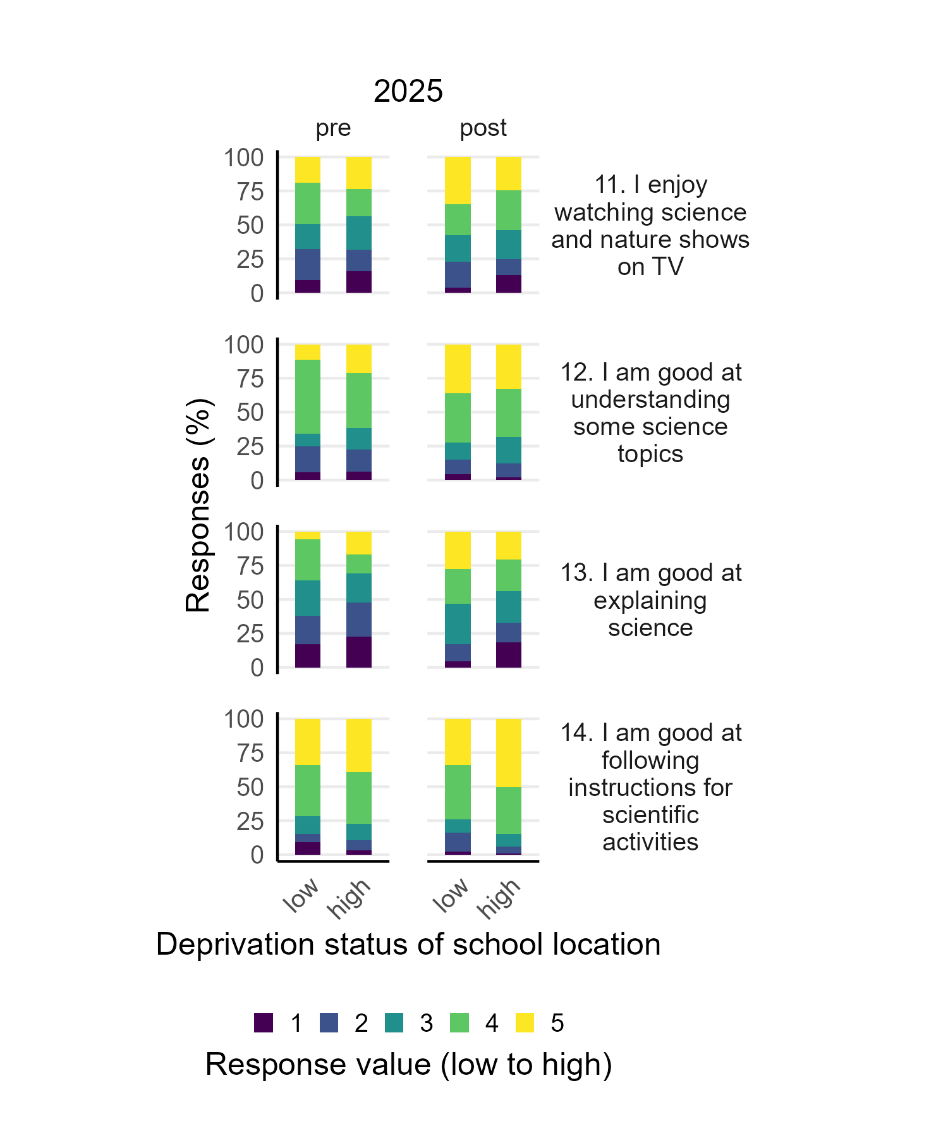

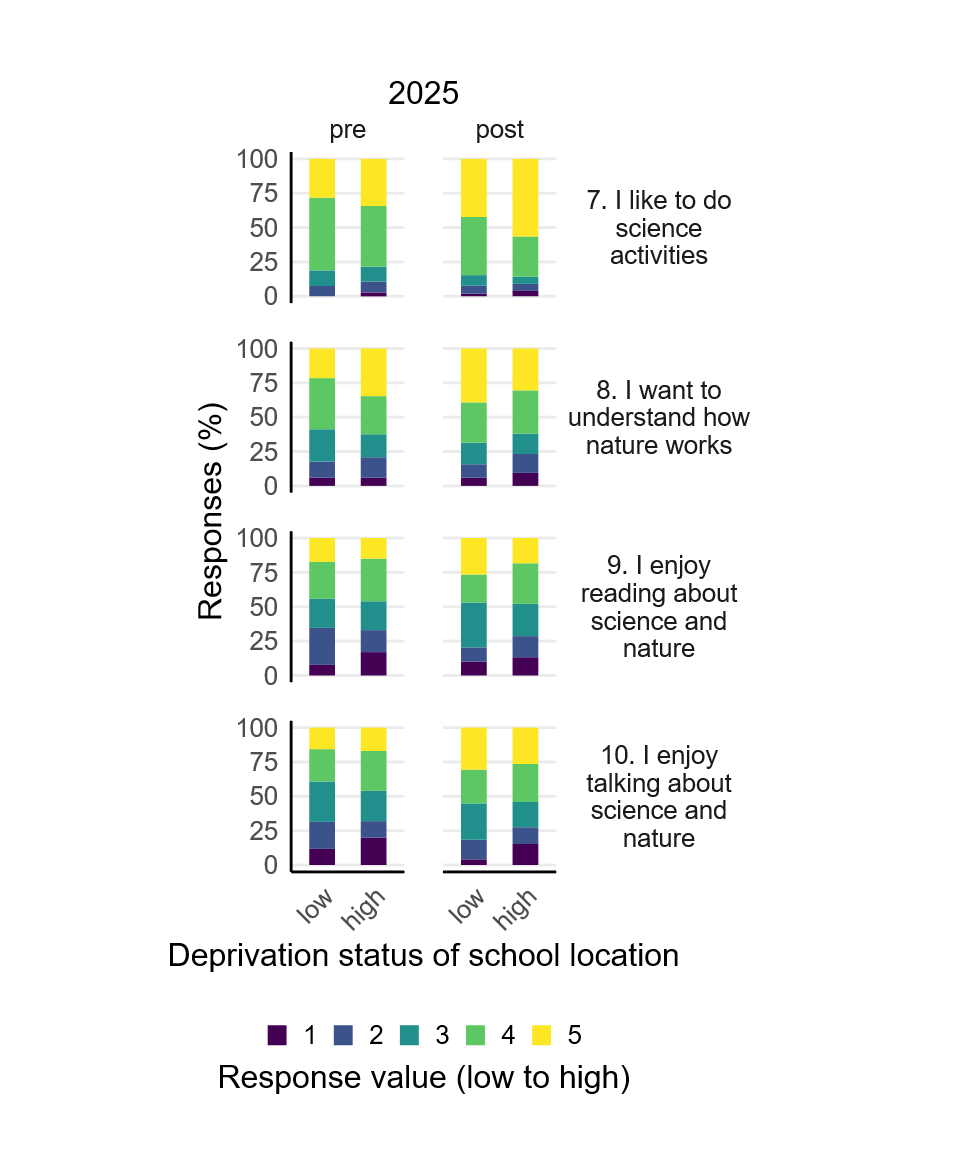

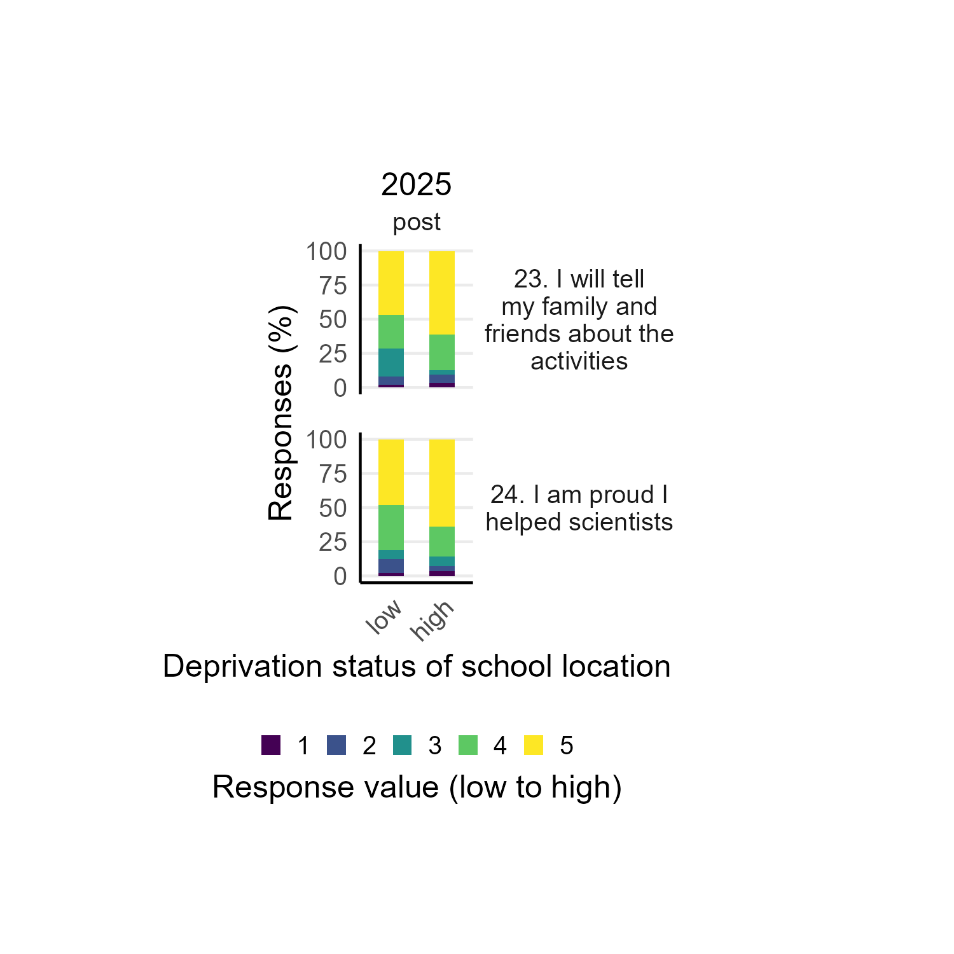

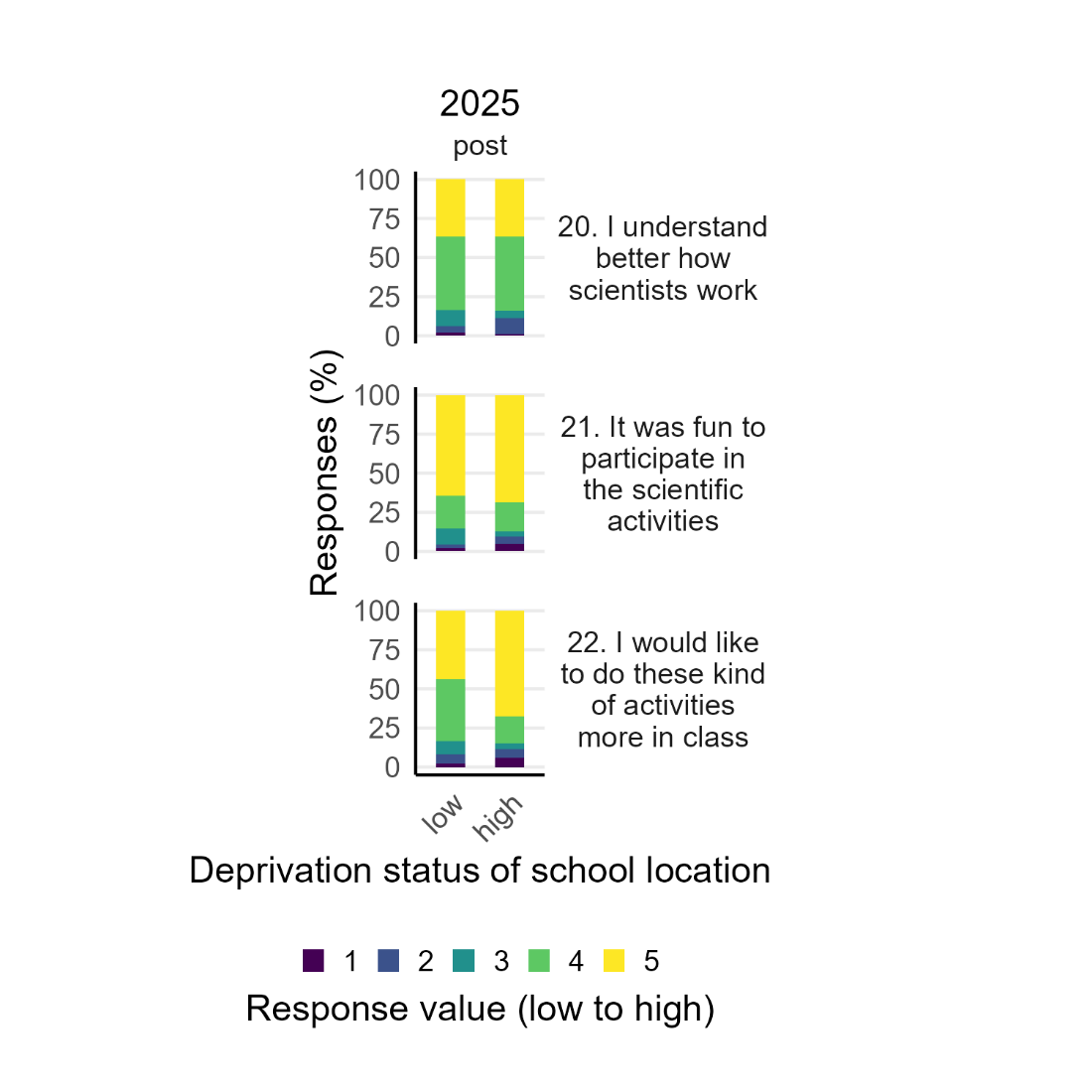


**Figure S 13** Survey results for extra questions in 2025 at the start (pre) and end (post) of the event grouped by Index of Multiple Deprivation (IMD) decile of school location (high-deprivation-location [IMD 1-5], low-deprivation-location [IMD 6-10]). (Scores on a 5-level Likert scale with 1 being lowest and 5 being the highest.)

### Classroom bioaerosol sampling

In parallel with the Science Fun Days, the University of Essex was conducting air sampling in classrooms of some of the schools involved (participation anonymous) as part of the InChildHealth project (grant agreement: EU:101056883, UKRI: 10040524). Students were given the opportunity to take their own bioaerosol samples in locations of their choice in their school. To facilitate this, their teachers were given sampling packs containing a protocol and data-recording sheet (see attached file), three electrostatic dust collectors (EDCs) (13.5 x 10 cm cloth stapled to half A4 size paper) (see Figure S10), and three 50 mL centrifuge tubes containing 20 mL 0.9% sodium chloride + 0.05% Tween 80 solution. These packs were provided in postage-paid self-addressed packages. Pupils and teachers were instructed to note where they set out the EDCs in classrooms for 30 days before, using the gloves provided, placing the cloths in the centrifuge tubes and shake for 1 min (as they did at the Science Fun Day). Following this, the samples were posted back to the University of Essex, where they were analysed for viable fungal (dichloran glycerol agar, DG18) and bacterial (tryptic soy agar, TSA) counts. On arrival at the University of Essex the samples were stored at 4°C for up to four days. The tubes containing the cloths were shaken at 250 rpm for 30 min, then 100 µL of the liquid extract was pipetted onto petri dishes containing DG18 or TSA and incubated at 37°C for 48 hours before counting.


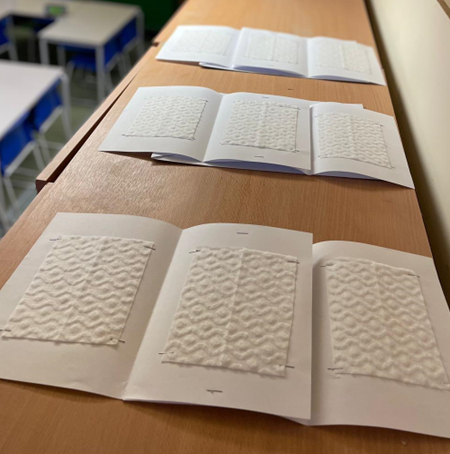


**Figure S 14** Electrostatic dust collectors (EDCs) like those given to schools for classroom bioaerosol sampling.

### Marine activities (full description)

Pupils were shown a shallow 10L seawater tank with green crabs (*Carcinus maenas*), periwinkles (*Littorina littorea*), beadlet anemones (*Actinia equina*), oysters (*Magallana gigas*) and macroalgae (e.g., *Ulva Lactuca, Fucus spiralis*) so they could get close to and learn about their local marine species. They were also shown a grey gurnard (*Eutrigla gurnardus)* caught locally, and encouraged to identify and discuss how morphological adaptations help it to survive; e.g., spines and camouflage to reduce predation, and its separated pectoral rays to allow it to ‘walk’ over the seabed to find benthic prey such as worms and crabs.

Pupils were then split into two small groups. The first group was given fish otoliths (earstones) to hold in their hands and told how important these little ‘bones’ are to avoid overfishing by allowing scientists to check that humans are not removing too many of the oldest or youngest fish from the sea. Each pupil looked at a sectioned 6-year old cod (*Gadus morhua*) otolith under a dissecting microscope and tried to count the annual growth rings (just like tree trunk rings) and we had a mini ‘competition’ to see who was closest to the actual age.

The second group was taken to play the ‘Wheel of Misfortune’ to learn about the perils faced by larval and juvenile fish, with often only 1 in 100 surviving to their first birthday. The main goal of this activity was to encourage the pupils to make the link that the fish that they eat may have grown up in their local river, beach or estuary, and for us to brainstorm how we can improve the health of these ‘nursery habitats’ to help more fish survive. The game began with a home-made LEGO ‘Plinko board’, where the pupils would each release a metal marble – representing a fish egg or larva - from the ‘offshore spawning grounds’ at the top of the board to a ‘safe beach’ at the bottom of the board (channels demarked by a smiley face) (see Figure S 10). We made the probability of reaching a beach 50:50 (which is biologically far too high) to ensure that some pupils could move onto the second stage. In reality, we let them keep trying until they ‘reached a beach’ to avoid disappointment. For pupils whose eggs ‘survived’, they then spun the Wheel of Misfortune to see which number they landed on (1 to 50). Only segment 1 resulted in their fish surviving to its first birthday. The remaining 49 resulted in mortality via an even that was revealed by them lifting a numbered flap. These events were suggested by scientists around the world via a social media campaign, and ranged from natural and unavoidable factors such as predation (‘Eaten by a hungry gannet’) and mismatch theory (‘Swept offshore and could not find shelter’) to factors associated with climate change (‘Heat exhaustion during a marine heatwave’), noise and chemical pollution (‘Poisoned by an oil spill’) and other anthropogenic factors (e.g. ‘Unintended capture in fishing nets (bycatch)’). The outcomes were all realistic, but written in a child-friendly and humorous way, with the focus being to introduce the pupils to the serious problems of habitat loss and anthropogenic disturbances, both locally and globally, and the need to change our behaviour and energy generation methods, and to remediate and restore these habitats to protect the fish and other animals that rely on them. After every pupil had had a chance to spin the wheel, the two groups swapped.

**a)**


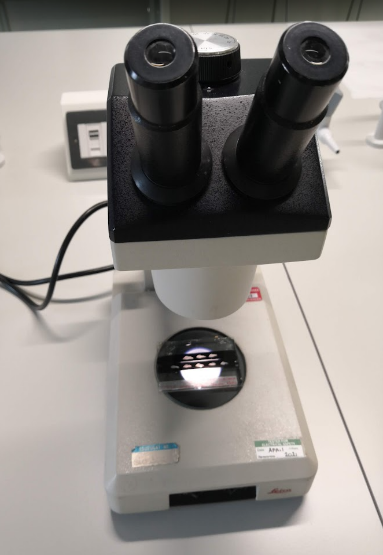

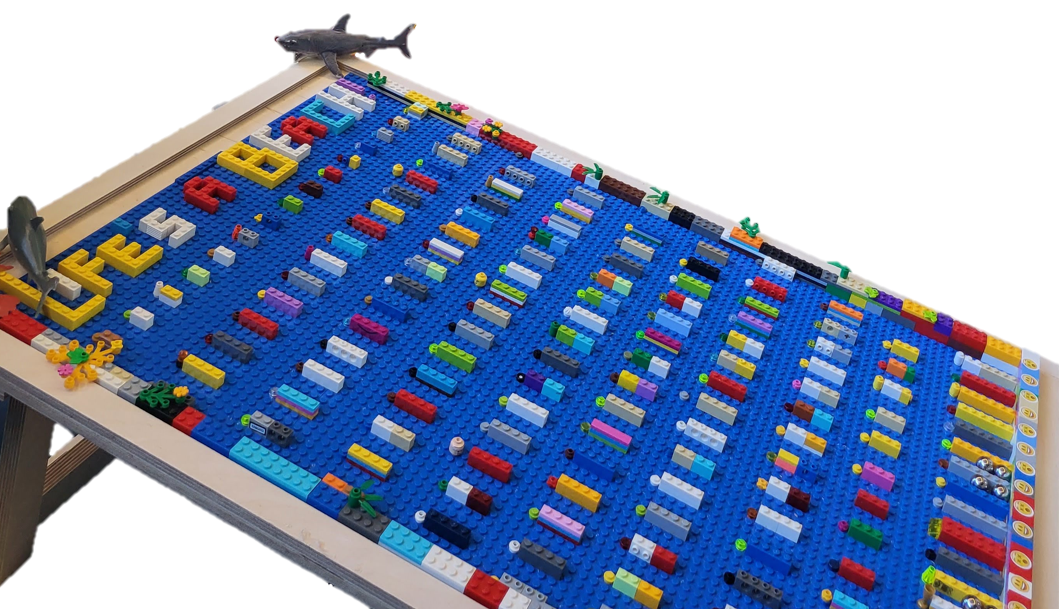


**b)**

**Figure S 15** **a)** LEGO ‘plinko’ board where the pupils would each release a metal marble – representing a fish egg or larva - from the ‘offshore spawning grounds’ at the top of the board to a ‘safe beach’ at the bottom of the board (channels demarked by a smiley face). **b)** Pupils looked at a sectioned 6-year-old cod (*Gadus morhua*) otolith (fish ‘earstone’) under a dissecting microscope and counted the annual growth rings (just like tree trunk rings).

The 50 events on the wheel of misfortune:

| **Options** | **Category** |
| --- | --- |
| Eaten by my own cousin! (cannibalism by older age class) | Predation |
| Confused by noise pollution from oil drilling and got lost | Humans |
| Suffocate when the water dried up during a drought | Climate change |
| Heat exhaustion during a marine heatwave | Climate change |
| Swept offshore and couldnt find a home | Habitat |
| Red tide! Sick from eating too much toxic algae | Habitat |
| Poisoned by heavy metals (industrial waste) | Humans |
| Scooped up by a digger taking gravel out the river | Habitat |
| Squashed by an anchor | Humans |
| Met a hungry cormorant. | Predation |
| Heron attack! | Predation |
| Genetic mutation caused by inbreeding (overfished population) | Humans |
| Eaten by my mum! | Predation |
| Infested by sea lice from a nearby salmon farm | Humans |
| Extreme rains caused a landslide that smothered my home | Climate change |
| Mama's yolk was lovely but it ran out before I could find anything to eat | Habitat |
| Ingested toxic substances after a chemical spill | Humans |
| Suffocated by a wave of anoxic waters | Habitat |
| Sucked into a jetski engine. | Humans |
| Stressed after acid rain lowered the pH level of the water | Humans |
| Eaten by an exotic aquarium fish someone flushed down their loo | Predation |
| Starved after hatching too early in the season (lack of food) | Habitat |
| Suffocated while stuck in a methane bubble | Habitat |
| Unintended capture in fishing nets (bycatch) | Humans |
| Summer was nice but froze to death in a cold snap | Climate change |
| New species moved into my home due to warming and ate me! | Climate change |
| Streetlights lit me up at night and I got eaten! | Humans |
| Met a hungry seal | Predation |
| Caught by a lecturer for undergrad fish ID course | Humans |
| Weak, slow growing and hungry - an easy meal for a bass | Predation |
| Trapped in a drying out tidepool | Habitat |
| Met a hungry dragonfly larva | Predation |
| Sucked into a water pump and sprayed over crops | Humans |
| Swept offshore and couldn't find shelter | Habitat |
| Dissected by a scientist to see what I'd eaten | Humans |
| Starved after a big storm moved away my food patch | Climate change |
| Met a hungry pike | Predation |
| Starved after getting "full" eating microplastics | Habitat |
| Poisoned and suffocated by an oil spill | Humans |
| Eaten because my seagrass home was destroyed by boats | Habitat |
| Got lost trying to reach the ocean due to river diversions | Habitat |
| Poisoned by flame retardants | Humans |
| New species moved into my home due to warming and ate all my food | Climate change |
| Several tonnes of human faeces just landed on my house | Habitat |
| Infected by a parasite that made me swim at the surface. Wham! Heron attack! | Predation |
| Smothered after an earthquake caused a landslide | Habitat |
| Suffocated after being blown onto the beach by a sudden tornado | Climate change |
| Suffocated after fertiliser & sewage spills caused eutrophication and hypoxia | Humans |
| Blown up by dynamite fishing | Humans |
| **CONGRATULATIONS - YOU JUST SURVIVED TO YOUR FIRST BIRTHDAY!  CAN YOU SURVIVE A FEW MORE YEARS TO MAKE YOUR OWN BABIES?** |  |
